# International surveillance study in acute spinal cord injury confirms viability of multinational clinical trials

**DOI:** 10.1186/s12916-022-02395-0

**Published:** 2022-06-14

**Authors:** Lucie Bourguignon, Bobo Tong, Fred Geisler, Martin Schubert, Frank Röhrich, Marion Saur, Norbert Weidner, Rüdiger Rupp, Yorck-Bernhard B. Kalke, Rainer Abel, Doris Maier, Lukas Grassner, Harvinder S. Chhabra, Thomas Liebscher, Jacquelyn J. Cragg, EMSCI study group, John Kramer, Armin Curt, Catherine R. Jutzeler

**Affiliations:** 1 Department of Health Sciences and Technology (D-HEST), ETH Zurich, Zürich, Switzerland; 2 SIB Swiss Institute of Bioinformatics, Lausanne, Switzerland; 3 International Collaboration on Repair Discoveries (ICORD), University of British Columbia, Vancouver, Canada; 4 University of Saskatchewan, Saskatoon, Saskatchewan Canada; 5 Spinal Cord Injury Center, University Hospital Balgrist, University of Zurich, Lengghalde 2, 8006 Zürich, Switzerland; 6 Berufsgenossenschaftliche Klinik Bergmanstrost of Halle, Halle, Germany; 7 Orthopädische Klinik, Hessisch Lichtenau, Germany; 8 Spinal Cord Injury Center, Heidelberg University Hospital, Heidelberg, Germany; 9 Spinal Cord Injury Center Orthopaedic Department, Ulm University, Ulm, Germany; 10 Spinal Cord Injury Center, Bayreuth, Germany; 11 Spinal Cord Injury Center, Trauma Center Murnau, Murnau, Germany; 12 Department of Neurosurgery, Medical University Innsbruck, Innsbruck, Austria; 13 Spine Service, Indian Spinal Injuries Centre, Sector C, Vasant Kunj, New Delhi, India; 14 Treatment Centre for Spinal Cord Injuries, Trauma Hospital Berlin, Berlin, Germany; 15 Collaboration for Outcomes Research and Evaluation (CORE), Faculty of Pharmaceutical Sciences, University of British Columbia, Vancouver, Canada; 16 Djavad Mowafaghian Centre for Brain Health, University of British Columbia, Vancouver, Canada; 17 Department of Anesthesiology, Pharmacology, and Therapeutics, Faculty of Medicine, University of British Columbia, Vancouver, Canada

**Keywords:** Spinal cord injury, Surveillance study, Neurological recovery, Functional recovery, Aging, Epidemiological shift, Benchmark

## Abstract

**Background:**

The epidemiological international landscape of traumatic spinal cord injury (SCI) has evolved over the last decades along with given inherent differences in acute care and rehabilitation across countries and jurisdictions. However, to what extent these differences may influence neurological and functional recovery as well as the integrity of international trials is unclear. The latter also relates to historical clinical data that are exploited to inform clinical trial design and as potential comparative data.

**Methods:**

Epidemiological and clinical data of individuals with traumatic and ischemic SCI enrolled in the European Multi-Center Study about Spinal Cord Injury (EMSCI) were analyzed. Mixed-effect models were employed to account for the longitudinal nature of the data, efficiently handle missing data, and adjust for covariates. The primary outcomes comprised demographics/injury characteristics and standard scores to quantify neurological (i.e., motor and sensory scores examined according to the International Standards for the Neurological Classification of Spinal Cord Injury) and functional recovery (walking function). We externally validated our findings leveraging data from a completed North American landmark clinical trial.

**Results:**

A total of 4601 patients with acute SCI were included. Over the course of 20 years, the ratio of male to female patients remained stable at 3:1, while the distribution of age at injury significantly shifted from unimodal (2001/02) to bimodal distribution (2019). The proportional distribution of injury severities and levels remained stable with the largest percentages of motor complete injuries. Both, the rate and pattern of neurological and functional recovery, remained unchanged throughout the surveillance period despite the increasing age at injury. The findings related to recovery profiles were confirmed by an external validation cohort (*n*=791). Lastly, we built an open-access and online surveillance platform (“*Neurosurveillance*”) to interactively exploit the study results and beyond.

**Conclusions:**

Despite some epidemiological changes and considerable advances in clinical management and rehabilitation, the neurological and functional recovery following SCI has remained stable over the last two decades. Our study, including a newly created open-access and online surveillance tool, constitutes an unparalleled resource to inform clinical practice and implementation of forthcoming clinical trials targeting neural repair and plasticity in acute spinal cord injury.

**Supplementary Information:**

The online version contains supplementary material available at 10.1186/s12916-022-02395-0.

## Background

Traumatic spinal cord injury is a devastating neurological disorder that is associated with life-long neurological condition with motor, sensory, and autonomic deficits [1]. Damage to the spinal cord occurs via both mechanical perturbation (so-called primary injury) and a cascade of damaging pathophysiological events (so-called secondary injury) [2, 3]. There are no pharmacological or non-pharmacological interventions available that mitigate the extent of damage in the acutely injured spinal cord. Despite the lack of effective treatment options, considerable progress has been made toward reducing the mortality rate and morbidity among patients with spinal cord injury [4, 5]. This progress is chiefly attributable to advances in the acute and long-term care of spinal cord injury, including early spine surgery (i.e., decompression and stabilization) [6], blood pressure augmentation within the first week post injury [7], introduction of antibiotics [8], availability of specialized rehabilitation centers [9], rehabilitation practices (e.g., gait training), and the prevention and treatment of secondary complications (e.g., infections and neuropathic pain) [10, 11].

Little is known about the impact of these advances on the rate and pattern of functional and neurological recovery following traumatic spinal cord injury. This knowledge gap is partially attributable to the data sources available, which are often limited in consistency and sample size, lack follow-up measures, and/or non-standardized data collection [12]. Various recent studies have reported changes in demographics and injury characteristics over the past decades. Most of these, however, have focused on regional epidemiology for a limited number of outcome measures, spanning only a relatively short time period [13, 14]. There is a paucity of validated long-term and comprehensive longitudinal studies.

Our study addressed this knowledge gap by leveraging data from the European Multi-center Study About Spinal Cord Injury (EMSCI)—the largest and most comprehensive longitudinal international data source in the field of spinal cord injury (https://www.emsci.org/). The first aim was to investigate changes in the epidemiological landscape of traumatic spinal cord injury over the last 20 years with a focus on changes in demographics and geographical and injury characteristics. Based on previous evidence [13, 14], we hypothesized a shift to older and less severe injuries along with an invariable ratio of female to male patients. The second aim was to establish a benchmark for the rate and pattern of neurological and functional recovery after a spinal cord injury. To this end, we investigated the extent that functional and neurological recovery following traumatic spinal cord injury has changed over the last two decades. We hypothesized that changes in acute and rehabilitation practices have led to improved outcomes during the transition from acute to chronic spinal cord injury. External validation was conducted using data from a landmark clinical trial.

Lastly, we developed the *Neurosurveillance* web platform for the spinal cord injury community, researchers, authorities, and policymakers that offers an open-access resource for benchmarking recovery and inform the design and implementation of clinical trials.

## Methods

### Study design and data source

We performed a prospective and longitudinal observational cohort study of individuals enrolled in the EMSCI (https://www.emsci.org, ClinicalTrials.gov Identifier: NCT01571531). The design and reporting of this study adhere to the STROBE guidelines for observational studies [15]. Founded in 2001, the EMSCI comprises 30 participating trauma and rehabilitation centers from across Europe and India that have collected data from more than 5000 individuals with spinal cord injury. Detailed neurological and functional outcomes are comprehensively tracked in individuals with traumatic or ischemic spinal cord injuries at fixed time points over the first year of injury (i.e., very acute [within 2 weeks], acute I [4 weeks], acute II [3 months], acute III [6 months], and chronic [12 months]). Further details on the EMSCI study (e.g., inclusion and exclusion criteria, active centers per year) can be found in Additional file 1: Table S1.

### Cohort definition: inclusion and exclusion criteria

**Fig. 1:**
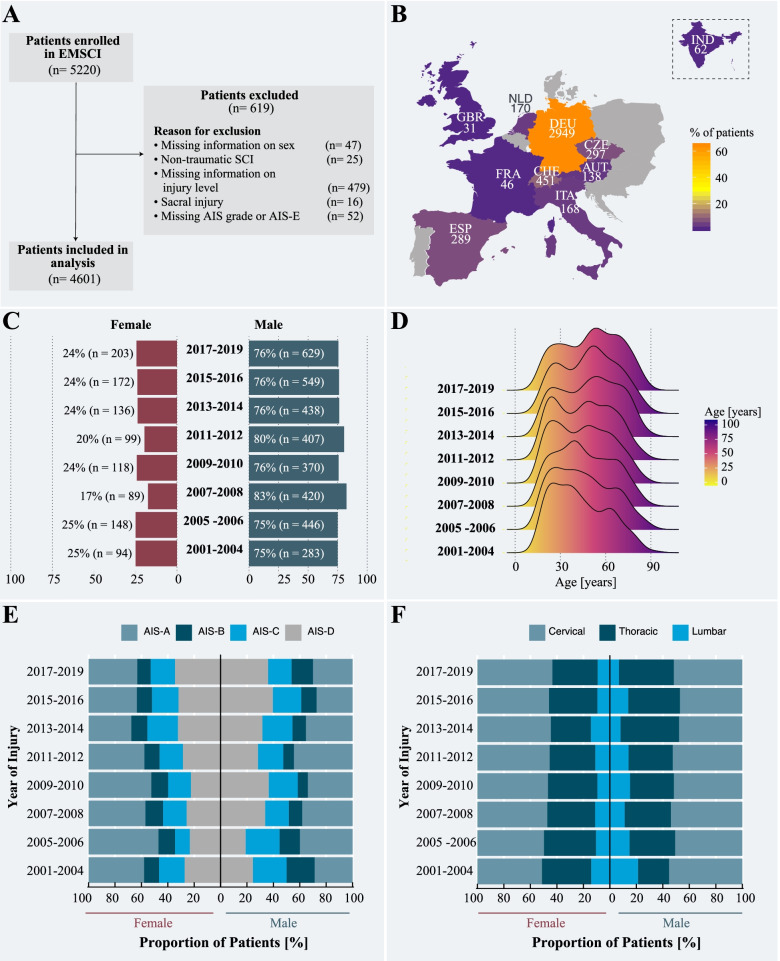
Study overview and result from the main cohort. **A** Flowchart of the included and excluded patients that were originally enrolled in the European Multi-Center Study about Spinal Cord Injury (EMSCI) study. Almost 90% of the EMSCI patients met our inclusion criteria. **B** Number of patients recruited between 2001 and 2019 per country. The majority of patients were admitted to centers in Germany, Switzerland, and Czech Republic. Note: The Indian center joined the EMSCI network only in 2011. **C** Annual ratio between female and male individuals with spinal cord injury enrolled in the EMSCI. Between 2001 and 2019, the ratio between men and women sustaining a traumatic or ischemic spinal cord injury remained comparable at 3:1. **D** Change in distribution of age at injury. Over the last two decades, a shift in age at injury was observed for individuals with spinal cord injury. In comparison to early 2000s, which were characterized by a unimodal distribution, the proportion of elderly people sustaining a traumatic spinal cord injury increased significantly. **E** Baseline injury severity. While there are some fluctuations, the proportions of injury severities, as measured by AIS scores, remained constant across the study period. **F** Baseline level of injury. The proportion of cervical, thoracic, and lumbar injuries did not significantly change as a function of time

To be included in our study, patients enrolled in the EMSCI had to meet the following inclusion criteria: (1) available baseline information on sex, age at injury, and year of injury; (2) defined cause of spinal cord injury (e.g., disc herniation, traumatic, ischemic, hemorrhagic); (3) neurological level of injury either “cervical,” “thoracic,” or “lumbar” (i.e., L1 and L2); and (4) neurological assessment of injury severity according to the American Spinal Injury Association (ASIA) Impairment Scale (AIS) [16] (for details see Table 1) at exam stage “very acute” (i.e., <2 weeks post injury) and/or “acute I” (i.e., 2–4 weeks post injury). The neurological level of injury refers to the most caudal segment of the cord with intact sensation and antigravity muscle function strength, provided that there is normal (intact) sensory and motor function rostrally [17]. We excluded patients who had sustained a non-traumatic spinal cord injury (with the exception of ischemic injuries), in whom damage was below the level L2 of the spinal cord, and missing information on injury completeness at the very acute or acute I stage. Ischemic injuries with a determinable disease onset were included owing to the fact that this type of injury is characterized by a sudden disease onset and the rate and pattern of recovery is comparable to traumatic spinal cord injury [18]. The workflow for the individuals included/excluded from our analysis is highlighted in Fig. 1A.

**Table 1:** American Spinal Injury Association (ASIA) Impairment Scale (AIS) describes the functional impairment as a result of spinal cord injury [16]. It consists of five grades ranging from complete loss of function to normal

Grade	Type of injury	Description of injury
A	Sensorimotor complete	No sensory or motor function is preserved in the sacral segments S4-5.
B	Sensory incomplete	Sensory but no motor function is preserved below the neurological level and includes the sacral segments S4-5, AND no motor function is preserved more than three levels below the motor level on either side of the body.
C	Motor incomplete	Motor function is preserved below the neurological level AND more than half of key muscle functions below the neurological level of injury have a muscle grade less than 3.
D	Motor incomplete	Motor incomplete status as defined above, with at least half (half or more) of key muscle functions below the neurological level of injury having a muscle grade ≥ 3.
E	Normal	Normal. If sensation and motor function as tested with the ISNCSCI are graded as normal in all segments, and the patient had prior deficits, then the AIS grade is E. *Someone without an initial spinal cord injury does not receive an AIS grade.*

### Primary outcome (dependent) variables

The primary outcomes were common neurological (total motor score [TMS], lower extremity motor score [LEMS], upper extremity motor score [UEMS], total pinprick score [TPP], total light touch score [TLT], total sensory score [TSS]) and functional outcome scores (Spinal Cord Independence Measure [SCIM], Walking Index for Spinal Cord Injury [WISCI], 10-meter walking test [10MWT], and the 6-minute walk test [6MWT]). For motor scores, key muscles in the upper and lower extremities were examined according to the International Standards for the Neurological Classification of Spinal Cord Injury (ISNCSCI) [17], with a maximum score of 50 points for each, the upper and lower extremities (for a maximum total motor score of 100). Light touch and pin prick (sharp-dull discrimination) scores were also assessed according to ISNCSCI, with a maximum score of 112 each (for a maximum total sensory score of 224) [17]. It is important to note that between 2001 and 2019, different ISNCSCI versions were used to assess the sensorimotor scores. For our analysis, we standardized and recalculated the ISNCSCI data by using the EMSCI ISNCSCI calculator [19] to comply with the 2015 ISNCSCI revision [17]. The SCIM is a scale for the assessment of activities of daily function. Throughout the duration of this study (2001–2019), two different versions of the SCIM were used: between 2001 and 2007 SCIM II [20] and since 2008 SCIM III [21]. The major difference between the versions is that SCIM II does not consider intercultural differences. Both versions contain 19 tasks related to activities of daily living organized in four areas of function (subscales): self-care (scored 0–20); respiration and sphincter management (0–40); mobility in room and toilet (0–10); and mobility indoors and outdoors (0–30). For the longitudinal analysis, we pooled the SCIMII and SCIMIII variables. WISCI has an original scale that quantifies a patient’s dependency on walking aids to travel a distance of 10 m; a score of 0 indicates that a patient cannot stand and walk 10 m and the highest score of 20 is assigned if a patient can walk 10 m without walking aids of assistance [22]. Lastly, 10MWT measures the time (in seconds) it takes a patient to walk 10 m at a self-selected walking speed, and the 6MWT quantifies the distance (in meters) covered by the patient within 6 min [23]. The 10MWT and 6MWT was only analyzed for ambulatory patients.

### Input (independent) variables

Year of injury and exam stage (i.e., time since injury) were selected as the independent variables. Exam stage comprises four levels: very acute (≤2 weeks post injury), acute I (1-month post injury), acute II (3 months post injury), acute III (6 months post injury), and chronic (12 months post injury). The exam stage variable was coded as continuous variable for the estimation of temporal recovery trajectories. As with all observational studies, there is potential for confounding effects and bias. Potential confounders included age, sex, injury completeness (at time of injury) according to the AIS grade [24], and neurological level of injury (cervical, thoracic, or lumbar).

### Data preprocessing and statistical analyses

As part of the preprocessing, we assessed the type and pattern of missing data. Briefly, we tested the hypothesis that the missing data are missing completely at random (MCAR) using the *LittleMCAR* function of the R package *BaylorEdPsych*. To visually explore the pattern of missing data as well as combinations of missingness across cases, we used the R package *naniar*.

In the first step of analysis, descriptive statistics (mean, standard deviations, median, min, max, percentage, and proportions) were used to provide summary information on the demographics, baseline injury characteristics, and baseline functional and neurological outcomes. Independent 2-group Mann-Whitney-*U* and chi-squared tests were used to assess whether there was a difference in demographics and injury characteristics between included and excluded cohorts. Prior to the regression analyses, we normalized and standardized our data (i.e., ExamStage, YEARDOI, AgeAtDOI). Specifically, normalization refers to scaling a variable to have a value between 0 and 1, while standardization transforms data to have a mean of zero and a standard deviation of 1. These two steps are important as they improve the interpretability and computational performance of the described statistical models. Employing linear and logistic regression analysis, we assessed if demographics (i.e., age at injury, ratio of male and female patients) and injury characteristics (i.e., injury severity and neurological level of injury) differed between 2001 and 2019. Variability in injury characteristics were assessed separately for male and female patients. Specifically, the proportions (in percent) of the different injury severities (AIS-A to AIS-D), injury level (cervical, thoracic, and lumbar), and plegia (paraplegia, tetraplegia) were calculated for each year of the surveillance period. Subsequently, we fit a linear regression model with the proportion of AIS-A as the response, and time since injury as the predictor to assess if the confidence interval of the beta coefficient includes zero or not. This was repeated for each AIS grade and all injury levels (i.e., cervical, thoracic, and lumbar). The second step of the analysis entailed the employment of non-linear mixed effect models to address the question if and to what extent the functional and/or neurological recovery were subject to change over the course of the last two decades. We assumed a random intercept and random effect for time since injury [25]. Moreover, we assumed a continuous time autoregressive process of order 1 for within-patient correlation structure and assumed a power function of the mean value for within-patient heteroscedasticity structure [26]. The model was fitted using restricted maximum likelihood for unbiased estimates of variance components. Dependent variables were all primary outcome variables described above, independent variables were year of injury (YEARDOI) and exam stages (ExamStage). To assess time-dependent changes in the recovery trajectories, the independent variables were included as interaction effect (YEARDOI*ExamStage). These analyses were performed for the overall cohort and stratified by sex, plegia, and AIS grades. Confounders of not interest included age and sex. If applicable, we also adjusted for AIS grades. The significance threshold was set at *α*=0.05. Post-hoc pairwise comparisons were Bonferroni corrected to account for multiple comparisons [27]. Lastly, as we expected a covariate-shift in terms of age, we performed a sensitivity analysis to determine if the recovery trajectories of sensorimotor and functional recovery changed in an age-dependent manner throughout the surveillance period. A second sensitivity analysis aimed at testing for sex-specific differences in recovery profiles. The third sensitivity analysis was performed to test the assumption that patients with ischemic and traumatic spinal cord injuries recover in a comparable fashion. For all analyses and figures, R Statistical Software Version 3.5.2 for Mac Os Mojave was used. All analyses were run locally (MacBook Pro, Memory 16GB, Processor 2.3GHz Intel Core i5).

### External validation cohort

In order to externally validate our findings related to the epidemiology as well as neurological recovery trajectories, we analyzed an independent clinical trial dataset [28]. Specially, the Sygen trial was a randomized, prospective, phase III, placebo-controlled, multi-center study testing the efficacy of GM-1 ganglioside therapy in acute, traumatic spinal cord injury. Clinically active from 1992 to 1998, the Sygen trial failed to demonstrate a superior treatment effect of GM-1 over placebo treatment. The Sygen clinical trial enrolled patients with traumatic spinal cord injury who were admitted to trauma centers across the USA and followed them over a year. Detailed information regarding the trial can be found in the Additional file 3. It is noteworthy to mention that the Sygen clinical trial is particularly well-suited to serve as an external validation data set for EMSCI owing to similar granularity in data, timepoints of assessment, duration of follow-up period, and standardized assessments across participating trauma and rehabilitation centers. There is no contemporary dataset that offers comparable data granularity, quality, and depth as the Sygen trial. The workflow for the individuals included/excluded from our analysis is highlighted in Additional file 3: Fig. S1. To maximize the interpretability of cross-data sources comparisons, the same inclusion/exclusion criteria to be included in our analysis as for EMSCI were applied. Similar to the EMSCI data, we standardized and recalculated the ISNCSCI data by using the EMSCI ISNCSCI calculator [19] to comply with the 2015 ISNCSCI revision [17]. The validation was focused on the sensorimotor recovery owing to the comparable assessment methods (i.e., ISNCSCI). In the Sygen trial, functional recovery was assessed with different outcome measures (i.e., Modified Benzel Score, FIM) compared to the EMSCI study making a proper validation of the functional recovery profiles impossible. Lastly, we performed a sensitivity analysis to assess if the recovery trajectories are different for patients who had early surgery (<24h) vs. those with late surgery (>24 h). In light of that, we added the timing of surgery as an independent variable to the models described above.

### Interactive web platform *Neurosurveillance*

In order to enable the spinal cord injury community, researchers, authorities, and policymakers to fully explore the data and results of this study (and beyond), we developed the freely available and open source *Neurosurveillance* web platform. *Neurosurveillance* was implemented with the *Shiny* framework [29], which combines the computational power of the free statistical software R with friendly and interactive web interfaces. Both, the front- and back-end of *Neurosurveillance* have been built using the *shiny dashboard* package [30]. *Neurosurveillance* is available as an online application and is hosted at https://jutzelec.shinyapps.io/neurosurveillance/ and can be accessed via any web browser on any device (e.g., desktop computers, laptops, tablets, smartphones). *Neurosurveillance* is published under the BSD 3-Clause License. The source code of *Neurosurveillance* is available through Github at https://github.com/jutzca/Neurosurveillance/. Further details on the technical implementation can be found in Additional file 4.

### Data sharing and code availability

The data used for this study, including de-identified individual participant data and a data dictionary defining each field or variable within the dataset, can be made available on reasonable request to the corresponding author (CRJ). These data will be made available following publication of this work. Written proposals will be evaluated by the authors, who will render a decision regarding suitability and appropriateness of the use of data. Approval of all authors will be required and a data sharing agreement must be signed before any data are shared. The code to run the analysis as well as create the figures and tables can be found on our Github repository https://github.com/jutzca/SCI_Neurological_Recovery.

### Role of funding source

The funder of the study had no role in study design, data collection, data analysis, data interpretation, or writing of the report. The corresponding author had full access to all the data in the study and had final responsibility for the decision to submit for publication.

## Results

### Cohort summary

Between 2001 and 2019, a total of 5220 individuals were enrolled in the EMSCI (Fig. 1A). Based on our initial inclusion criteria, 4601 patients were eligible for our analysis (mean age at injury, 47.2 ± 19.0 years; 77.0% male); 53.9% were injured at the cervical level, and 51.5% had a motor complete injury at the initial ISNCSCI examination (i.e., AIS-A and AIS-B). Detailed cohort characteristics are provided in Table 2. The average number of patients enrolled per year was 242.2 ± 101.6 (Additional file 2: Fig. S1). As shown in Fig. 1B and summarized in Additional file 2: Table S1, the majority of the patients were admitted to EMSCI centers located in Germany (*n* = 2949, 64.1%), followed by Switzerland (*n* = 451, 9.8%), and the Czech Republic (*n* = 297, 6.5%). Additional file 2: Table S2 provides the demographics and injury characteristics stratified by age groups.

**Table 2:** Demographics and injury characteristics of included EMSCI cohort stratified by sex

	Female (***N***=1059)	Male (***N***=3542)	Overall (***N***=4601)
**Sex**			
Female	1059 (100%)	0 (0%)	1059 (23.0%)
Male	0 (0%)	3542 (100%)	3542 (77.0%)
**Age (years)**			
Mean (SD)	51.1 (20.2)	46.0 (18.4)	47.2 (19.0)
Median [Min, Max]	52.0 [9.00, 94.0]	46.0 [9.00, 92.0]	47.0 [9.00, 94.0]
**Cause**			
Disc herniation	3 (0.3%)	10 (0.3%)	13 (0.3%)
Hemorrhagic	12 (1.1%)	3 (0.1%)	15 (0.3%)
Ischemic	129 (12.2%)	202 (5.7%)	331 (7.2%)
Traumatic	915 (86.4%)	3327 (93.9%)	4242 (92.2%)
**AIS Score**			
A	360 (34.0%)	1459 (41.2%)	1819 (39.5%)
B	136 (12.8%)	418 (11.8%)	554 (12.0%)
C	227 (21.4%)	644 (18.2%)	871 (18.9%)
D	336 (31.7%)	1021 (28.8%)	1357 (29.5%)
**Neurological level of injury**			
Cervical	539 (50.9%)	1899 (53.6%)	2438 (53.0%)
Thoracic	387 (36.5%)	1256 (35.5%)	1643 (35.7%)
Lumbar	133 (12.6%)	387 (10.9%)	520 (11.3%)

American Spinal Injury Association Impairment Scale (AIS): *AIS-A* no sensory or motor function is preserved in the sacral segments S4-5. *AIS-B* sensory but no motor function is preserved below the neurological level and includes the sacral segments S4-5 (LT or PP at S4-5 or DAP), and no motor function is preserved more than three levels below the motor level on either side of the body. *AIS-C* motor function is preserved at the most caudal sacral segments for voluntary anal contraction OR the patient meets the criteria for sensory incomplete status, and has some sparing of motor function more than three levels below the ipsilateral motor level on either side of the body. Less than half of key muscle functions below the single NLI have a muscle grade ≥ 3. *AIS-D* motor incomplete status as defined above, with at least half (half or more) of key muscle functions below the single NLI having a muscle grade ≥ 3. *AIS-E* if sensation and motor function as tested with the ISNCSCI are graded as normal in all segments, and the patient had prior deficits, then the AIS grade is E. Someone without an initial SCI does not receive an AIS grade

A total of 619 EMSCI patients (mean age at injury, 49.7 ± 20.5 years; 77.1% male) were excluded from our analysis (Additional file 2: Table S3). The ratio of male and female patients was comparable between included and excluded cohorts (*X*-squared = 0.006, df = 1, *p*-value = 0.939). However, the cohorts were different in terms of age (*t* = 2.779, df = 697.900, *p*-value = 0.006) and injury characteristics (*X*-squared = 14.106, df = 3, *p*-value = 0.003), with the excluded cohort being older and represented by a larger proportion of AIS-D injuries. For detailed information on the missing data, see Additional file 1: Figs. S1 and S2.

### Epidemiological landscape between 2001 and 2019

The overall ratio between female and male patients remained constant over the last 20 years (*β* = 0.102, standard error = 0.665, *p*-value = 0.880, Fig. 1C). Along these lines, the ratio between female and male patients remained unchanged stratified according to cervical and thoracic/lumbar spine levels (i.e., tetraplegia [ratio 1:3] and paraplegia [ratio 1:3], Additional file 2: Fig. S2A) as well as injury severity (AIS-A [ratio 1:4], B [ratio 1:4], C [ratio 1:3], and D [ratio 1:3], Additional file 2: Fig. S2B). In contrast, the overall distribution of age at injury changed significantly over the years (*β* = 8.603, standard error = 1.045, *p*-value < 0.001). Between 2001 and 2019, there was a shift towards older age at injury (Fig. 1D, Additional file 2: Table S4), which was more prominent in male compared to female patients (interaction effect YEARDOI*Sex_male_: *β* = 5.306, standard error = 2.433, *p* = 0.029, Additional file 2: Fig. S3). This shift in age remained evident after stratifying patients according to their plegia (Additional file 2: Fig. S4A) and injury severity (Additional file 2: Fig. S4B). In terms of the baseline injury severity, the overall proportion (in percentage) of AIS-A, AIS-B, AIS-C, and AIS-D remained constant throughout the study duration (Fig. 1E). The proportions of cervical, thoracic, and lumbar injuries were also unchanged (Fig. 1F). These findings remained constant in post hoc sensitivity analyses of subgroups according to AIS grades (Additional file 2: Fig. S5A) and plegia (Additional file 2: Fig. S5B). When stratified by age groups, linear regression models revealed significant changes in the proportion of injury severities as a function of time (Additional file 2: Fig. S6), with more motor-complete injuries (AIS-A, AIS-B) among female and male patients older than 50 years of age. Summary statistics of all models can be found in the Additional file 2: Table S5.

### Temporal progression of neurological and functional outcomes

**Fig. 2:**
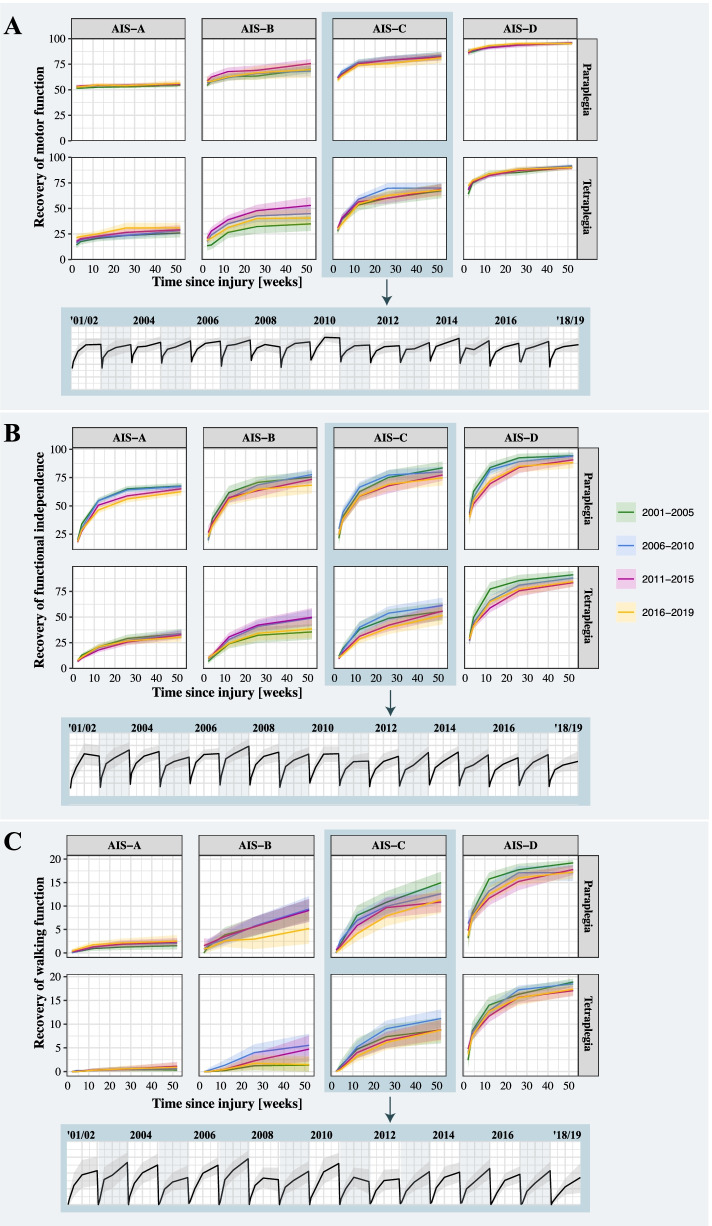
Neurological and functional recovery throughout the surveillance period. The recovery trajectory profiles of **A** the motor function, **B** functional independence, and **C** walking function remained comparable across the surveillance period. In other words, the degree a person with spinal cord injury spontaneously recovers motor and walking function as well as functional independence within 1-year post-injury is the same now as it was two decades ago. The solid lines represent the fitted models and the shaded areas the standard error. The inserted boxes illustrate the robustness of the recovery profiles across all years for patients with AIS-C injuries. For all other injury severities, please refer to the supplementary material section

**Fig. 3:**
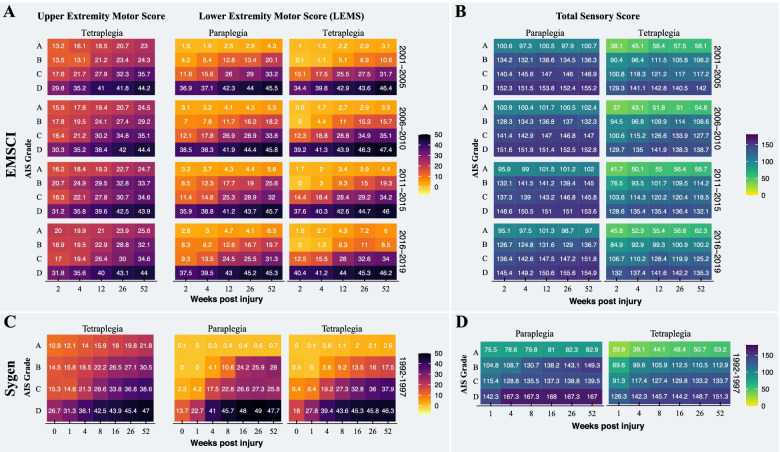
Comparison of sensorimotor recovery between data sources. **A** The pattern and degree recovery of motor and **B** sensory function of patients enrolled in the EMSCI were comparable to those of patients from the Sygen study (**C** and **D**). The heat plots and the number in the tiles represent the mean of motor and sensory scores, respectively. The progression of upper extremity motor scores is only shown for individuals with a tetraplegic spinal cord injury. Note: Individuals with paraplegic spinal cord injury have, by definition, full function in the upper extremities (i.e., UEMS of 50)

The mixed-effect models revealed that recovery trajectories (i.e., fitted regression lines) of all neurological and functional outcomes remained comparable between 2001 and 2019 (Fig. 2). Dependent on the injury severity, the recovery trajectories within a year were characterized by an improvement in function between baseline (i.e., very acute and acute I) and 6 months followed by a plateau phase up to 12 months post injury (Additional file 2: Figs. S7-S10). In addition to the pattern, the rate of sensorimotor recovery remained comparable between the years of the surveillance period (Fig. 3A, B, Additional file 2: Table S6). This was also true when stratifying patients based on sex, plegia, and AIS grades. Summary statistics of all models are provided in Additional file 2: Tables S7-S15. Our sensitivity analyses revealed that the neurological and functional recovery profiles were comparable throughout the surveillance period between different age groups (Additional file 2: Fig. S11 and Table S17), male and female patients (Additional file 2: Fig. S12 and Table S17), and cause of injury (traumatic vs. ischemic, Additional file 2: Fig. S13 and Table S18). The results can be further interactively be explored on our open access and online *Neurosurveillance* platform.

### Validation study

As summarized in Table 3, the validation cohort comprised 703 (mean age at injury, 32.9 ± 13.5 years; 79.7% male, 74.4% motor complete injury). In comparison to EMSCI cohort, the Sygen cohort exhibited a comparable ratio of male and female patients (X-squared = 3.176, df = 1, *p*-value = 0.074). However, the cohorts were different in terms of age (*t* = 2.779, df = 697.900, *p*-value = 0.006) and injury characteristics (AIS grades: *X*-squared = 301.44, df = 3, *p*-value < 0.001, NLI: X-squared = 219.12, df = 2, *p*-value < 0.001), with the Sygen cohort being younger and represented by a larger proportion of AIS-A and cervical injuries.

**Table 3:** Demographics and injury characteristics of Sygen cohort per year and overall

	1992 (*n*=104)	1993 (*n*=161)	1994 (*n*=128)	1995 (*n*=139)	1996 (*n*=159)	1997 (*n*=12)	Overall (*n*=703)
**Sex,*n*(%)**							
Female	23 (22.1)	32 (19.9)	30 (23.4)	24 (17.3)	32 (20.1)	2 (16.7)	143 (20.3)
Male	81 (77.9)	129 (80.1)	98 (76.6)	115 (82.7)	127 (79.9)	10 (83.3)	560 (79.7)
**Age (years)**							
Mean (SD)	33.6 (13.8)	32.0 (13.4)	32.7 (12.9)	32.6 (13.3)	34.2 (14.0)	26.3 (13.2)	32.9 (13.5)
Median [Min, Max]	31.0 [15.0, 69.0]	30.0 [11.0, 66.0]	30.0 [15.0, 69.0]	30.0 [15.0, 67.0]	33.0 [13.0, 69.0]	23.5 [13.0, 60.0]	30.0 [11.0, 69.0]
**AIS Score**^**a**^**,*n*(%)**							
A (complete)	69 (66.3)	102 (63.4)	75 (58.6)	83 (59.7)	106 (66.7)	11 (91.7)	446 (63.4)
B (sensory incomplete)	9 (8.7)	14 (8.7)	16 (12.5)	19 (13.7)	19 (11.9)	0 (0)	77 (11.0)
C (motor incomplete)	22 (21.2)	34 (21.1)	27 (21.1)	34 (24.5)	31 (19.5)	1 (8.3)	149 (21.2)
D (motor incomplete)	4 (3.8)	11 (6.8)	10 (7.8)	3 (2.2)	3 (1.9)	0 (0)	31 (4.4)
**Neurological level of injury,*n*(%)**							
Cervical	81 (77.9)	115 (71.4)	103 (80.5)	112 (80.6)	119 (74.8)	10 (83.3)	540 (76.8)
Thoracic	23 (22.1)	46 (28.6)	25 (19.5)	27 (19.4)	40 (25.2)	2 (16.7)	163 (23.2)

^a^American Spinal Injury Association Impairment Scale (AIS): *AIS-A* no sensory or motor function is preserved in the sacral segments S4-5. *AIS-B* sensory but no motor function is preserved below the neurological level and includes the sacral segments S4-5 (LT or PP at S4-5 or DAP), and no motor function is preserved more than three levels below the motor level on either side of the body. *AIS-C* motor function is preserved at the most caudal sacral segments for voluntary anal contraction OR the patient meets the criteria for sensory incomplete status, and has some sparing of motor function more than three levels below the ipsilateral motor level on either side of the body. Less than half of key muscle functions below the single NLI have a muscle grade ≥ 3. *AIS-D* motor incomplete status as defined above, with at least half (half or more) of key muscle functions below the single NLI having a muscle grade ≥ 3. *AIS-E* if sensation and motor function as tested with the ISNCSCI are graded as normal in all segments, and the patient had prior deficits, then the AIS grade is E. Someone without an initial SCI does not receive an AIS grade

**Fig. 4:**
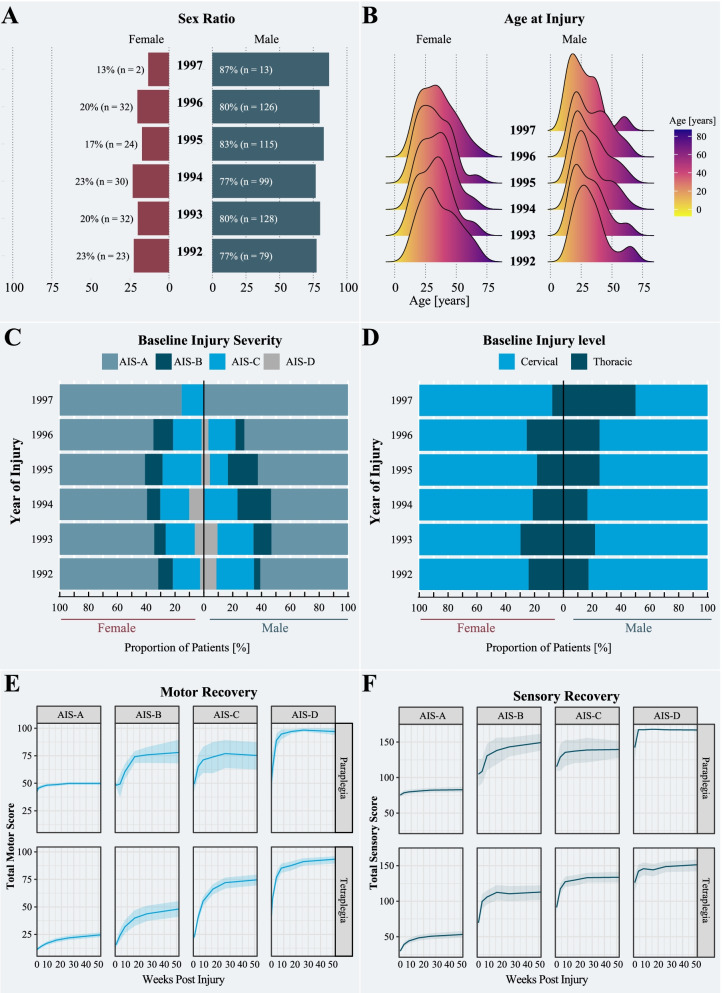
Overview of the validation study. **A** The ratio between male and female individuals with a spinal cord injury. Depending on the year, the ratio of male and female spinal cord injury individuals changed between 3:1 and 4:1. **B** Distribution of age at injury. Throughout the clinical trial period, there was no change in distribution of age at injury. Important to note, the average age at injury of the Sygen clinical trial cohort, independent of sex, was significantly lower compared to the EMSCI cohort. **C** Baseline injury severity and **D** injury level: The proportions of injury characteristics remained constant between 1992 and 1997. **E** Motor and **F** sensory recovery stratified by AIS grade and plegia (i.e., paraplegia or tetraplegia). The solid lines represent the fitted models and the shaded areas the standard error

The ratio of male to female patients remained constant at 3:1 (*β* = 1.247, standard error = 0.668, *p*-value = 0.135; Fig. 4A) and there was no significant change in the distribution of age at injury between 1992 and 1997 (*β* = 0.392, standard error = 1.782, *p*-value = 0.826, Fig. 4B). The proportion (%) of AIS grades remained comparable during the trial period (Fig. 4C) (AIS-A: *β*= 9.833, standard error = 20.484, *p*-value = 0.634; AIS-B: *β*= 2.891, standard error = 3.955, *p*-value = 0.486; AIS-C: *β*= −1.156, standard error = 6.622, *p*-value = 0.865; AIS-D: *β*= −2.148, standard error = 2.707, *p*-value = 0.454). The ratio between patients sustaining cervical and thoracic injuries (*β* = 2.375, standard error = 2.471, *p*-value = 0.454; Fig. 4D) was comparable across the 6 years of study duration. An overview of the excluded cohort (Additional file 2: Table S1) as well as detailed information on the missing data (Additional file 2: Table S2 and Figs. S2 and S3) and model summaries of demographics (Additional file 2: Table S3) can be found in the Additional files 2 and 3. As shown in Fig. 4E and F, the motor and sensory recovery, respectively, were dependent on the injury severity and level (Additional file 2: Fig. 4). The direct comparison with the EMSCI revealed similar pattern and rates of motor (Fig. 3C, Additional file 2: Tables S4 to S6) and sensory recovery (Fig. 3D, Additional file 2: Tables S7 and S8). Age and sex had no effect on the rate and pattern of sensorimotor recovery. Owing to a significant degree of missingness in the functional scores (i.e., Benzel score, >30% data was missing), we refrained from computing functional recovery profiles for the patients enrolled in the Sygen clinical trial. In terms of the surgical timing, there was no statistical difference in the sensorimotor recovery trajectory between the early and late surgery group (Additional file 2: Table S9).

### Interactive web platform *Neurosurveillance*

The *Neurosurveillance* web platform is hosted online and contains three main data visualization parts: (1) epidemiological features, including demographics and injury characteristics (Additional file 4: Fig. S1); (2) functional and neurological profiles (Additional file 4: Fig. S2); and (3) recovery monitoring of single patients or a group thereof. All data from the EMSCI study and the Sygen clinical trial, which was used in this study, can be explored in a customized fashion (e.g., customized selection of patient groups, one time point vs. multiple time-points).

## Discussion

The primary aim of this study was to outline the epidemiological landscape of acute spinal cord injury over the last 20 years, as well as to provide a benchmark for the expected changes in standardized neurological and functional spinal cord injury outcomes. In line with our first hypothesis, the ratio between female and male patients remained fairly stable at approximately 1:3 throughout the surveillance period. The mean age at injury, however, has been steadily increasing over the last 20 years, which is consistent with an aging general population at risk. This increase was accompanied by a shift from a unimodal (i.e., young patients) to a bimodal distribution of age at injury (i.e., young and elderly patients). In terms of injury characteristics, the proportional distribution of injury severities and levels remained stable with the largest percentages of motor complete injuries. Our second hypothesis was not confirmed as neither the rate nor the pattern of neurological and functional recovery has changed since 2001—even after adjusting for injury characteristics and demographics. In essence, the degree a patient with spinal cord injury recovers sensory, motor, and walking function within 1-year post-injury remained stable over the last two decades. With the exception of the change in age at injury, all findings derived from the EMSCI study were confirmed through the external validation analysis of a secondary source of data (i.e., Sygen clinical trial performed in the USA). The similarity of results from these different data sources affirm that our findings are not markedly influenced by temporal or geographical biases or confounding factors related to the study design, timing of data collection, or population structure.

Confirming previous findings, the age at injury progressively increased throughout the surveillance period in both, male and female patients [13, 31, 32]. A shift from an unimodal (i.e., young patients) to a bimodal distribution of age at injury (i.e., young and elderly patients) was observed between 2001 and 2019. A cursory glance at the one of the largest US data sources, Spinal Cord Injury Model Systems (SCIMS) [32], suggests that this upward trend in age at injury is evident since the early 1970s. Possible explanations for this observation are the increasing longevity in the general population along with an increase in propensity for risk taking among the elderly population [33]. Furthermore, the elevated susceptibility for spinal cord injuries among elderly is also attributable to the increasing risk of falling with ageing [34]. In fact, the majority of spinal cord injuries among elderly are sustained traumatically through falls [35]. Comparable to trends in the general population, the changed age structure of the spinal cord injury population has major implications on the medical and nursing services required in the prevention and treatment of spinal cord injury and associated complications [36]. The latter is of particular concern, as the frequency of secondary health complications in older patients with spinal cord injury is markedly higher compared to younger patients [37]. Older age at injury is not only associated with greater number of infections, cardiovascular and metabolic complications, but also more fatigue and a greater risk for cognitive impairments [38]. Moreover, the shift towards bimodal distribution of age at injury also has implications on the design of clinical trials and the stratification of patients as it is imperative that data collected from clinical trials are applicable to the patient population to be treated. Thus, forthcoming clinical trials must ensure an appropriate representation of elderly to provide meaningful and generalizable evidence and knowledge regarding the trialed treatment strategy. A proportionate participation of the elderly individuals in clinical trials is further desirable to allow for statistically meaningful subgroup analyses to account for age-related differences in treatment response (e.g., altered affect pharmacokinetics and pharmacodynamics, adverse drug events due to comorbidities or concomitant drugs).

While the epidemiological landscape has been changing in terms of age, traumatic spinal cord injury remains much more common in men, with incidence rates that are three to four times higher compared to women. Along with reports from the SCIMS [32], the data from the Sygen clinical trial study further corroborate the robustness of the sex ratio. Our findings partially contrast previous reports suggesting an increase in the proportion of female patients since the early 2000s [39]. These divergent observations can be likely be explained by the differences in study size (smaller studies are more prone to outliers), study population (e.g., focus on subgroups vs entire cohort), and duration of observation period (longer time windows allow to account for seasonal fluctuations). Independent of age and sex, the incidence of cervical injures remained higher than that of thoracic/lumbar injuries, as has been reported in other studies [39]. Although not reaching statistical significance, the annual proportion of lower thoracic spine injuries steadily decreased, while a greater number of cervical injuries was consistently recorded over the last two decades. In contrast, no such trend was detected for the injury severities as their distribution remained fairly stable for both male and female patients and independent of the neurological level of injury. Results from the Sygen clinical trial study further suggest that the proportion of sensorimotor complete injuries are following a declining trend since the early 1990s.

Both the rate and pattern of neurological and functional recovery have been extensively studied over the last couple of decades [40, 41]. Generally speaking, recovery after acute spinal cord injury is characterized by an initial period of rapid improvement, with a plateau in sensory and motor function by 1 year, leaving most patients with some permanent neurological and functional deficits [40, 42]. Outcome-modifying factors include injury characteristics (level and severity), age, acute care concepts (early surgical decompression, blood pressure regulation), comorbidities, and medication administered to treat secondary complications (e.g., gabapentionoids) [43, 44]. Our international surveillance study revealed that rate, pattern, and variability of neurological and functional recovery remained stable between 2001 and 2019. As a matter of fact, our validation analysis further suggests that this pattern has been unchanged since the early 1990s and is independent of geographical region, study design (observational vs. controlled clinical trial), and changes in population structure. Independent of the data source and year of injury, changes in neurological score were the greatest for tetra- and paraplegic AIS C patients. A markedly smaller increase was observed for patients with AIS-D injuries owing to ceiling effects of the neurological scores. In contrast, the AIS-C and AIS-D showed the greatest increase in the functional scores, which are less prone to ceiling effects. Our findings are remarkable considering the ongoing changes in the acute care [10, 11, 45] and neurorehabilitation practices aiming at maximizing the functional recovery following a spinal cord injury [46]. However, the mainly applied concept in spinal cord injury rehabilitation still relies on fostering mechanisms of compensation and adaptation, while interventions of true neural repair and induced regeneration have not yet reached clinical practice. It is noteworthy that our study does not allow to make any assumption of the effects of potential changes to the very early acute care (e.g., surgical decompression, specialized transportation from scene of accident to hospital) on recovery. While our study indicates consistent patterns and robust trends for injury characteristics-dependent neurological and functional recovery during early rehabilitation in the sub-acute time period and long-term follow-up of 1 year, it does not capture the immediate effects of very early interventions on the recovery or outcome-modifying factors. Nevertheless, it is noteworthy that our sensitivity analysis revealed that there is yet no significant effect of early surgery on the longitudinal recovery trajectory. This is in line with a recent study by Jaja and colleagues [47] employing group-based longitudinal trajectory modeling. Additionally, the effect of outcome-modifying factors, such as medication, comorbidities, and readmissions, has not been assessed owing to the lack of this data in the EMSCI study. However, given the observed robustness of the recovery patterns, rate, and variability over the years and a fairly large cohort, we carefully conclude that these effects are marginal and might be specific to subpopulations. Future studies, powered to detect effects of outcome-modifying factors, are warranted to investigate the validity of this conclusion.

With recovery rates remaining rather consistent over recent decades, the data from the EMSCI can be pooled across the years making it the largest longitudinal observational study world-wide. EMSCI constitutes an unparalleled resource to inform real-time clinical practice as well as guide the design and implementation forthcoming clinical trials targeting neural repair and neural plasticity [48]. Gauging a patient’s recovery trajectory is challenging owing to the high variability in neurological and functional recovery after injury. Heterogeneous recovery makes accuracy in prognosis at early time-points after injury very difficult and creates a dilemma for clinicians asked to provide a prediction of long-term outcomes to patients and their families. Undoubtedly, there is a great need for accurate and reliable early injury exams or surrogates (e.g., blood biomarkers) thereof. With data from EMSCI, patients that share similar demographics and injury characteristics, physicians can provide a reference context with greater confidence to newly injured patients (i.e., concept of digital twins/siblings) [49]. Having a “digital twin” also allows tracking a patient’s progress, detecting deviations from the projected trajectory, and initiating timely interventions (e.g., treatment of infections) if required. In the context of clinical trials, heterogeneity also adds variability to recovery trajectories, limiting the effectiveness of patient stratification methods, and potentially masking subtle treatment effects. Thus, the provided surveillance data will be instrumental to refine the patient selection and stratification for future clinical trials clinical trials targeting neural repair and neural plasticity.

Beyond this, our study suggests that observational data, such as the EMSCI, could be implemented as historical control data in clinical trials to, at least partially, replace a concurrent control. For rare conditions like acute spinal cord injury, there are a number of distinct advantages to the incorporationof historical control data into clinical trials. Chief among them is increasing the number of participants exposed to treatment and thereby, avoiding early termination of trials owing to difficulties with patient enrollment [50]. Moreover, the incorporation of quality external historical control data (e.g., EMSCI data) allows for reduced mean square error, increased power, and reduced type I error within the current trial [51]. In contrast, should the historical data be inconsistent with current trial control arm data, there is a potential for bias and inflated type I error. Residual confounding cannot just reliably be adjusted away, and misleading (causal and non-causal) associations may not be ruled out. Owing to the standardized data collection and curation by highly trained staff, the EMSCI constitutes a unique source for real-world evidence, particularly for clinical trials that are conducted at EMSCI centers. This is highlighted by the ongoing Nogo Inhibition in Spinal Cord Injury (NISCI) trial (clinicaltrial.gov identifier: NCT03935321). Accumulating evidence suggests that the appropriate usage of real-world evidence can increase the probability of successfully completing a clinical trial and even support regulatory decisions [52].

Our study has limitations. Firstly, the EMSCI database lacks information on mortality, which is an important factor when investigating how modifications to the standard of care change the epidemiological landscape. This limitation is mainly driven by the fact that the majority of the participating centers of EMSCI dedicated comprehensive spinal cord injury care centers to which patients are transferred from trauma centers, where they received acute medical and surgical care. Trauma-related deaths would be recorded in the trauma centers and thus not collected within the EMSCI. Secondly, the standard of care after spinal cord injury (e.g., surgery and timing of surgery, rehabilitation training) was not standardized across the EMSCI centers. Non-uniform standard of care can potentially confound the data and results. In contrast, the Sygen study was completed in a rigorous manner, using a randomized clinical trial protocol designed to limit confounding variables. Despite these differences in study design, the findings related to neurological outcomes were comparable. Thirdly, neither the EMSCI nor the Sygen trial included non-traumatic spinal cord injury, with the exception of ischemic injuries. Longitudinal studies are warranted to shed light on potential changes in epidemiology and recovery profiles of non-traumatic spinal cord injuries. Lastly, EMSCI data have not undergone a thorough monitoring process as typically applied in controlled trials, which is a concern as it might impact the results of the study. Data missingness is inherent to any clinical study and particular observational studies. We addressed this concern by performing a comprehensive examination of the variables and patterns of missing data, which revealed that, in comparison to other observational studies, the degree of missingness is remarkably low.

## Conclusions

In conclusion, the goal of this surveillance study was to provide an unparalleled overview of how the epidemiological landscape of spinal cord injury evolved between 2001 and 2019. Additionally, we addressed the questions whether and to what extent the rate and pattern of neurological and functional recovery changed over the last two decades. Leveraging the largest longitudinal observational spinal cord injury study, we observed a continuation in the previously reported trend toward increasing mean age at injury of new cases, while the ratio between male and female patients as well as the acute injury characteristics remained stable. Most interestingly, the rate and the pattern of neurological and functional recovery did not change throughout the surveillance period. External validation using the data from a landmark clinical trial conducted in the USA corroborated our findings regarding forecastable neurological recovery. It further suggests that our findings are not significantly confounded by geography, study design, and population structure and change thereof. In addition to the longitudinal quantification of the change in the population structure, our study provides a benchmark for expected changes in standardized outcomes after traumatic spinal cord injury. These seminal findings will inform and guide the development and implementation of future clinical trials assessing the safety and effectiveness of novel therapies—with the potential applicability in a multinational setting.

## Supplementary Information

**Additional file 1: Figure S1.** Proportion of missing data in the EMSCI study stratified by the exam stage. **Figure S2.** Proportion of missing data in the EMSCI study stratified by the injury severity (i.e., AIS grade). **Table S1.** Outcome variables and number of observations with missing data (EMSCI).

**Additional file 2: Figure S1.** Annual number of patients enrolled in the EMSCI study. On average 242 patients were included in EMSCI per year across all participating centers. In 2001, EMSCI was founded and only three centers were recruiting patients. **Figure S2.** Ratio of female and male EMSCI patients with traumatic spinal cord injury between 2001 and 2019. (A) The sex ratio remained constant over time in para- and tetraplegic patients. (B) Similarly, no change over time could be observed when stratifying patients according to injury severity, i.e., AIS grades. **Figure S3.** Overall annual distribution of age at injury stratified by sex. Over the last two decades, there was a shift in age at injury for both, male and female individuals with spinal cord injury. In comparison to early 2000’s, which were characterized by a unimodal distribution, the proportion of elderly people sustaining a traumatic spinal cord injury increased significantly. **Figure S4.** Age at injury of EMSCI patients stratified by injury level and severity. Independent of (A) level of injury and (B) injury severity, there was a change in age at injury over the last decade. While in 2001 predominantly young individuals sustained a traumatic spinal cord injury, the proportion of elderly patient significantly increased with time. **Figure S5.** Proportional distribution of (A) injury severity and (B) injury level of EMSCI patients who sustained a traumatic spinal cord injury between 2001 and 2019. The injury severity remained constant over time in the paraplegic and tetraplegic cohort. More pronounced fluctuations were observed in the injury levels across different AIS grades. **Figure S6.** Trend estimates of distribution of injury severity in different age groups for male and female EMSCI patients. Positive estimates indicate in an increase in proportion of a specific AIS grade over timeframe between 2001 and 2019, while negative estimates indicate a decrease. In the age groups below 70 years, the proportion of AIS grades remained constant as opposed to the over 70 years of age group that is characterized by a decrease in severe injuries. In the female population, the heterogeneity in terms of injury severities is greater. This has to be interpreted with caution as the number of female patients is relatively small. **Figure S7.** Sensorimotor recovery between 2001 and 2019. The recovery trajectories of (A) lower and (B) upper extremity, as well as the (C) total motor score, and (D) total sensory score were comparable across the study duration. Less severe injuries (i.e., AIS-C and AIS-D) were associated with a higher sensorimotor recovery. The solid lines represent the fitted models and the shaded areas the standard error. *Note: The total sensory score is computed as the sum of the total pin prick score and total light touch score*. **Figure S8.** Time-series of neurological and functional recovery throughout the surveillance period. The sensorimotor recovery, measured as (A) total motor score, (B) and total sensory score, is characterized by an improvement over the course of one year (i.e., transition from the very acute to chronic phase). (C) Similar pattern and rate of recovery can be observed for the functional outcome, measured by the SCIM2/3. However, neither the pattern nor the degree of neurological and functional recovery changed between 2001 and 2019. In other words, the degree a person with spinal cord injury spontaneously recovers sensory and motor function within one-year post-injury is the same now as it was two decades ago. The solid lines represent the fitted models and the shaded areas the standard error. **Figure S9.** Walking function recovery between 2001 and 2019. The recovery trajectories of the (A) walking endurance, and (B) walking cadence remained comparable throughout the surveillance period. Less severe injuries (i.e., AIS-C and AIS-D) were associated with more functional recovery, including walking. The solid lines represent the fitted models and the shaded areas the standard error. **Figure S10.** Time-series of recovery of walking function throughout the surveillance period. Dependent on the injury severity, walking function, measured by (A) WISCI, (B) 6-minute walking test, and (C) 10m walking test spontaneously recovers, in part, during the transition from the acute to the chronic phase of injury. Importantly, the increase in different aspects of the walking function, such as endurance (6-minute walking test) and cadence (10m walking test), within one-year post-injury remained comparable throughout the surveillance period. The solid lines represent the fitted models and the shaded areas the standard error. **Figure S11.** Sensorimotor recovery trajectories stratified by age-groups and injury characteristics. The (A) motor and (B) sensory recovery remained comparable throughout the surveillance period from 2001 and 2019 and across different age groups. **Figure S12.** Comparison of recovery profiles between female and male patients. The recovery profiles of (A) motor function (i.e., Total motor score), (B) functional independence (i.e., SCIM), and (C) walking function were comparable between patients with traumatic and ischemic spinal cord injuries. **Figure S13.** Comparison of recovery profiles between patients with traumatic and ischemic injuries. The recovery profiles of (A) motor function (i.e., Total motor score), (B) functional independence (i.e., SCIM), and (C) walking function were comparable between patients with traumatic and ischemic spinal cord injuries. **Table S1.** Numbers and proportions of patients enrolled in the EMSCI per country (5-year bins). **Table S2.** Demographics and injury characteristics of included EMSCI cohort stratified by age groups. **Table S3.** Demographics and injury characteristics of excluded EMSCI cohort. **Table S4.** Mean and standard deviation of age at injury for the entire EMSCI cohort between 2001 and 2019 stratified by sex. **Table S5.** Model output of longitudinal analysis of demographics (i.e., sex and age) and baseline injury characteristics (i.e., injury severity and level, plegia). **Table S6.** Overview of longitudinal sensory and motor recovery. Patients enrolled in the EMSCI had 5 follow-up time points, while the patients participating the Sygen trial had seven. Upper extremity motor scores were computed for paraplegic patients only. We report mean (standard deviation); number of patients. **Table S7.** Model output of lower extremity motor score (LEMS) stratified by sex, plegia, and baseline AIS grades. Patients were enrolled in the EMSCI study. Significant values are highlighted in red. **Table S8.** Model output of upper extremity motor score (UEMS) stratified by sex, plegia, and baseline AIS grades. Patients were enrolled in the EMSCI study. Significant values are highlighted in red. *Note: The model was only run for tetraplegic patients*. **Table S9.** Model output of upper extremity motor score (UEMS) stratified by sex, plegia, and baseline AIS grades. Patients were enrolled in the EMSCI study. Significant values are highlighted in red. *Note: The model was only run for tetraplegic patients*. **Table S10.** Model output of total light touch score (TLT) stratified by sex, plegia, and baseline AIS grades. Patients were enrolled in the EMSCI study. Significant values are highlighted in red. **Table S11.** Model output of total pinprick score (TPP) stratified by sex, plegia, and baseline AIS grades. Patients were enrolled in the EMSCI study. Significant values are highlighted in red. **Table S12.** Model output of total sensory score (TSS) stratified by sex, plegia, and baseline AIS grades. Patients were enrolled in the EMSCI study. Significant values are highlighted in red. **Table S13.** Model output of SCIM Total Score stratified by sex, plegia, and baseline AIS grades. Patients were enrolled in the EMSCI study. Significant values are highlighted in red. **Table S14.** Model output of Walking Index for Spinal Cord Injury (WISCI), stratified by sex, plegia, and baseline AIS grades/ Patients were enrolled in the EMSCI study. Significant values are highlighted in red. **Table S15.** Model output of 6-minute walking test (6-MWT) stratified by sex, plegia, and baseline AIS grades. Patients were enrolled in the EMSCI study. Significant values are highlighted in red. *Note: The model was only run for patients with AIS-C and D injuries. Male and female patients were pooled due to low sample numbers*. **Table S16.** Model output of 10m walking test (10-MWT) stratified by sex, plegia, and baseline AIS grades. Patients were enrolled in the EMSCI study. Significant values are highlighted in red. *Note: The model was only run for patients with AIS-C and D injuries*. **Table S17.** Sensitivity Analysis II: Sex. Model output of total motor score, SCIM2 and 3, and WISCI stratified by plegia, and baseline AIS grades. Patients were enrolled in the EMSCI study. Significant values are highlighted in red. **Table S18.** Sensitivity Analysis III: Cause of Injury. Model output of total motor score, SCIM2 and 3, and WISCI stratified by plegia, and baseline AIS grades. Patients were enrolled in the EMSCI study. Significant values are highlighted in red.

**Additional file 3: Figure S1.** Flow chart of included and excluded subjects. **Figure S2.** Proportion of missing data in the Sygen trial stratified by the exam stage. **Figure S3.** Proportion of missing data in the Sygen trial stratified by the injury severity (i.e., AIS grade). **Figure S4.** Sensorimotor recovery after spinal cord injury in patients enrolled in the Sygen clinical trial. Recovery of (A) lower and (B) upper extremity motor score as well as (C) total light touch and (D) total pin prick are all dependent on the injury level (tetra- vs. paraplegia) and the injury severity (i.e., AIS grades). **Table S1.** Details on excluded Sygen cohort. **Table S2.** Outcome variables and number of observations with missing data (Sygen). **Table S3.** Model output of longitudinal analysis of demographics (i.e., sex and age) and baseline injury characteristics (i.e., injury severity and level, plegia). **Table S4.** Model output of lower extremity motor score (LEMS) stratified by sex, plegia, and AIS grades. Patients were enrolled in the Sygen Trial. Note: In case the number of patients per group were too low (*n* < 3), the model did not converge and thus, the results are not represented in the table. **Table S5.** Model output of upper extremity motor score (UEMS) stratified by sex and AIS grades. Patients were enrolled in the Sygen Trial. Note: This analysis was only conducted for the tetraplegic patients. **Table S7.** Model output of total light touch (TLT) stratified by sex, plegia, and AIS grades. Patients were enrolled in the Sygen Trial. Note: In case the number of patients per group were too low (*n* < 3), the model did not converge and thus, the results are not represented in the table. **Table S8.** Model output of total pinprick (TPP) stratified by sex, plegia, and AIS grades. Patients were enrolled in the Sygen Trial. Note: In case the number of patients per group were too low (*n* < 3), the model did not converge and thus, the results are not represented in the table. **Table S9.** Sensitivity analysis: Surgical timing on total motor score stratified by AIS grades. Patients were enrolled in the Sygen Trial. Note: In case the number of patients per group were too low (*n* < 3), the model did not converge and thus, the results are not represented in the table.

**Additional file 4: Figure S1.** Epidemiological tab in Neurosurveillance. The user can interactively explore the changes in the epidemiological landscape in the EMSCI and Sygen data sets. The platform allows the user to customize the selection of patients to visualize. **Figure S2.** Epidemiological tab in Neurosurveillance. The user can interactively explore the changes in the epidemiological landscape in the EMSCI and Sygen data sets. The platform allows the user to customize the selection of patients to visualize.

## Data Availability

The data used for this study, including de-identified individual participant data and a data dictionary defining each field or variable within the dataset, can be made available on reasonable request to the corresponding author (CRJ). These data will be made available following publication of this work. Written proposals will be evaluated by the authors, who will render a decision regarding suitability and appropriateness of the use of data. Approval of all authors will be required and a data sharing agreement must be signed before any data are shared. The code to run the analysis as well as create the figures and tables can be found on our Github repository (https://github.com/jutzca/SCI_Neurological_Recovery).
