# Supplementary material for: International surveillance study in acute spinal cord injury confirms viability of multinational clinical trials
Source: BMC Med. 2022 Jun 14;20:225. doi: 10.1186/s12916-022-02395-0 (PMC9202190; doi:10.1186/s12916-022-02395-0)
Supplement: Supplementary file 1 — Additional file 1: Figure S1. Proportion of missing data in the EMSCI study stratified by the exam stage. Figure S2. Proportion of missing data in the EMSCI study stratified by the injury severity (i.e., AIS grade). Table S1. Outcome variables and number of observations with missing data (EMSCI). [file 12916_2022_2395_MOESM1_ESM.pdf]

Additional File 1

1. Further Details on Data Source: EMSCI

Governance, study design

Founded in 2001 and lead by the University Clinic Balgrist (Zurich, Switzerland), the EMSCI network comprises of 22 participating trauma and rehabilitation centers from across Europe (Austria, Czech Republic, France, Germany, Great Britain, Italy, Netherlands, Spain, Switzerland) and India (https://www.emsci.org/index.php/members). Collectively, the EMSCI network has collected data from more than 5000 individuals with spinal cord injury. The EMSCI is an ongoing longitudinal, observational study that prospectively collects clinical, functional, and neurophysiological data at fixed time points over the first year of injury: very acute (within 2 weeks), acute I (1 month), acute II (3 months), and acute III (6 months), and chronic (12 months).

Inclusion and exclusion criteria

Three inclusion criteria have to be met before a patient can be enrolled in the EMSCI: (1) the patient has to be capable and willing to give written informed consent; (2) the injury was caused by a single event; and (3) the first EMSCI assessment has to be possible within the first 6 weeks following injury. Patients are excluded from EMSCI for the following reasons: (1) nontraumatic para- or tetraplegia (e.g., discus prolaps, tumor, AV-malformation, myelitis) excl. single event ischemic incidences; (2) previously known dementia or severe reduction of intelligence, leading to reduced capabilities of cooperation or giving consent; (3) peripheral nerve lesions above the level of lesion (e.g., plexus brachialis impairment); (4) pre-existing polyneuropathy; and (5) severe craniocerebral injury. All individuals in the EMSCI receive standards of rehabilitation care.

Quality management

Since August 2010, EMSCI has been ISO 9001:2015-certified. To maintain a high level of quality, the EMSCI network offers instructional ISNCSCI courses to their members. The training is instructed by experienced ISNCSCI examiners and raters. A recent study showed that ISNCSCI training significantly improves the classification skills regardless of the experience in spinal cord injury medicine.

Certificate: https://www.emsci.org/images/docs/HD_alle_Zentren_EN_38769ms_en_00.pdf

2. Analysis of Missing Data in the EMSCI

Our analysis revealed around 6.4% missing data and 12 variables that were affected. The t-test of mean difference revealed that the missing data is not Missing Completely at Random (MCAR), but rather missing at random (MAR) (Chi-square = 11941·91; df = 973; $p < 0.001$). This suggests that the missing values are not randomly distributed across observations, but are distributed within one or more sub-samples. Demographics and injury characteristics have been documented with a marginal number of missing data, while neurological, functional, and clinical assessments were most vulnerable to missing values. Variables and number of observations with missing are listed in Table A1.1. Note, multiple observations per patients were recorded and thus, can be missing. Assessments of walking function were prone to missing data as a consequence of the fact that it is often only assessed in patients with minimal or lack walking ability. A closer inspection of the missing data revealed that there were significant differences of proportion of missing data depending on the exam stage (Figure A1.1) and injury severity (Figure A1.2). That is, the least missing values were recorded at acute I and acute II, while a large proportion of data was missing at the very acute, acute III, and chronic stages. In terms of injury severity, patients with AIS-C and AIS-D injuries recorded the highest proportions of missing data.

Additional File 1: Table S1. Outcome variables and number of observations with missing data (EMSCI)

Variable	Count (n)	Proportion [%]
Upper extremity motor score	3	<0·1
Lower extremity motor score	4	<0·1
Total motor score	3	<0·1
Voluntary anal contraction	327	2·1
Deep anal pressure	319	2·1
Combined Total Score SCIM2 and 3	1131	7·4
Walking Index for Spinal Cord Injury	900	5·9
6min walking test	1598	10·4
10m walking test	1246	8·1

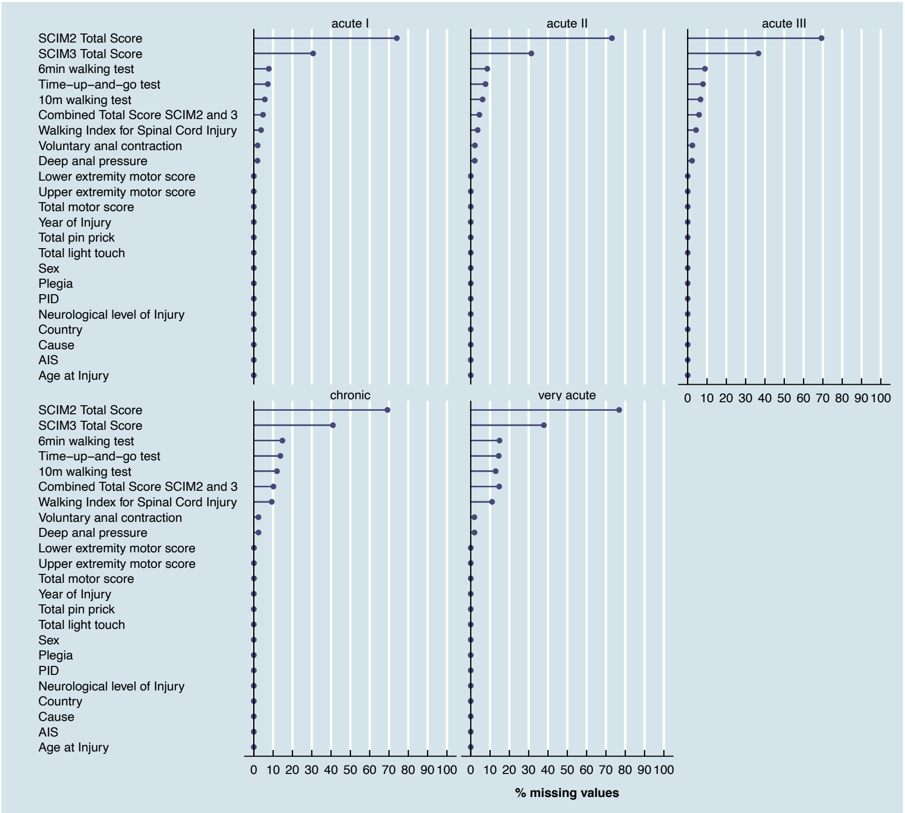

Additional File: Figure S1. Proportion of missing data in the EMSCI study stratified by the exam stage.

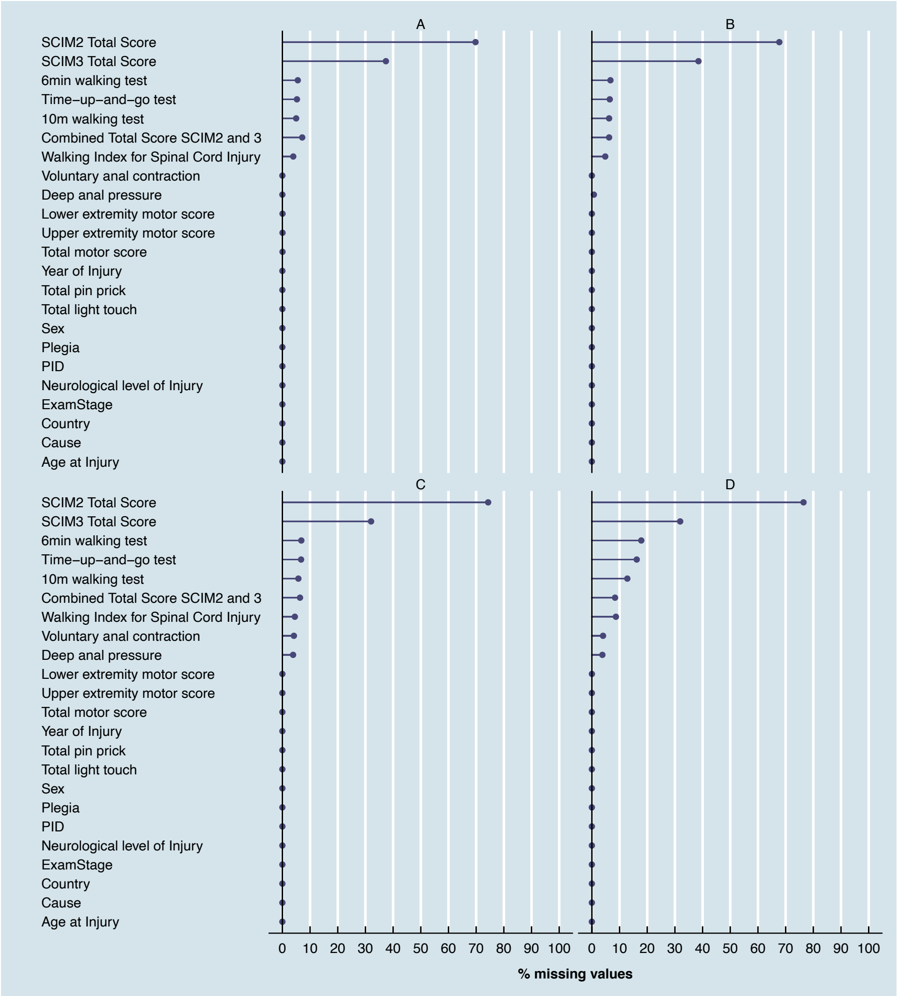

Additional File 1: Figure S2. Proportion of missing data in the EMSCI study stratified by the injury severity (i.e., AIS grade).
