# Supplementary material for: International surveillance study in acute spinal cord injury confirms viability of multinational clinical trials
Source: BMC Med. 2022 Jun 14;20:225. doi: 10.1186/s12916-022-02395-0 (PMC9202190; doi:10.1186/s12916-022-02395-0)
Supplement: Supplementary file 2 — Additional file 2: Figure S1. Annual number of patients enrolled in the EMSCI study. On average 242 patients were included in EMSCI per year across all participating centers. In 2001, EMSCI was founded and only three centers were recruiting patients. Figure S2. Ratio of female and male EMSCI patients with traumatic spinal cord injury between 2001 and 2019. (A) The sex ratio remained constant over time in para- and tetraplegic patients. (B) Similarly, no change over time could be observed when stratifying patients according to injury severity, i.e., AIS grades. Figure S3. Overall annual distribution of age at injury stratified by sex. Over the last two decades, there was a shift in age at injury for both, male and female individuals with spinal cord injury. In comparison to early 2000’s, which were characterized by a unimodal distribution, the proportion of elderly people sustaining a traumatic spinal cord injury increased significantly. Figure S4. Age at injury of EMSCI patients stratified by injury level and severity. Independent of (A) level of injury and (B) injury severity, there was a change in age at injury over the last decade. While in 2001 predominantly young individuals sustained a traumatic spinal cord injury, the proportion of elderly patient significantly increased with time. Figure S5. Proportional distribution of (A) injury severity and (B) injury level of EMSCI patients who sustained a traumatic spinal cord injury between 2001 and 2019. The injury severity remained constant over time in the paraplegic and tetraplegic cohort. More pronounced fluctuations were observed in the injury levels across different AIS grades. Figure S6. Trend estimates of distribution of injury severity in different age groups for male and female EMSCI patients. Positive estimates indicate in an increase in proportion of a specific AIS grade over timeframe between 2001 and 2019, while negative estimates indicate a decrease. In the age groups below 70 years, the proportion of A [file 12916_2022_2395_MOESM2_ESM.pdf]

Additional File 2

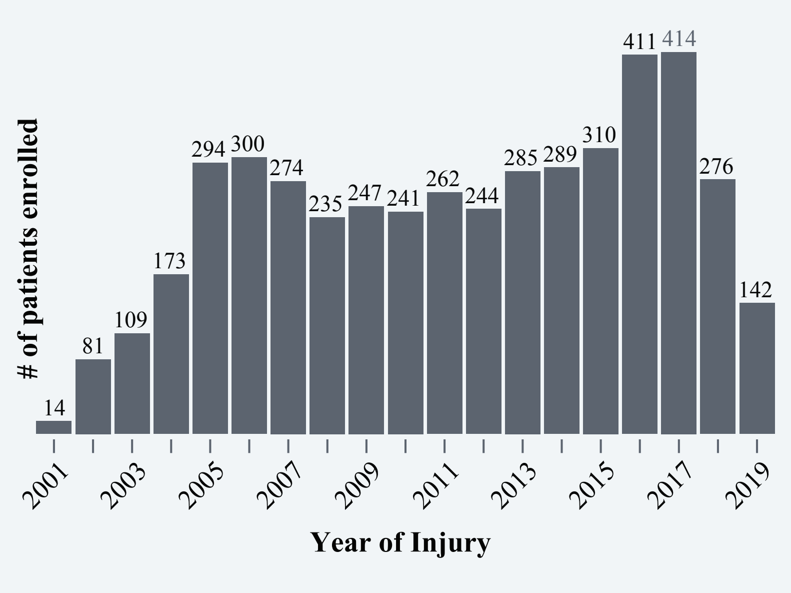

Additional File 2: Figure S1. Annual number of patients enrolled in the EMSCI study. On average 242 patients were included in EMSCI per year across all participating centers. In 2001, EMSCI was founded and only three centers were recruiting patients.

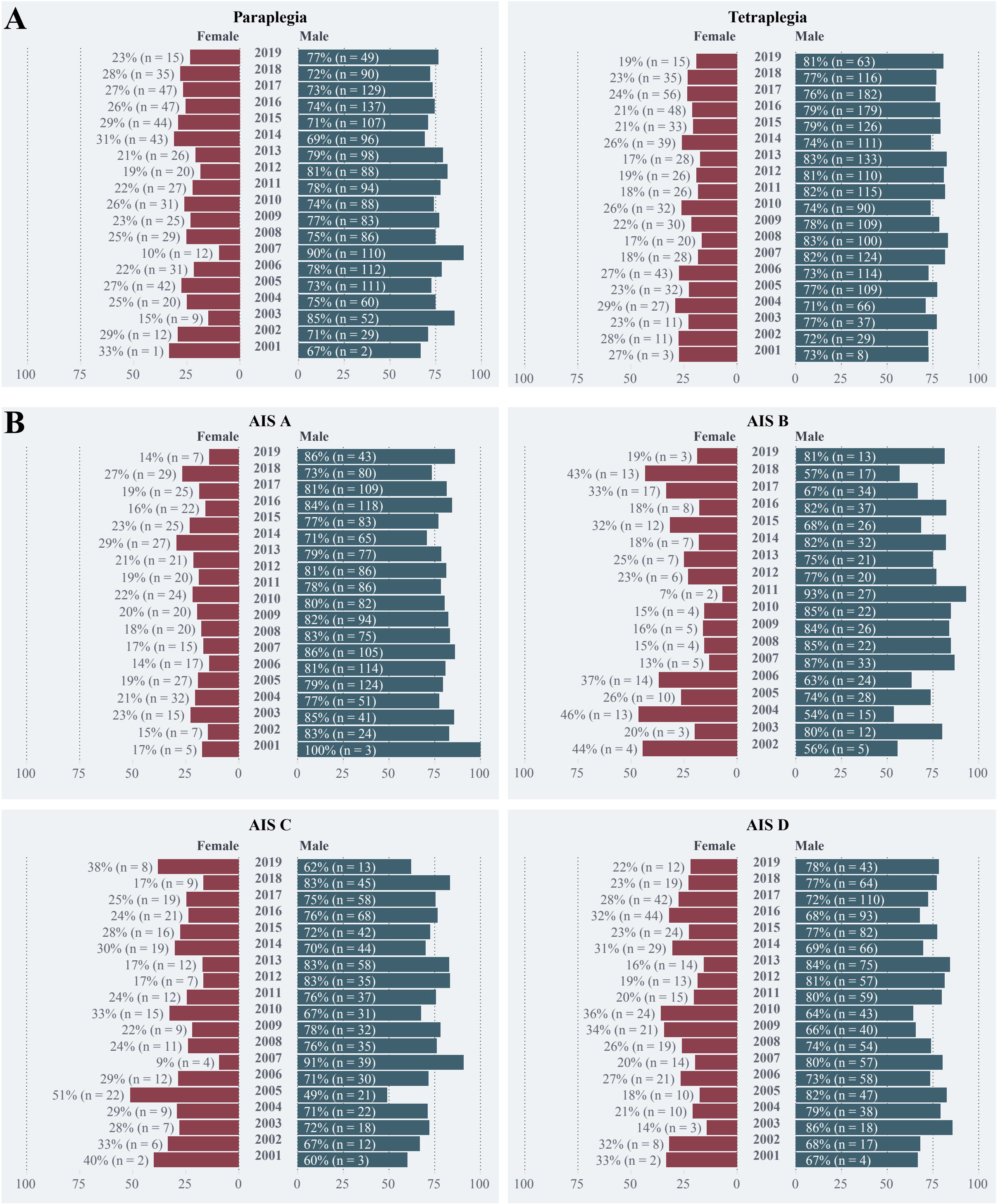

Additional File 2: Figure S2. Ratio of female and male EMSCI patients with traumatic spinal cord injury between 2001 and 2019. **(A)** The sex ratio remained constant over time in para- and tetraplegic patients. **(B)** Similarly, no change over time could be observed when stratifying patients according to injury severity, i.e., AIS grades.

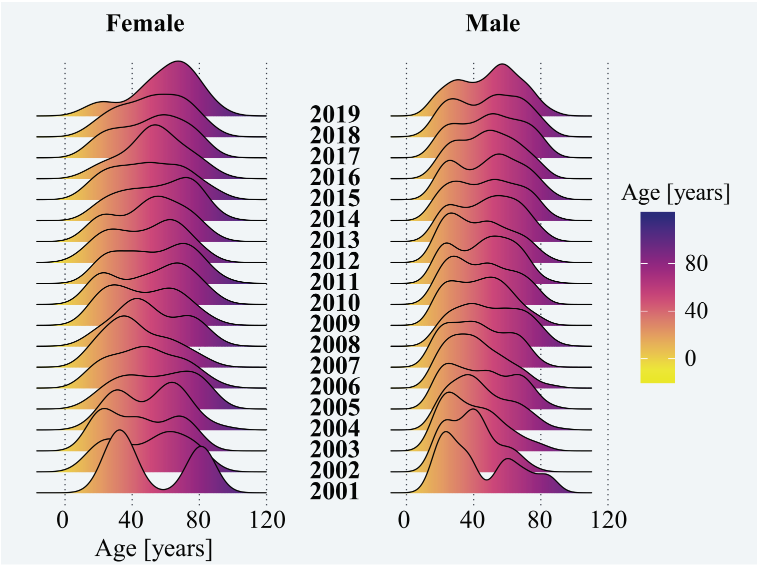

Additional File 2: Figure S3. Overall annual distribution of age at injury stratified by sex. Over the last two decades, there was a shift in age at injury for both, male and female individuals with spinal cord injury. In comparison to early 2000's, which were characterized by a unimodal distribution, the proportion of elderly people sustaining a traumatic spinal cord injury increased significantly.

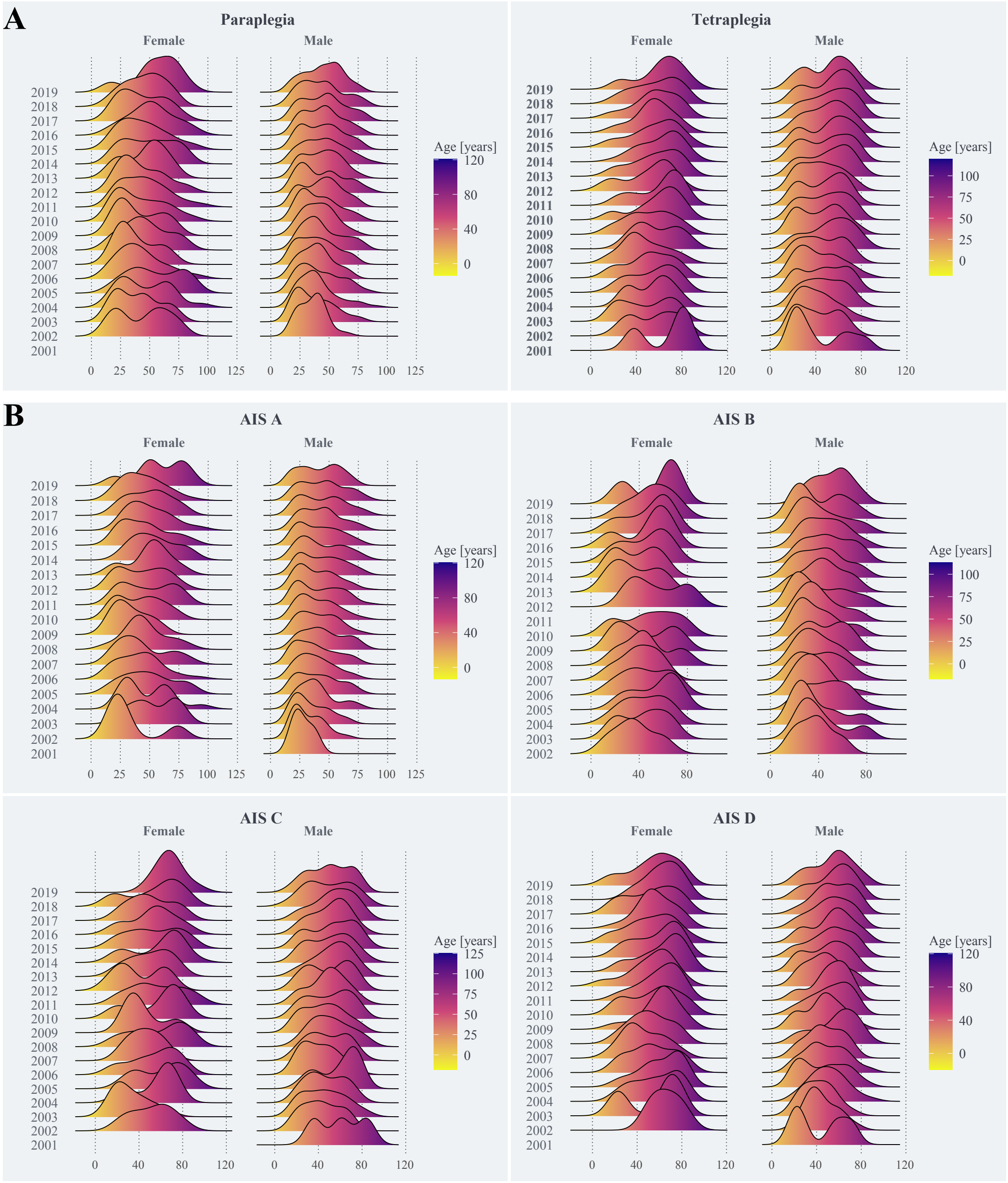

Additional File 2: Figure S4. Age at injury of EMSCI patients stratified by injury level and severity. Independent of (A) level of injury and (B) injury severity, there was a change in age at injury over the last decade. While in 2001 predominantly young individuals sustained a traumatic spinal cord injury, the proportion of elderly patient significantly increased with time.

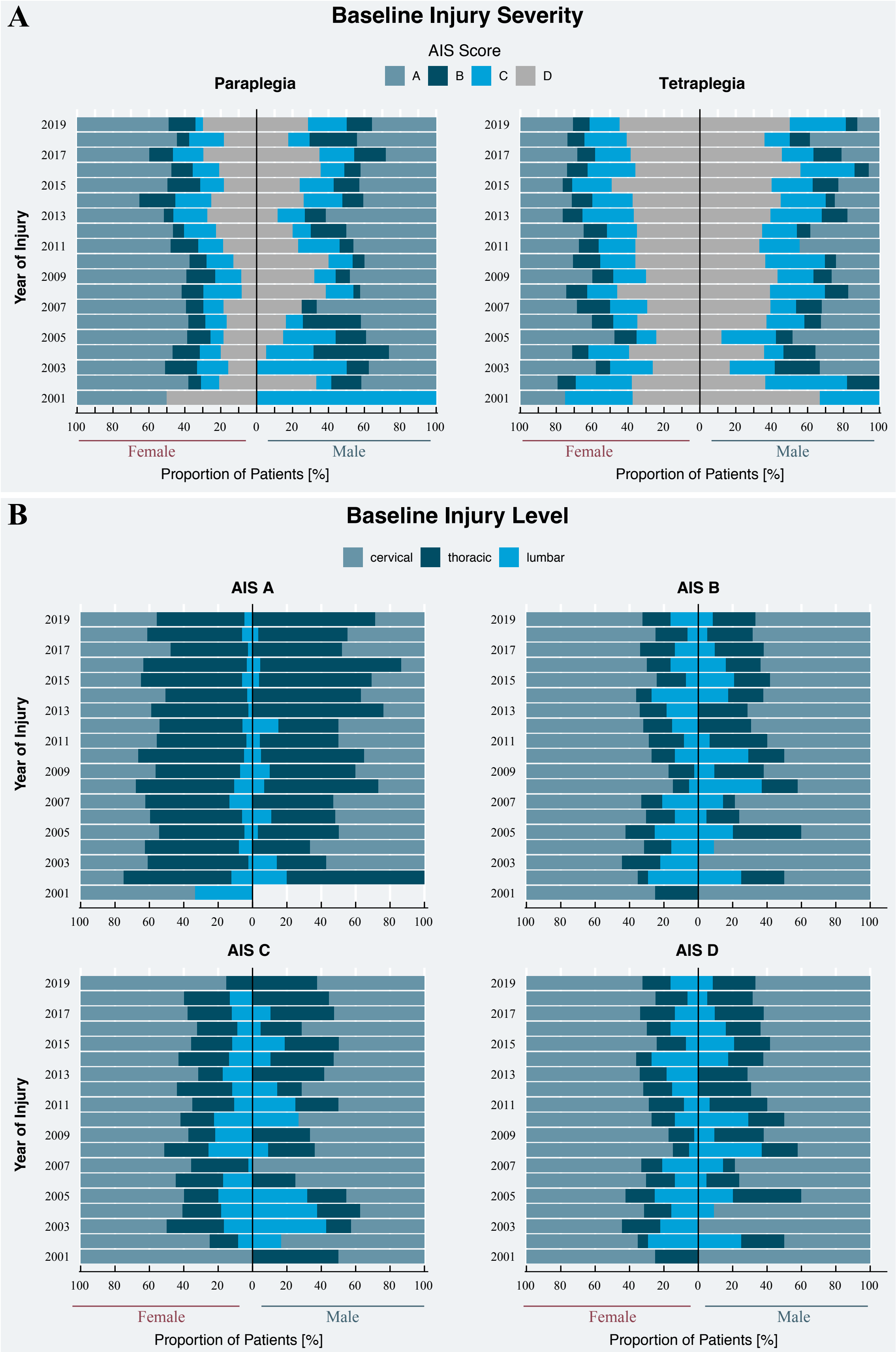

Additional File 2: Figure S5. Proportional distribution of (A) injury severity and (B) injury level of EMSCI patients who sustained a traumatic spinal cord injury between 2001 and 2019. The injury severity remained constant over time in the paraplegic and tetraplegic cohort. More pronounced fluctuations were observed in the injury levels across different AIS grades.

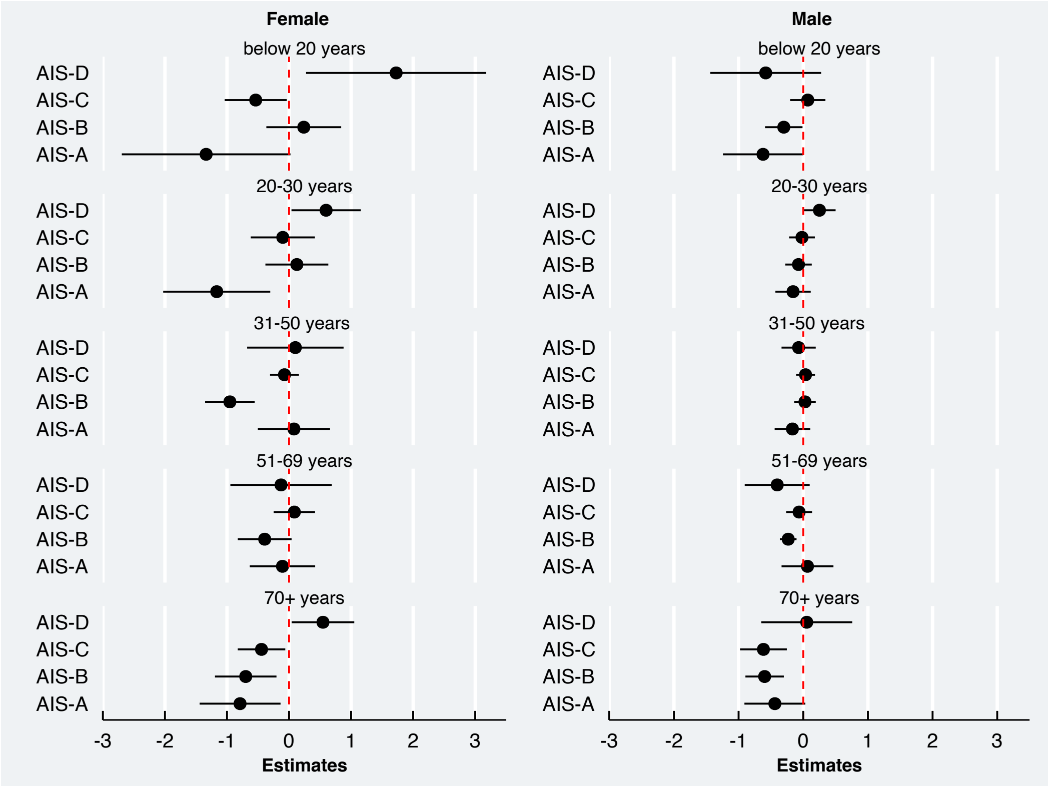

Additional File 2: Figure S6. Trend estimates of distribution of injury severity in different age groups for male and female EMSCI patients. Positive estimates indicate in an increase in proportion of a specific AIS grade over timeframe between 2001 and 2019, while negative estimates indicate a decrease. In the age groups below 70 years, the proportion of AIS grades remained constant as opposed to the over 70 years of age group that is characterized by a decrease in severe injuries. In the female population, the heterogeneity in terms of injury severities is greater. This has to be interpreted with caution as the number of female patients is relatively small.

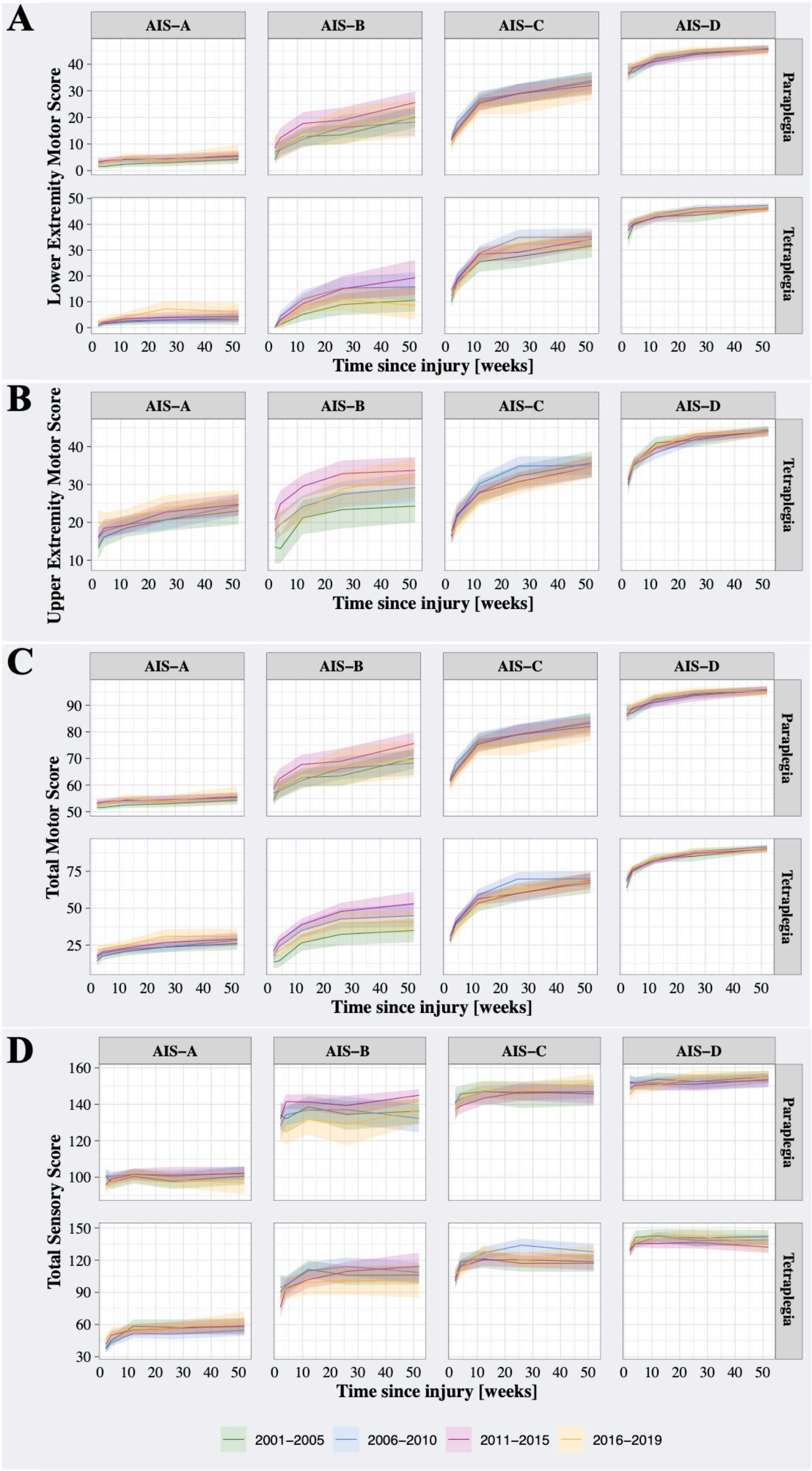

Additional File 2: Figure S7. Sensorimotor recovery between 2001 and 2019. The recovery trajectories of **(A)** lower and **(B)** upper extremity, as well as the **(C)** total motor score, and **(D)** total sensory score were comparable across the study duration. Less severe injuries (i.e., AIS-C and AIS-D) were associated with a higher sensorimotor recovery. The solid lines represent the fitted models and the shaded areas the standard error. *Note: The total sensory score is computed as the sum of the total pin prick score and total light touch score.*

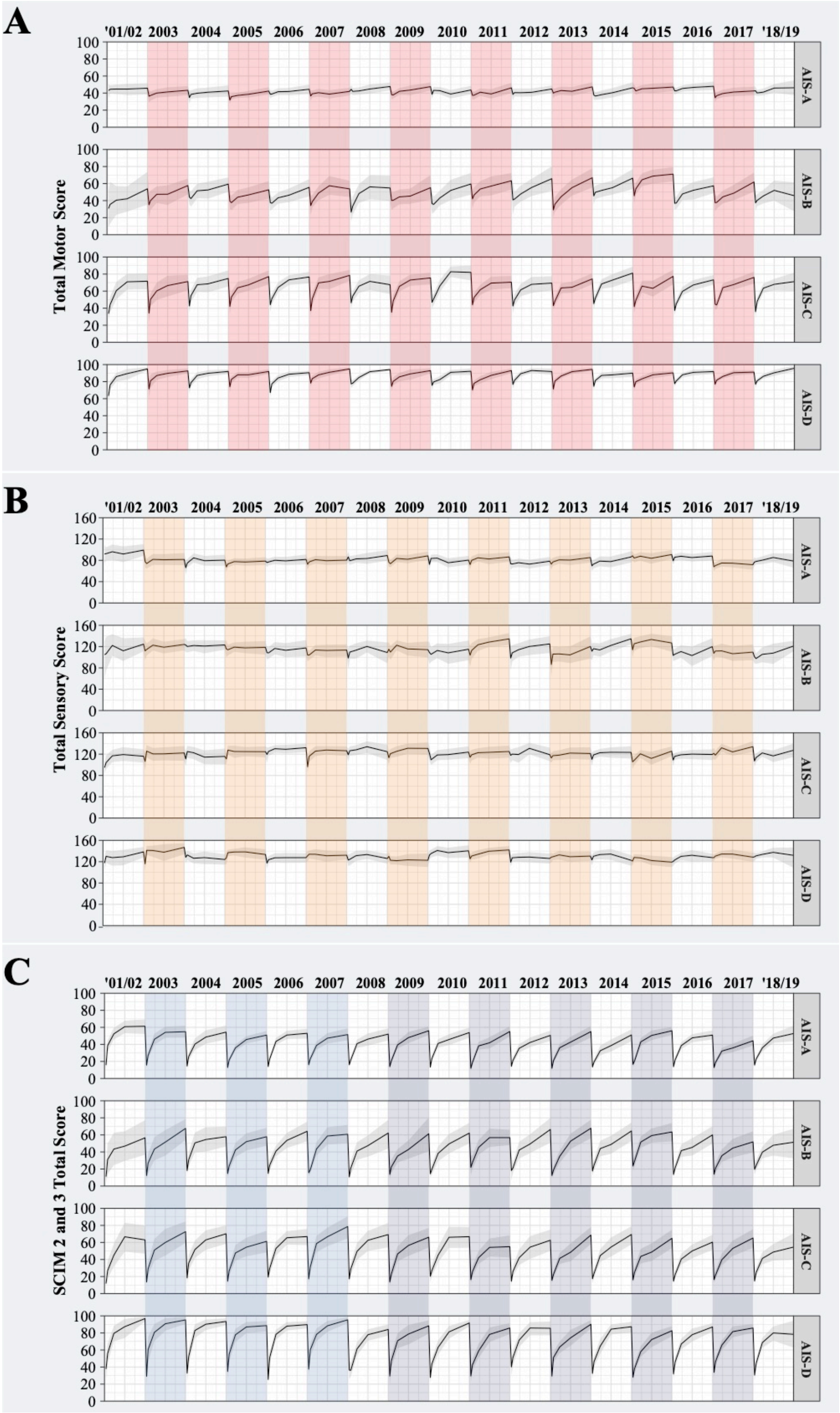

Additional File 2: Figure S8. Time-series of neurological and functional recovery throughout the surveillance period. The sensorimotor recovery, measured as **(A)** total motor score, **(B)** and total sensory score, is characterized by an improvement over the course of one year (i.e., transition from the very acute to chronic phase). **(C)** Similar pattern and rate of recovery can be observed for the functional outcome, measured by the SCIM2/3. However, neither the pattern nor the degree of neurological and functional recovery changed between 2001 and 2019. In other words, the degree a person with spinal cord injury spontaneously recovers sensory and motor function within one-year post-injury is the same now as it was two decades ago. The solid lines represent the fitted models and the shaded areas the standard error.

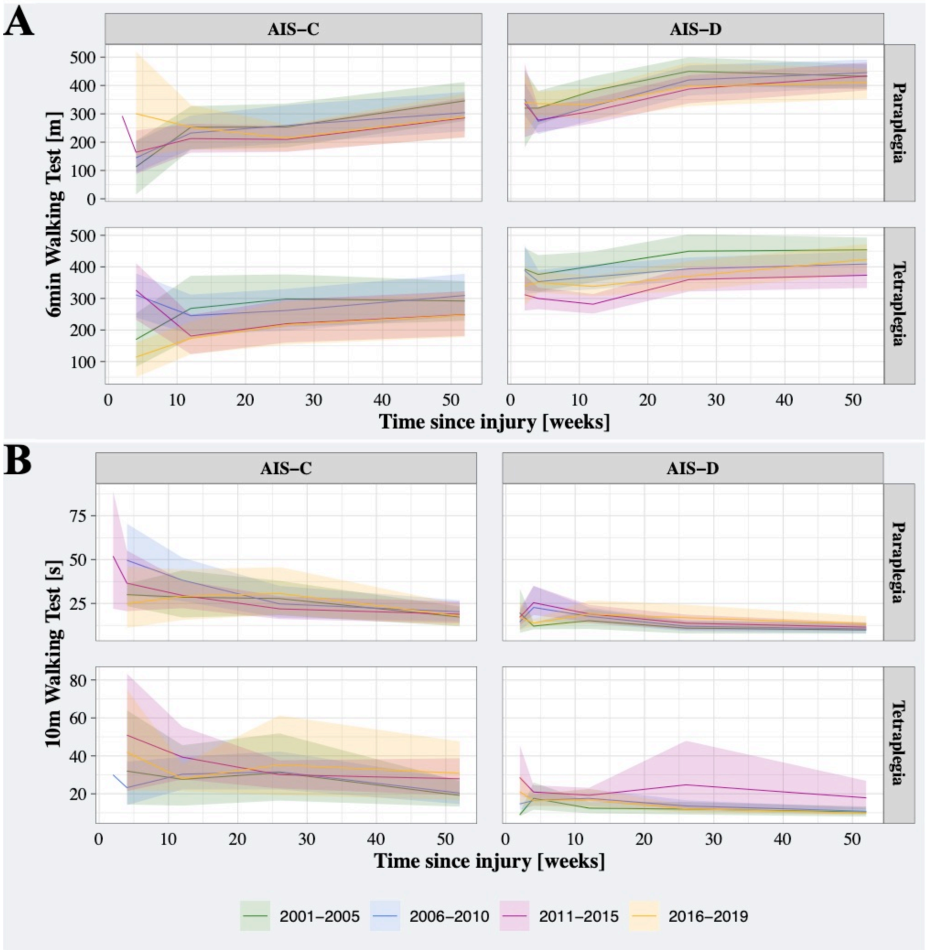

Additional File 2: Figure S9. Walking function recovery between 2001 and 2019. The recovery trajectories of the **(A)** walking endurance, and **(B)** walking cadence remained comparable throughout the surveillance period. Less severe injuries (i.e., AIS-C and AIS-D) were associated with more functional recovery, including walking. The solid lines represent the fitted models and the shaded areas the standard error.

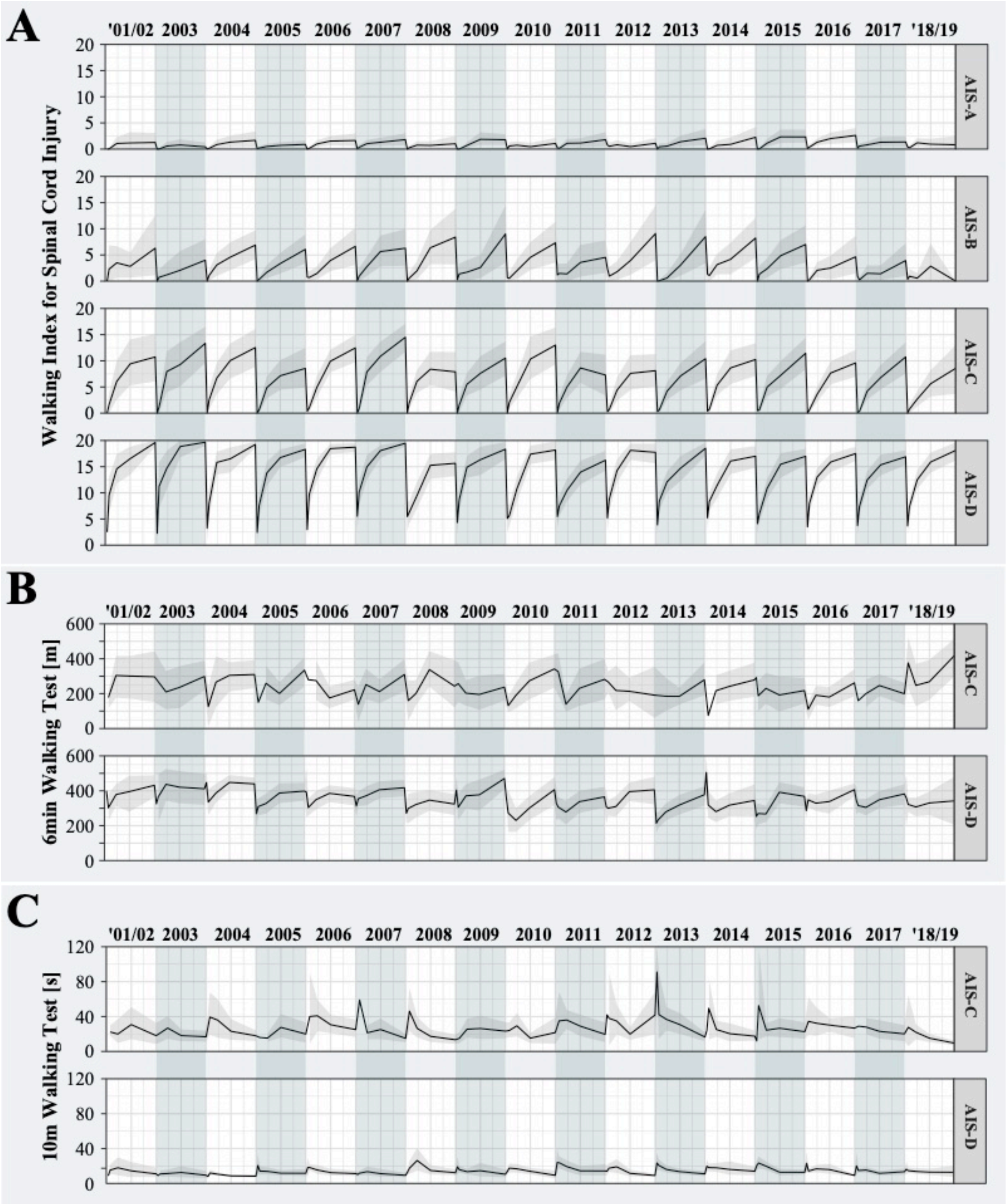

Additional File 2: Figure S10. Time-series of recovery of walking function throughout the surveillance period. Dependent on the injury severity, walking function, measured by **(A)** WISCI, **(B)** 6-minute walking test, and **(C)** 10m walking test spontaneously recovers, in part, during the transition from the acute to the chronic phase of injury. Importantly, the increase in different aspects of the walking function, such as endurance (6-minute walking test) and cadence (10m walking test), within one-year post-injury remained comparable throughout the surveillance period. The solid lines represent the fitted models and the shaded areas the standard error.

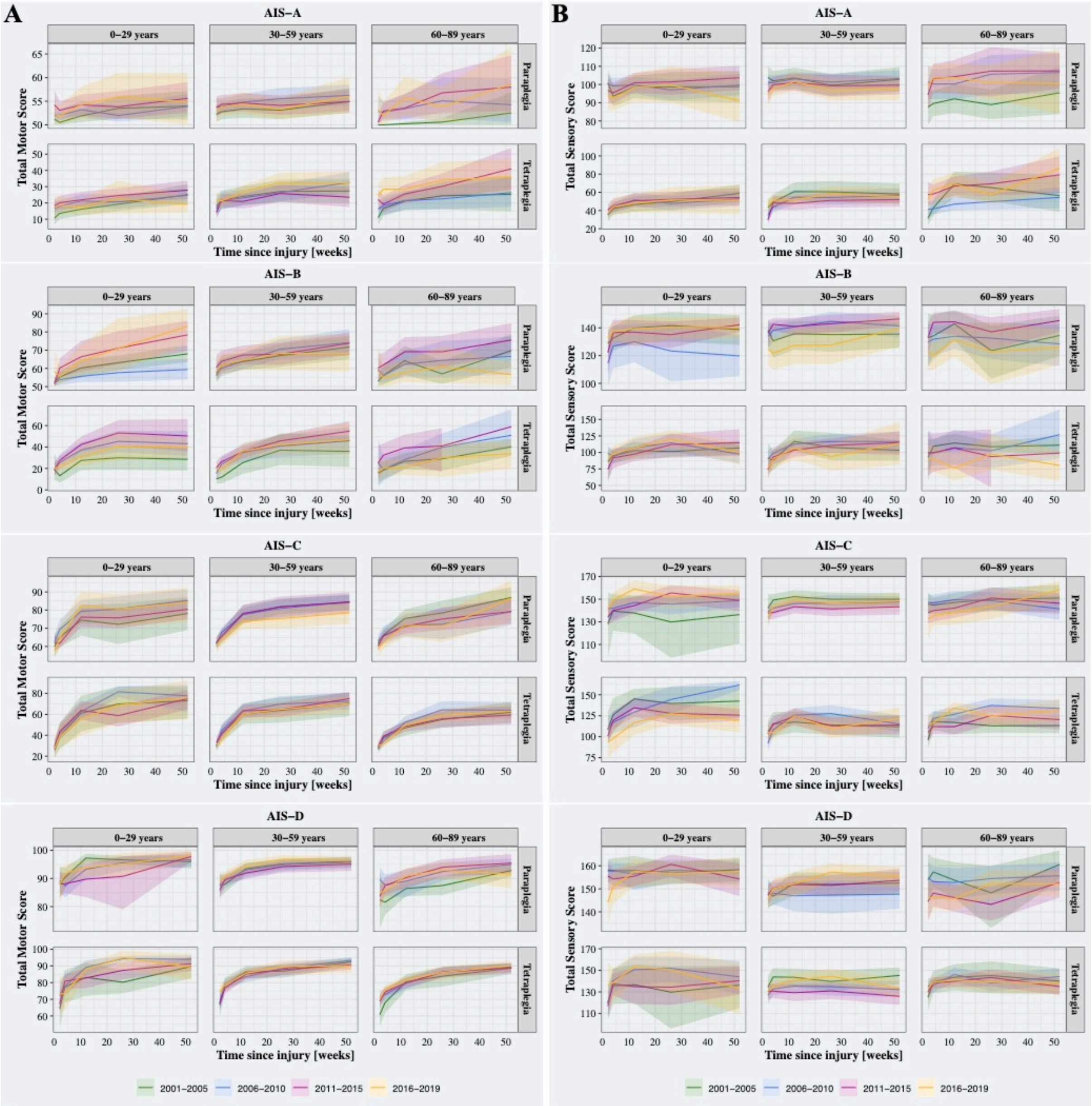

Additional File 2: Figure S11. Sensorimotor recovery trajectories stratified by age-groups and injury characteristics. The **(A)** motor and **(B)** sensory recovery remained comparable throughout the surveillance period from 2001 and 2019 and across different age groups.

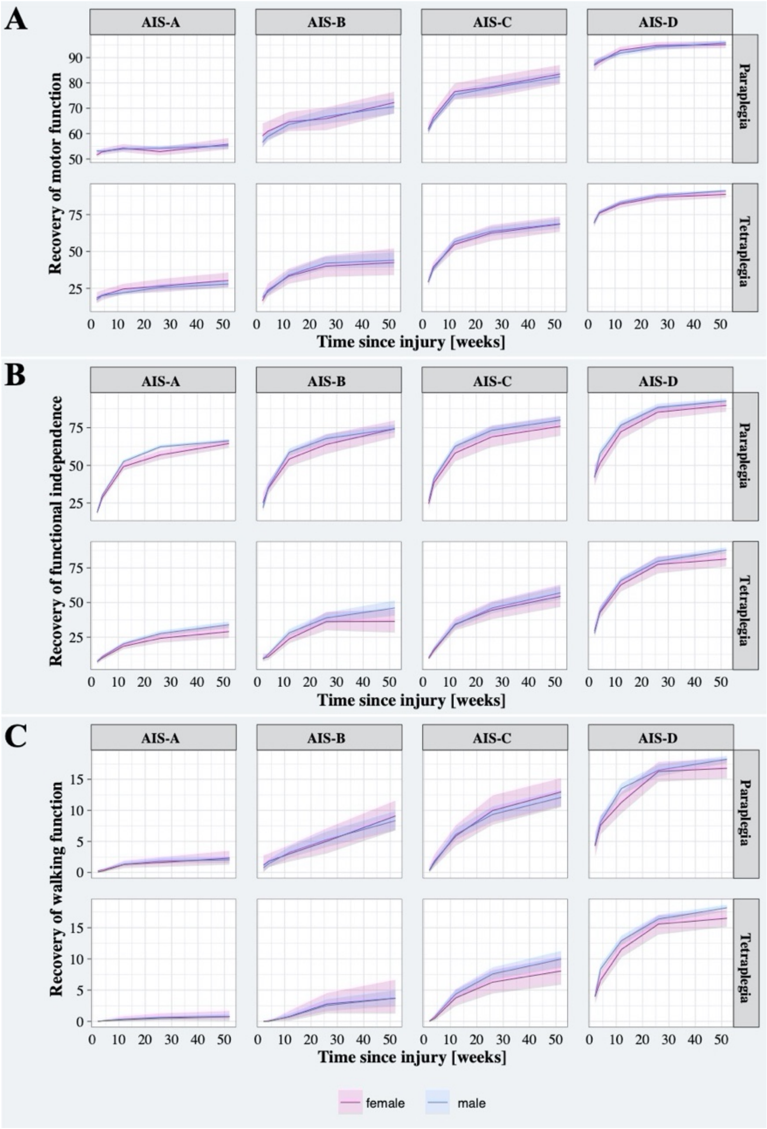

Additional File 2: Figure S12. Comparison of recovery profiles between female and male patients. The recovery profiles of **(A)** motor function (i.e., Total motor score), **(B)** functional independence (i.e., SCIM), and **(C)** walking function were comparable between patients with traumatic and ischemic spinal cord injuries.

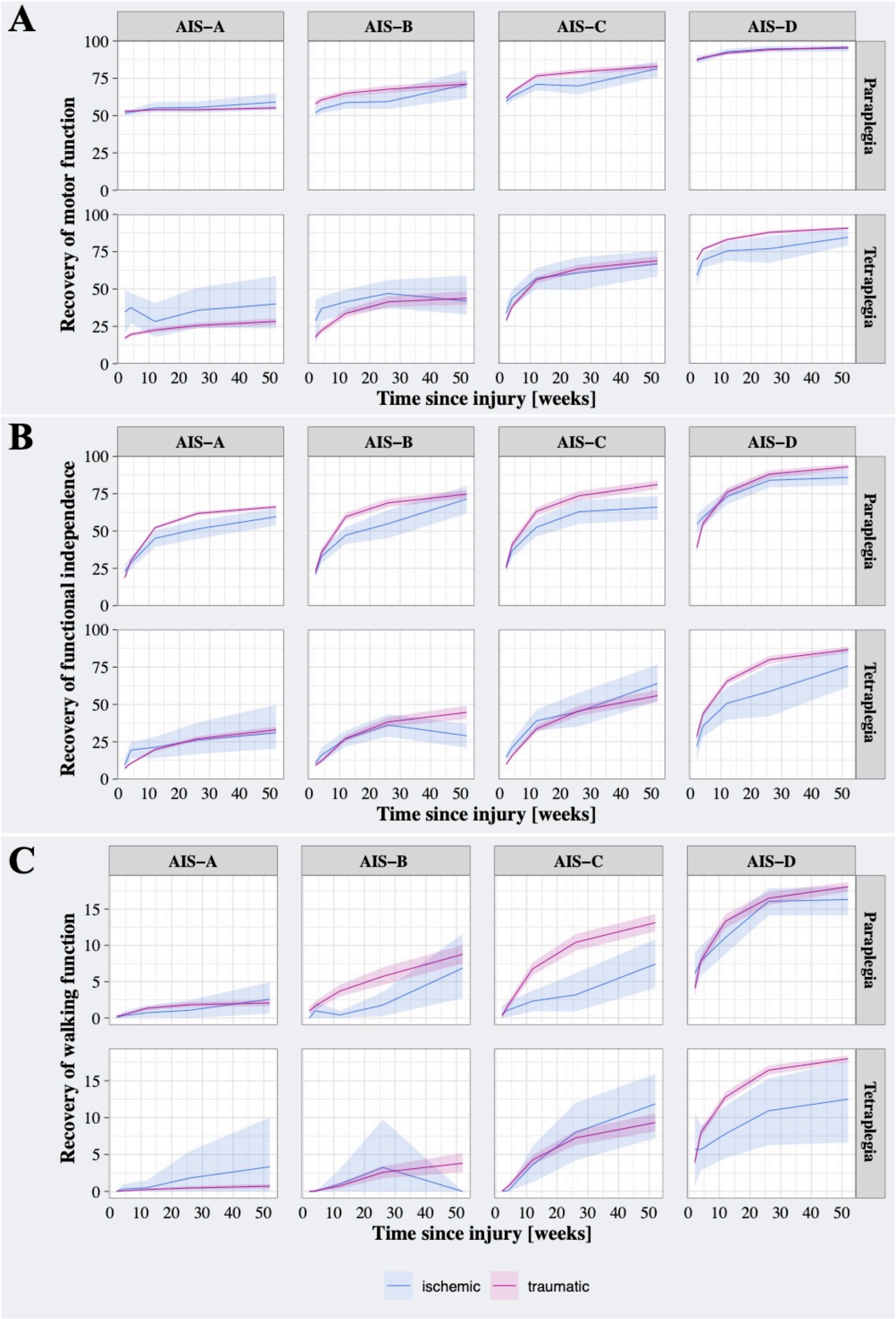

Additional File 2: Figure S13. Comparison of recovery profiles between patients with traumatic and ischemic injuries. The recovery profiles of **(A)** motor function (i.e., Total motor score), **(B)** functional independence (i.e., SCIM), and **(C)** walking function were comparable between patients with traumatic and ischemic spinal cord injuries.

SUPPLEMENTARY TABLES MAIN STUDY

Additional File 2: Table S1. Numbers and proportions of patients enrolled in the EMSCI per country (5-year bins).

	**2001-2005** (n=671)	**2006-2010** (n=1297)	**2011-2015** (n=1390)	**2016-2019** (n=1243)	**Overall** (n=4601)
**Country** (number of centers)	**n (%)**	**n (%)**	**n (%)**	**n (%)**	**n (%)**
Austria (2)	0 (0·0)	0 (0·0)	66 (4·7)	72 (5·8)	138 (3·0)
Czech Republic (1)	0 (0·0)	124 (9·6)	111 (8·0)	62 (5·0)	297 (6·5)
France (1)	43 (6·4)	3 (0·2)	0 (0·0)	0 (0·0)	46 (1·0)
Germany (14)	517 (77·0)	884 (68·2)	828 (59·6)	720 (57·9)	2949 (64·1)
Great Britain (2)	0 (0·0)	0 (0·0)	16 (1·2)	15 (1·2)	31 (0·7)
India (1)	0 (0·0)	0 (0·0)	15 (1·1)	47 (3·8)	62 (1·3)
Italy (2)	0 (0·0)	0 (0·0)	77 (5·5)	91 (7·3)	168 (3·7)
Netherlands (1)	45 (6·7)	48 (3·7)	40 (2·9)	37 (3·0)	170 (3·7)
Spain (2)	4 (0·6)	133 (10·3)	92 (6·6)	60 (4·8)	289 (6·3)
Switzerland (4)	62 (9·2)	105 (8·1)	145 (10·4)	139 (11·2)	451 (9·8)

Details for the centers can be found here: https://www.emsci.org/index.php/members

Additional File 2: Table S2. Demographics and injury characteristics of included EMSCI cohort stratified by age groups.

	0-29 years (N=1075)	30-59 years (N=2171)	60-89 years (N=1355)	Overall (N=4601)
**Sex**				
female	205 (19.1%)	449 (20.7%)	405 (29.9%)	1059 (23.0%)
male	870 (80.9%)	1722 (79.3%)	950 (70.1%)	3542 (77.0%)
**Age (years)**				
Mean (SD)	22.5 (4.08)	44.9 (8.62)	70.5 (7.21)	47.2 (19.0)
Median [Min, Max]	22.0 [9.00, 29.0]	46.0 [30.0, 59.0]	70.0 [60.0, 94.0]	47.0 [9.00, 94.0]
**Cause**				
Disc herniation	1 (0.1%)	6 (0.3%)	6 (0.4%)	13 (0.3%)
Haemorragic	0 (0%)	5 (0.2%)	10 (0.7%)	15 (0.3%)
Ischemic	29 (2.7%)	141 (6.5%)	161 (11.9%)	331 (7.2%)
Traumatic	1045 (97.2%)	2019 (93.0%)	1178 (86.9%)	4242 (92.2%)
**AIS Score**				
A	572 (53.2%)	894 (41.2%)	353 (26.1%)	1819 (39.5%)
B	158 (14.7%)	270 (12.4%)	126 (9.3%)	554 (12.0%)
C	153 (14.2%)	383 (17.6%)	335 (24.7%)	871 (18.9%)
D	192 (17.9%)	624 (28.7%)	541 (39.9%)	1357 (29.5%)
**Neurological level of injury**				
Cervical	484 (45.0%)	1036 (47.7%)	918 (67.7%)	2438 (53.0%)
Thoracic	447 (41.6%)	855 (39.4%)	341 (25.2%)	1643 (35.7%)
Lumbar	144 (13.4%)	280 (12.9%)	96 (7.1%)	520 (11.3%)

American Spinal Injury Association Impairment Scale (AIS):* ***AIS-A**, no sensory or motor function is preserved in the sacral segments S4-5.* ***AIS-B****, sensory but no motor function is preserved below the neurological level and includes the sacral segments S4-5 (LT or PP at S4-5 or DAP), and no motor function is preserved more than three levels below the motor level on either side of the body.* ***AIS-C****, motor function is preserved at the most caudal sacral segments for voluntary anal contraction OR the patient meets the criteria for sensory incomplete status, and has some sparing of motor function more than three levels below the ipsilateral motor level on either side of the body. Less than half of key muscle functions below the single NLI have a muscle grade ≥ 3.* ***AIS-D****, motor incomplete status as defined above, with at least half (half or more) of key muscle functions below the single NLI having a muscle grade ≥ 3.* ***AIS-E****, if sensation and motor function as tested with the ISNCSCI are graded as normal in all segments, and the patient had prior deficits, then the AIS grade is E. Someone without an initial SCI does not receive an AIS grade.*

Additional File 2: Table S3. Demographics and injury characteristics of *excluded* EMSCI cohort.

**Variable**	**Overall** (n=619)
**Sex, n (%)**	
Female	141 (22·8)
Male	477 (77·1)
Missing	1 (0·2)
**Age (years)**	
Mean (SD)	49·7 (20·5)
Median [Min, Max]	51·0 [13·0, 90·0]
Missing, n (%)	47 (7·6)
**Cause, n (%)**	
Disc herniation	1 (0·2)
Haemorrhagic	0 (0·0)
Ischemic	24 (3·9)
Traumatic	25 (4·0)
Other	569 (91·9)
**AIS grade*, n (%)**	
A (complete)	137 (22·1)
B (sensory incomplete)	35 (5·7)
C (motor incomplete)	54 (8·7)
D (motor incomplete)	140 (22·6)
E (normal)	10 (1·6)
Not tested	177 (28·6)
Missing	66 (10·7)
**Neurological level of injury, n (%)**	
Cervical	252 (40·7)
Thoracic	47 (7·6)
Lumbar	9 (1·5)
Sacral	68 (11·0)
Missing	243 (39·3)

American Spinal Injury Association Impairment Scale (AIS): ***AIS-A*, no sensory or motor function is preserved in the sacral segments S4-5.* ***AIS-B***, *sensory but no motor function is preserved below the neurological level and includes the sacral segments S4-5 (LT or PP at S4-5 or DAP), and no motor function is preserved more than three levels below the motor level on either side of the body.* ***AIS-C***, *motor function is preserved at the most caudal sacral segments for voluntary anal contraction OR the patient meets the criteria for sensory incomplete status, and has some sparing of motor function more than three levels below the ipsilateral motor level on either side of the body. Less than half of key muscle functions below the single NLI have a muscle grade ≥ 3.* ***AIS-D***, *motor incomplete status as defined above, with at least half (half or more) of key muscle functions below the single NLI having a muscle grade ≥ 3.* ***AIS-E***, *if sensation and motor function as tested with the ISNCSCI are graded as normal in all segments, and the patient had prior deficits, then the AIS grade is E. Someone without an initial SCI does not receive an AIS grade.*

Additional File 2: Table S4. Mean and standard deviation of age at injury for the entire EMSCI cohort between 2001 and 2019 stratified by sex.

**Year of Injury**	**Female**		**Male**	
	Age [years]		Age [years]	
	Mean	Standard deviation	Mean	Standard deviation
2001	58·00	26·68	43·60	23·06
2002	49·83	21·82	38·47	15·29
2003	44·90	21·00	38·87	16·97
2004	50·60	19·58	43·49	17·44
2005	54·43	21·16	44·53	18·97
2006	47·88	20·31	41·05	16·67
2007	43·98	18·44	42·53	17·71
2008	50·67	19·81	49·12	18·90
2009	46·65	20·18	43·24	16·88
2010	50·94	22·07	44·61	18·11
2011	52·92	21·28	45·94	17·75
2012	47·54	20·49	43·88	18·21
2013	51·85	19·63	47·93	18·16
2014	55·27	20·46	47·78	19·32
2015	49·55	21·09	48·90	19·27
2016	53·36	18·03	48·14	17·46
2017	50·96	19·74	50·10	18·85
2018	51·79	19·13	48·88	19·47
2019	59·07	19·42	48·59	18·36

Additional File 2: Table S5. Model output of longitudinal analysis of demographics (i.e., sex and age) and baseline injury characteristics (i.e., injury severity and level, plegia).

Model	Coefficients	Estimate	Std. Error	t-value	p-value	Adjusted p-value
Age distribution						
Overall (n = 4061)	Intercept	42·213	0·664	63·613	<0·001	Not applicable
	YEARDOI	8·6034	1·045	8·236	<0·001	Not applicable
Overall: Effect of sex (n = 4061)	Intercept	48·472	1·362	35·579	<0·001	Not applicable
	YEARDOI	4·486	2·123	2·113	0·035	Not applicable
	Sex	-8·101	1·557	-5·203	<0·001	Not applicable
	YEARDOI*Sex	5·306	2·433	2·181	0·029	Not applicable
Female:Paraplegia						
AIS-A (n = 216)	Intercept	38·983	3·154	12·359	<0·001	<0·001
	YEARDOI	5·997	4·933	1·216	0·225	
AIS-B (n = 76)	Intercept	41·856	4·724	8·861	<0·001	<0·001
	YEARDOI	3·226	7·314	0·441	0·660	
AIS-C (n = 94)	Intercept	49·702	4·786	10·385	<0·001	<0·001
	YEARDOI	-2·343	7·675	-0·305	0·761	
AIS-D (n = 130)	Intercept	51·929	4·784	10·855	<0·001	<0·001
	YEARDOI	-1·208	7·076	-0·171	0·865	
Female:Tetraplegia						
AIS-A (n = 144)	Intercept	45·942	3·546	12·956	<0·001	<0·001
	YEARDOI	10·684	5·984	1·785	0·076	
AIS-B (n = 60)	Intercept	46·885	4·853	9·661	<0·001	<0·001
	YEARDOI	4·325	7·703	0·561	0·577	
AIS-C (n = 133)	Intercept	53·101	4·097	12·960	<0·001	<0·001
	YEARDOI	7·791	6·599	1·181	0·240	
AIS-D (n = 206)	Intercept	60·045	3·258	18·432	<0·001	<0·001
	YEARDOI	-0·042	4·810	-0·009	0·993	
Male:Paraplegia						
AIS-A (n = 855)	Intercept	35·952	1·229	29·242	<0·001	<0·001
	YEARDOI	8·089	2·041	3·964	<0·001	0·001
AIS-B (n = 199)	Intercept	41·228	2·869	14·372	<0·001	<0·001
	YEARDOI	8·387	4·598	1·824	0·070	
AIS-C (n = 244)	Intercept	41·936	2·865	14·637	<0·001	<0·001
	YEARDOI	5·773	4·509	1·280	0·202	
AIS-D (n = 312)	Intercept	42·138	2·373	17·754	<0·001	<0·001
	YEARDOI	35·952	1·229	29·242	<0·001	<0·001
Male:Tetraplegia						
AIS-A (n = 604)	Intercept	4·091	3·557	1·150	0·251	
	YEARDOI	39·263	1·784	22·005	<0·001	<0·001
AIS-B (n = 219)	Intercept	5·781	2·904	1·991	0·047	
	YEARDOI	36·663	2·933	12·502	<0·001	<0·001
AIS-C (n = 400)	Intercept	3·841	4·778	0·804	0·422	
	YEARDOI	49·061	2·309	21·243	<0·001	<0·001
AIS-D (n = 709)	Intercept	6·786	3·496	1·941	0·053	
	YEARDOI	46·927	1·621	28·953	<0·001	<0·001
Sex ratio						
Overall	Intercept	3·383	0·389	8·692	<0·001	<0·001
	YEARDOI	0·102	0·665	0·153	0·880	
Injury severity						
AIS-A (n = 1819)	Intercept	3·725	0·638	5·835	<0·001	<0·001
	YEARDOI	0·694	1·091	0·636	0·533	
AIS-B (n = 554)	Intercept	3·260	1·303	2·502	0·023	0·092
	YEARDOI	1·223	2·226	0·549	0·522	
AIS-C (n = 871)	Intercept	2·641	0·861	3·069	0·007	0·028
	YEARDOI	0·925	1·490	0·621	0·543	
AIS-D (n = 1357)	Intercept	3·561	0·547	6·515	<0·001	<0·001
	YEARDOI	-0·376	0·934	-0·402	0·692	
Plegia						
Paraplegia (n = 2126)	Intercept	3·852	0·702	5·491	<0·001	<0·001
	YEARDOI	-0·911	1·246	-0·731	0·475	
Tetraplegia (n = 2475)	Intercept	3·129	0·334	9·363	<0·001	<0·001
	YEARDOI	0·895	0·571	1·568	0·135	
AIS grade						
Overall						
AIS-A (n = 1819)	Intercept	22·654	4·222	5·366	<0·001	<0·001
	YEARDOI	-5·252	7·117	-0·738	0·466	
AIS-B (n = 554)	Intercept	6·685	1·196	5·588	<0·001	<0·001
	YEARDOI	-1·213	1·990	-0·610	0·546	
AIS-C (n = 871)	Intercept	10·962	1·693	6·474	<0·001	<0·001
	YEARDOI	-2·241	2·893	-0·775	0·444	
AIS-D (n = 1357)	Intercept	13·207	2·587	5·105	<0·001	<0·001
	YEARDOI	3·586	4·420	0·811	0·422	
Female: Paraplegia						
AIS-A (n = 216)	Intercept	4·408	0·591	7·463	<0·001	<0·001

	YEARDOI	0·481	0·982	0·489	0·631	
AIS-B (n = 76)	Intercept	2·109	0·571	3·694	0·002	0·035
	YEARDOI	-0·598	0·934	-0·640	0·532	
AIS-C (n = 94)	Intercept	3·572	0·617	5·789	<0·001	<0·001
	YEARDOI	-2·127	1·035	-2·056	0·057	
AIS-D (n = 130)	Intercept	2·101	0·735	2·859	0·011	
	YEARDOI	1·252	1·222	1·025	0·321	
Female: Tetraplegia						
AIS-A (n = 144)	Intercept	4·786	0·713	6·708	<0·001	<0·001
	YEARDOI	-2·687	1·153	-2·330	0·034	
AIS-B (n = 60)	Intercept	1·940	0·284	6·836	<0·001	<0·001
	YEARDOI	-0·939	0·470	-1·999	0·064	
AIS-C (n = 133)	Intercept	4·256	0·600	7·090	<0·001	<0·001
	YEARDOI	-2·134	1·026	-2·080	0·053	
AIS-D (n = 206)	Intercept	5·184	1·210	4·284	0·001	0·008
	YEARDOI	-0·694	2·067	-0·335	0·741	
Male: Paraplegia						
AIS-A (n = 855)	Intercept	20·380	1·906	10·691	<0·001	<0·001
	YEARDOI	-3·731	3·257	-1·146	0·268	
AIS-B (n = 199)	Intercept	4·903	0·817	5·998	<0·001	<0·001
	YEARDOI	-0·894	1·359	-0·658	0·520	
AIS-C (n = 244)	Intercept	5·481	0·876	6·260	<0·001	<0·001
	YEARDOI	-0·415	1·456	-0·285	0·779	
AIS-D (n = 312)	Intercept	5·886	0·923	6·380	<0·001	<0·001
	YEARDOI	1·882	1·576	1·194	0·249	
Male: Tetraplegia						
AIS-A (n = 604)	Intercept	13·973	1·319	10·597	<0·001	<0·001
	YEARDOI	-1·923	2·253	-0·853	0·405	
AIS-B (n = 219)	Intercept	4·834	0·641	7·540	<0·001	<0·001
	YEARDOI	-0·327	1·066	-0·307	0·763	
AIS-C (n = 400)	Intercept	10·220	1·629	6·274	<0·001	<0·001
	YEARDOI	-1·807	2·783	-0·649	0·525	
AIS-D (n = 709)	Intercept	13·243	1·414	9·366	<0·001	<0·001
	YEARDOI	4·460	2·416	1·846	0·082	

Additional File 2: Table S6. Overview of longitudinal sensory and motor recovery. Patients enrolled in the EMSCI had 5 follow-up time points, while the patients participating the Sygen trial had seven. Upper extremity motor scores were computed for paraplegic patients only. We report mean (standard deviation); number of patients.

	Paraplegic Spinal Cord Injury				Tetraplegic Spinal Cord Injury			
	AIS-A	AIS-B	AIS-C	AIS-D	AIS-A	AIS-B	AIS-C	AIS-D
EMSCI								
**Lower Extremity Motor Score**								
Baseline (2 weeks)	2·834 (8·679); n=586	7·132 (12·542); n=159	11·227 (7·596); n=186	37·125 (9·579); n=234	1·200 (5·305); n=402	0·026 (0·229); n=151	12·704 (10·225); n=304	38·444 (11·293); n=558
4 weeks	2·992 (8·794); n=934	9·407 (13·592); n=242	15·446 (9·926); n=306	38·668 (8·415); n=377	2·015 (7·062); n=662	2·791 (7·826); n=258	17·553 (13·574); n=464	40·846 (9·794); n=807
12 weeks	4·008 (10·291); n=877	13·861 (13·770); n=209	25·679 (13·042); n=279	42·051 (7·819); n=279	3·135 (9·123); n=602	9·062 (13·304); n=243	28·023 (14·629); n=397	43·420 (8·414); n=562
26 Weeks	4·003 (10·065); n=614	16·510 (14·728); n=145	28·115 (13·967); n=183	44·286 (6·307); n=191	4·013 (10·786); n=468	13·208 (16·189); n=184	31·435 (14·592); n=271	45·225 (8·005); n=377
52 Weeks	5·323 (11·957); n=576	21·116 (15·359); n=147	32·688 (12·660); n=171	45·609 (5·536); n=199	4·181 (11·701); n=368	14·311 (17·301); n=119	33·957 (14·445); n=236	46·538 (5·501); n=380
**Upper Extremity Motor Score**								
Baseline (2 weeks)	--	--	--	--	16·343 (15·083); n=402	18·340 (13·982); n=151	16·668 (11·299); n=304	30·978 (10·813); n=558
4 weeks	--	--	--	--	18·099 (15·802); n=662	19·946 (14·568); n=258	21·063 (12·415); n=464	35·501 (9·697); n=807
12 weeks	--	--	--	--	19·542 (14·973); n=602	24·850 (13·668); n=243	28·117 (11·273); n=397	39·556 (7·885); n=562
26 Weeks	--	--	--	--	21·860 (15·568); n=468	28·288 (13·955); n=184	31·951 (11·263); n=271	42·357 (7·008); n=377
52 Weeks	--	--	--	--	24·416 (14·869); n=368	29·462 (12·411); n=119	34·952 (10·792); n=236	44·116 (5·710); n=380
**Total Motor Score**								
Baseline (2 weeks)	52·834 (8·679); n=586	57·132 (12·542); n=159	61·227 (7·596); n=186	87·125 (9·579); n=234	17·534 (16·439); n=402	18·340 (13·982); n=151	29·369 (13·334); n=304	69·173 (16·903); n=558
4 weeks	52·992 (8·794); n=934	59·407 (13·592); n=242	65·446 (9·926); n=306	88·668 (8·415); n=377	20·080 (18·024); n=662	22·713 (17·053); n=258	38·606 (19·287); n=464	76·363 (15·429); n=807
12 weeks	54·008 (10·291); n=877	63·861 (13·770); n=209	75·679 (13·042); n=279	92·051 (7·819); n=279	22·625 (18·624); n=602	33·879 (21·132); n=243	56·181 (21·659); n=397	82·830 (13·071); n=562
26 Weeks	54·003 (10·065); n=614	66·510 (14·728); n=145	78·115 (13·967); n=183	94·286 (6·307); n=191	25·809 (20·600); n=468	41·568 (24·199); n=184	63·459 (22·232); n=271	87·540 (12·054); n=377
52 Weeks	55·323 (11·957); n=576	71·116 (15·359); n=147	82·688 (12·660); n=171	95·609 (5·536); n=199	28·460 (21·014); n=368	43·773 (24·804); n=119	68·763 (22·401); n=236	90·634 (9·371); n=380
**Total Pin Prick**								
Baseline (2 weeks)	63·888 (15·831); n=586	81·277 (18·582); n=159	86·728 (16·073); n=186	86·728 (16·073); n=186	22·977 (14·185); n=402	39·113 (23·953); n=151	52·003 (28·560); n=304	75·608 (29·187); n=558
4 weeks	63·861 (15·994); n=934	80·565 (19·544); n=242	88·987 (15·352); n=306	88·987 (15·352); n=306	25·340 (16·630); n=662	38·976 (24·898); n=258	57·658 (29·828); n=464	79·947 (27·686); n=807
12 weeks	65·125 (16·833); n=877	82·029 (19·296); n=209	91·605 (15·368); n=279	91·605 (15·368); n=279	28·062 (18·346); n=602	46·842 (27·613); n=243	66·311 (28·949); n=397	84·056 (25·519); n=562
26 Weeks	64·201 (16·574); n=614	82·910 (18·978); n=145	93·060 (14·143); n=183	93·060 (14·143); n=183	27·772 (17·533); n=468	51·716 (29·404); n=184	67·186 (28·089); n=271	83·307 (26·282); n=377

52 Weeks	64·812 (16·844); n=576	83·781 (19·233); n=147	92·164 (15·315); n=171	92·164 (15·315); n=171	28·279 (17·461); n=368	51·127 (27·618); n=119	66·359 (28·289); n=236	82·193 (25·754); n=380
**Total Light Touch**								
Baseline (2 weeks)	65·409 (15·841); n=586	87·019 (15·625); n=159	92·546 (12·363); n=186	99·227 (10·456); n=234	26·955 (16·117); n=402	57·807 (21·996); n=151	68·490 (22·631); n=304	86·884 (21·130); n=558
4 weeks	65·896 (16·144); n=934	89·243 (15·479); n=242	94·807 (12·335); n=306	100·401 (9·943); n=377	31·861 (19·396); n=662	63·278 (22·287); n=258	75·943 (22·219); n=464	91·065 (19·838); n=807
12 weeks	67·540 (17·102); n=877	91·488 (14·561); n=209	97·246 (12·287); n=279	100·978 (9·821); n=279	36·600 (21·618); n=602	70·404 (23·717); n=243	82·952 (21·236); n=397	93·418 (18·814); n=562
26 Weeks	66·497 (17·169); n=614	90·667 (15·618); n=145	97·835 (11·653); n=183	101·784 (8·889); n=191	36·729 (21·253); n=468	72·749 (23·415); n=184	82·204 (20·061); n=271	92·738 (19·197); n=377
52 Weeks	67·430 (17·639); n=576	91·870 (13·810); n=147	98·287 (11·222); n=171	102·670 (8·764); n=199	38·564 (21·829); n=368	72·093 (22·374); n=119	81·647 (20·136); n=236	90·921 (19·491); n=380
**Total Sensory Score**								
Baseline (2 weeks)	98·131 (23·768); n=586	130·424 (23·373); n=159	138·770 (18·958); n=186	148·760 (15·877); n=234	40·424 (24·130); n=402	86·520 (33·184); n=151	103·030 (34·136); n=304	129·942 (31·988); n=558
4 weeks	98·857 (24·198); n=934	133·824 (23·100); n=242	142·136 (18·744); n=306	150·564 (15·013); n=377	47·625 (29·089); n=662	94·945 (33·647); n=258	113·906 (33·462); n=464	136·455 (29·944); n=807
12 weeks	101·341 (25·600); n=877	137·182 (21·742); n=209	145·870 (18·585); n=279	151·447 (14·931); n=279	54·702 (32·437); n=602	105·475 (35·776); n=243	124·360 (32·019); n=397	140·031 (28·430); n=562
26 Weeks	99·728 (25·710); n=614	135·896 (23·186); n=145	146·731 (17·532); n=183	152·753 (13·445); n=191	55·009 (31·823); n=468	108·874 (35·015); n=184	123·524 (30·613); n=271	138·947 (29·017); n=377
52 Weeks	101·183 (26·384); n=576	137·760 (20·451); n=147	147·503 (16·887); n=171	153·990 (13·228); n=199	57·787 (32·721); n=368	108·169 (33·559); n=119	122·384 (30·543); n=236	136·483 (29·334); n=380
Sygen Trial								
**Lower Extremity Motor Score**								
Baseline (<72 hours)	0·073 (0·726); n=128	0 (0); n=8	2·273 (5·179); n=13	13·667 (12·662); n=3	0 (0); n=297	0·544 (2·384); n=69	6·404 (11·431); n=130	17·952 (14·179); n=28
1 week	0 (0); n=132	0 (0); n=8	4·154 (7·437); n=13	22·667 (8·505); n=3	0·095 (0·802); n=314	0 (0); n=69	8·425 (8·663); n=136	27·8 (7·703); n=28
4 weeks	0·271 (2·766); n=125	4·143 (5·956); n=8	17·5 (18·495); n=11	41 (7·81); n=3	0·638 (3·502); n=290	5·621 (11·242); n=63	19·235 (14·519); n=127	39·435 (7·372); n=24
8 weeks	0·423 (3·713); n=126	10·571 (10·814); n=8	22·8 (19·826); n=11	45·667 (4·933); n=3	1·062 (4·664); n=281	9·182 (14·766); n=60	27·259 (16·22); n=121	43·56 (7·583); n=26
16 weeks	0·417 (3·072); n=121	24·167 (7·36); n=6	26·6 (20·592); n=10	48 (1); n=3	1·961 (7·044); n=267	13·482 (17·17); n=60	32·782 (16·183); n=113	45·333 (4·705); n=26
26 Weeks	0·624 (4·409); n=124	25·857 (10·961); n=7	27·273 (21·868); n=12	49 (1·732); n=3	2·073 (7·187); n=277	16·018 (18·023); n=62	35·953 (16·481); n=121	45·769 (4·91); n=26
52 Weeks	0·718 (4·759); n=123	28 (14·855); n=7	25·75 (22·503); n=12	47·667 (3·215); n=3	2·619 (8·276); n=263	17·5 (19·307); n=56	37·922 (15·053); n=110	46·348 (5·322); n=25
**Upper Extremity Motor Score**								
Baseline (<72 hours)	--	--	--	--	10·872 (11·913); n=297	14·468 (13·235); n=69	15·289 (14·167); n=130	26·667 (13·638); n=28
1 week	--	--	--	--	12·099 (11·041); n=314	15·785 (11·501); n=69	14·762 (11·291); n=136	31·269 (10·429); n=28
4 weeks	--	--	--	--	14·015 (11·909); n=290	18·5 (12·913); n=63	21·311 (11·822); n=127	38·083 (8·812); n=24
8 weeks	--	--	--	--	15·871 (12·63); n=281	22·204 (12·768); n=60	28·596 (11·403); n=121	42·462 (7·021); n=26

16 weeks	--	--	--	--	18·024 (12·866); n=267	26·519 (13·336); n=60	33·786 (11·775); n=113	43·909 (5·52); n=26
26 Weeks	--	--	--	--	19·816 (13·1); n=277	27·054 (13·416); n=62	36·771 (11·181); n=121	45·423 (5·721); n=26
52 Weeks	--	--	--	--	21·764 (12·994); n=263	30·529 (14·008); n=56	38·624 (11·075); n=110	47·043 (4·15); n=25
**Total Motor Score**								
Baseline (<72 hours)	41·852 (16·001); n=128	49·75 (0·707); n=8	48·846 (15·566); n=13	51·667 (22·546); n=3	10·997 (11·29); n=297	15·261 (13·485); n=69	21·769 (18·373); n=130	42·333 (18·47); n=28
1 week	45·455 (9·601); n=132	48·25 (3·059); n=8	50·231 (11·359); n=13	64·667 (13·204); n=3	12·169 (10·921); n=314	16·29 (11·762); n=69	23·485 (13·401); n=136	58·25 (10·298); n=28
4 weeks	46·918 (8·903); n=125	49·286 (15·734); n=8	65·2 (18·902); n=11	89 (6·557); n=3	14·493 (12·587); n=290	24·066 (19·574); n=63	40·919 (22·116); n=127	76·875 (13·129); n=24
8 weeks	48·252 (7·595); n=126	60·571 (10·814); n=8	71·2 (21·259); n=11	94·667 (4·509); n=3	16·908 (14·073); n=281	31·964 (22·573); n=60	55·282 (24·201); n=121	85·269 (11·323); n=26
16 weeks	48·808 (6·626); n=121	74·167 (7·36); n=6	73·9 (25·071); n=10	97 (2); n=3	19·835 (16·236); n=267	39·897 (25·551); n=60	66·179 (24·601); n=113	87·609 (10·188); n=26
26 Weeks	49·839 (6·173); n=124	75·857 (10·961); n=7	77 (22·213); n=12	98·333 (1·528); n=3	21·86 (16·432); n=277	43·772 (27·432); n=62	72·055 (25·07); n=121	91·192 (8·727); n=26
52 Weeks	49·797 (6·576); n=123	78 (14·855); n=7	75·25 (23·105); n=12	97 (3); n=3	24·619 (17·32); n=263	48·093 (28·495); n=56	74·611 (25·371); n=110	93·391 (7·838); n=25
**Total Pin Prick**								
Baseline (<72 hours)	--	--	--	--	--	--	--	--
1 week	49·053 (10·858); n=132	67·625 (10·555); n=8	64·154 (13·957); n=13	62 (10·536); n=3	18·322 (8·813); n=314	34·232 (17·619); n=69	46·067 (27·12); n=136	69·179 (26·031); n=28
4 weeks	50·631 (10·514); n=125	75·714 (11·324); n=8	71·7 (19·63); n=11	90·667 (24·379); n=3	21·523 (13·821); n=290	52 (27·621); n=63	67·213 (27·507); n=127	78·125 (18·889); n=24
8 weeks	51·317 (11·219); n=126	87·429 (17·453); n=8	74·4 (20·293); n=11	91·667 (21·939); n=3	23·607 (15·149); n=281	54·982 (29·088); n=60	74·59 (27·7); n=121	84·923 (18·249); n=26
16 weeks	52·375 (12·013); n=121	93 (13·565); n=6	79·1 (21·341); n=10	94·667 (19·858); n=3	26·413 (18·82); n=267	58·069 (30·166); n=60	78·019 (28·807); n=113	90·909 (15·181); n=26
26 Weeks	52·568 (12·193); n=124	94·857 (12·469); n=7	78 (20·651); n=12	95·667 (16·258); n=3	26·812 (18·908); n=277	60·138 (30·098); n=62	79·445 (27·466); n=121	93·923 (12·809); n=26
52 Weeks	53·059 (12·503); n=123	97·286 (12·539); n=7	81·5 (20·03); n=12	100·667 (8·737); n=3	28·052 (19·709); n=263	62·963 (29·914); n=56	81·84 (27·724); n=110	90·913 (17·876); n=25
**Total Light Touch**	49·053 (10·858); n=132	67·625 (10·555); n=8	64·154 (13·957); n=13	62 (10·536); n=3	18·322 (8·813); n=314	34·232 (17·619); n=69	46·067 (27·12); n=136	69·179 (26·031); n=28
Baseline (<72 hours)	--	--	--	--	--	--	--	--
1 week	50·267 (10·223); n=132	68·625 (19·878); n=8	76·692 (8·42); n=13	93·333 (16·289); n=3	19·895 (9·646); n=314	46·58 (22·112); n=69	60·711 (29·175); n=136	84·036 (22·622); n=28
4 weeks	52·352 (11·029); n=125	72·429 (17·803); n=8	86·1 (21·377); n=11	111·333 (0·577); n=3	25·931 (17·177); n=290	66·167 (24·877); n=63	78·279 (27·08); n=127	94·542 (16·577); n=24
8 weeks	53·114 (11·249); n=126	85·429 (18·831); n=8	90·3 (20·26); n=11	111·333 (1·155); n=3	29·37 (19·322); n=281	70·614 (25·174); n=60	84·897 (26·597); n=121	96·577 (15·867); n=26
16 weeks	53·842 (11·965); n=121	92 (16·923); n=6	91·8 (19·263); n=10	112 (0); n=3	32·408 (22·867); n=267	74·879 (28·215); n=60	86·406 (25·75); n=113	95·773 (14·534); n=26
26 Weeks	54·729 (12·193); n=124	95·143 (13·496); n=7	92·7 (19·316); n=12	111·333 (1·155); n=3	33·827 (23·141); n=277	73·621 (28·267); n=62	88·464 (24·564); n=121	98·962 (12·565); n=26

52 Weeks	55·068 (13·57); n=123	98·429 (11·717); n=7	93·5 (15·205); n=12	111 (1·732); n=3	35·492 (24·46); n=263	74·944 (26·714); n=56	88·896 (26·077); n=110	99·957 (13·736); n=25
**Total Sensory Score**								
Baseline (<72 hours)	--	--	--	--	--	--	--	--
1 week	75·45 (15·436); n=132	104·75 (29·918); n=8	115·385 (12·031); n=13	142·333 (22·898); n=3	29·812 (14·539); n=314	69·638 (33·132); n=69	91·311 (43·893); n=136	126·286 (34·657); n=28
4 weeks	78·566 (16·573); n=125	108·714 (26·644); n=8	128·8 (31·545); n=11	167·333 (0·577); n=3	39·069 (25·944); n=290	99·6 (37·97); n=63	117·352 (40·661); n=127	142·333 (24·343); n=24
8 weeks	79·821 (17·013); n=126	130·714 (27·171); n=8	135·5 (30·376); n=11	167·333 (1·155); n=3	44·074 (29·189); n=281	105·912 (37·85); n=60	127·41 (39·859); n=121	145·731 (23·521); n=26
16 weeks	80·983 (18·001); n=121	138·167 (25·294); n=6	137·3 (28·945); n=10	168 (0); n=3	48·408 (34·214); n=267	112·466 (42·496); n=60	129·84 (38·721); n=113	144·182 (21·551); n=26
26 Weeks	82·28 (18·162); n=124	143·143 (20·506); n=7	138·8 (29·123); n=12	167·333 (1·155); n=3	50·658 (34·649); n=277	110·5 (42·386); n=62	133·2 (36·917); n=121	148·692 (19·091); n=26
52 Weeks	82·898 (20·22); n=123	149·286 (17·433); n=7	139·5 (22·841); n=12	167 (1·732); n=3	53·167 (36·658); n=263	112·87 (40·156); n=56	133·698 (39·306); n=110	151·348 (20·239); n=25

Additional File 2: Table S7. Model output of lower extremity motor score (LEMS) stratified by sex, plegia, and baseline AIS grades. Patients were enrolled in the EMSCI study. Significant values are highlighted in red.

**Model**	**Variable**	**Estimate**	**Standard Error**	**DF**	**t-value**	**p-value**	**Adjusted p-value**
Female *paraplegic* patients							
AIS-A (n = 228)	Intercept	2·983	1·737	247·776	1·717	0·087	
	Age at injury	-3·179	2·662	228·839	-1·194	0·234	
	Time since injury[#]	2·642	1·176	478·410	2·246	0·025	0·402
	YEARDOI	1·918	2·316	257·542	0·828	0·408	
	Time since injury*YEARDOI	-1·349	2·073	483·681	-0·651	0·516	
AIS-B (n = 76)	Intercept	15·233	4·650	74·316	3·276	0·002	0·026
	Age at injury	-12·508	7·493	71·474	-1·669	0·100	
	Time since injury	11·780	2·551	156·940	4·619	<0·001	<0·001
	YEARDOI	1·548	5·661	77·390	0·274	0·785	
	Time since injury*YEARDOI	1·669	4·515	159·136	0·370	0·712	
AIS-C (n = 98)	Intercept	22·028	3·216	114·399	6·850	<0·001	<0·001
	Age at injury	-4·382	4·300	100·408	-1·019	0·311	
	Time since injury	21·327	3·207	222·061	6·650	<0·001	<0·001
	YEARDOI	-6·571	4·065	128·566	-1·617	0·108	
	Time since injury*YEARDOI	-3·587	5·691	227·545	-0·630	0·529	
AIS-D (n = 141)	Intercept	37·898	2·062	159·886	18·377	<0·001	<0·001
	Age at injury	-3·948	2·477	137·746	-1·594	0·113	
	Time since injury	9·003	2·258	262·082	3·988	<0·001	0·001
	YEARDOI	4·833	2·481	177·919	1·948	0·053	
	Time since injury*YEARDOI	-1·327	3·855	283·477	-0·344	0·731	
Female *tetraplegic* patients							
AIS-A (n = 159)	Intercept	1·283	1·972	178·806	0·651	0·516	
	Age at injury	3·358	2·930	165·254	1·146	0·253	
	Time since injury	1·826	1·548	376·178	1·179	0·239	
	YEARDOI	-1·749	2·606	193·144	-0·671	0·503	
	Time since injury*YEARDOI	5·323	2·961	385·724	1·798	0·073	
AIS-B (n = 65)	Intercept	2·735	3·711	80·821	0·737	0·463	
	Age at injury	0·734	5·775	65·931	0·127	0·899	
	Time since injury	5·082	3·670	156·139	1·385	0·168	
	YEARDOI	-0·219	4·421	90·708	-0·050	0·961	
	Time since injury*YEARDOI	23·013	6·941	158·657	3·315	0·001	0·018
AIS-C (n = 138)	Intercept	18·713	3·253	147·400	5·753	<0·001	<0·001
	Age at injury	0·355	4·166	130·016	0·085	0·932	
	Time since injury	17·525	2·917	291·653	6·008	<0·001	<0·001
	YEARDOI	-4·021	3·924	170·967	-1·025	0·307	
	Time since injury*YEARDOI	5·265	5·168	303·414	1·019	0·309	
AIS-D (n = 223)	Intercept	39·133	2·282	246·104	17·147	<0·001	<0·001
	Age at injury	-5·590	2·743	233·116	-2·038	0·043	0·684
	Time since injury	10·019	1·883	411·718	5·321	<0·001	<0·001
	YEARDOI	5·736	2·299	264·044	2·495	0·013	0·212
	Time since injury*YEARDOI	-4·061	2·934	418·885	-1·384	0·167	
Male *paraplegic* patients							
AIS-A (n = 904)	Intercept	1·981	0·972	914·106	2·038	0·042	0·670
	Age at injury	1·267	1·789	890·680	0·708	0·479	
	Time since injury	2·702	0·448	2004·930	6·031	<0·001	<0·001

	YEARDOI	1·654	1·315	937·993	1·258	0·209	
	Time since injury*YEARDOI	-0·092	0·812	2015·832	-0·113	0·910	
AIS-B (n = 209)	Intercept	5·948	2·662	219·897	2·235	0·026	0·423
	Age at injury	3·935	4·247	199·615	0·927	0·355	
	Time since injury	9·774	2·008	468·974	4·868	<0·001	<0·001
	YEARDOI	2·126	3·454	234·103	0·615	0·539	
	Time since injury*YEARDOI	8·730	3·624	475·405	2·409	0·016	0·262
AIS-C (n = 263)	Intercept	18·886	1·996	319·958	9·462	<0·001	<0·001
	Age at injury	-4·512	2·923	255·218	-1·544	0·124	
	Time since injury	16·287	2·202	609·466	7·395	<0·001	<0·001
	YEARDOI	-1·909	2·648	357·814	-0·721	0·472	
	Time since injury*YEARDOI	7·285	3·674	613·114	1·983	0·048	0·765
AIS-D (n = 358)	Intercept	41·186	1·201	380·477	34·292	<0·001	<0·001
	Age at injury	-6·661	1·792	324·152	-3·717	<0·001	0·004
	Time since injury	7·823	1·324	596·929	5·907	<0·001	<0·001
	YEARDOI	0·674	1·479	419·434	0·456	0·649	
	Time since injury*YEARDOI	1·703	2·238	635·148	0·761	0·447	
Male *tetraplegic* patients							
AIS-A (n = 634)	Intercept	-1·840	0·863	720·530	-2·131	0·033	0·535
	Age at injury	6·784	1·337	650·124	5·074	<0·001	<0·001
	Time since injury	2·191	0·767	1444·351	2·859	0·004	0·069
	YEARDOI	1·432	1·182	774·175	1·211	0·226	
	Time since injury*YEARDOI	1·820	1·361	1467·202	1·337	0·181	
AIS-B (n = 229)	Intercept	3·489	2·004	317·841	1·741	0·083	
	Age at injury	-0·567	3·108	259·952	-0·183	0·855	
	Time since injury	15·560	2·597	592·538	5·993	<0·001	<0·001
	YEARDOI	-1·233	2·829	351·871	-0·436	0·663	
	Time since injury*YEARDOI	-0·638	4·694	603·163	-0·136	0·892	
AIS-C (n = 410)	Intercept	18·137	2·068	480·814	8·769	<0·001	<0·001
	Age at injury	-1·726	2·788	423·890	-0·619	0·536	
	Time since injury	17·475	2·051	935·816	8·521	<0·001	<0·001
	YEARDOI	1·132	2·457	539·023	0·461	0·645	
	Time since injury*YEARDOI	6·549	3·461	956·361	1·892	0·059	
AIS-D (n = 727)	Intercept	40·903	1·045	744·568	39·142	<0·001	<0·001
	Age at injury	-1·847	1·519	690·554	-1·216	0·225	
	Time since injury	9·282	0·929	1397·151	9·995	<0·001	<0·001
	YEARDOI	1·263	1·231	808·532	1·026	0·305	
	Time since injury*YEARDOI	-3·711	1·560	1427·739	-2·379	0·018	0·280

[#]Time since injury measured in weeks: 2, 4, 8, 16, 26, and 52 weeks. DF: degree of freedom; YEARDOI: Year of injury

Additional File 2: Table S8. Model output of upper extremity motor score (UEMS) stratified by sex, plegia, and baseline AIS grades. Patients were enrolled in the EMSCI study. Significant values are highlighted in red.
Note: The model was only run for tetraplegic patients.

Model	Variable	Estimate	Standard Error	DF	t-value	p-value	Adjusted p-value
Female *tetraplegic* patients							
AIS-A (n = 159)	Intercept	23·354	3·674	157·787	6·356	<0·001	<0·001
	Age at injury	-13·601	5·534	154·294	-2·458	0·015	0·121
	Time since injury[#]	8·696	1·466	348·863	5·934	<0·001	<0·001
	YEARDOI	6·752	4·777	160·496	1·413	0·160	
	Time since injury*YEARDOI	0·007	2·809	351·387	0·003	0·998	
AIS-B (n = 65)	Intercept	20·813	4·793	62·535	4·342	<0·001	<0·001
	Age at injury	2·084	7·813	59·808	0·267	0·791	
	Time since injury	10·183	2·115	140·960	4·815	<0·001	<0·001
	YEARDOI	-2·848	5·570	63·984	-0·511	0·611	
	Time since injury*YEARDOI	6·182	4·016	141·507	1·539	0·126	
AIS-C (n = 137)	Intercept	27·107	2·615	133·461	10·367	<0·001	<0·001
	Age at injury	-8·207	3·336	115·083	-2·461	0·015	0·123
	Time since injury	16·166	2·555	276·246	6·328	<0·001	<0·001
	YEARDOI	-1·701	3·168	157·955	-0·537	0·592	
	Time since injury*YEARDOI	2·790	4·481	289·243	0·623	0·534	
AIS-D (n = 225)	Intercept	39·903	1·967	240·270	20·292	<0·001	<0·001
	Age at injury	-7·093	2·367	222·907	-2·997	0·003	0·024
	Time since injury	10·809	1·819	411·321	5·943	<0·001	<0·001
	YEARDOI	0·620	1·986	264·090	0·312	0·755	
	Time since injury*YEARDOI	-0·074	2·839	422·452	-0·026	0·979	
Male *tetraplegic* patients							
AIS-A (n = 636)	Intercept	15·298	1·816	637·805	8·423	<0·001	<0·001
	Age at injury	3·007	2·867	626·405	1·049	0·295	
	Time since injury	7·973	0·703	1344·402	11·337	<0·001	<0·001
	YEARDOI	5·440	2·445	647·185	2·225	0·027	0·212
	Time since injury*YEARDOI	0·520	1·252	1349·615	0·415	0·678	
AIS-B (n = 228)	Intercept	18·165	2·779	230·796	6·538	<0·001	<0·001
	Age at injury	-7·183	4·528	221·651	-1·586	0·114	
	Time since injury	13·060	1·626	519·060	8·035	<0·001	<0·001
	YEARDOI	9·675	3·827	236·538	2·528	0·012	0·097
	Time since injury*YEARDOI	-0·412	2·953	521·469	-0·140	0·889	
AIS-C (n = 411)	Intercept	30·405	1·796	421·570	16·927	<0·001	<0·001
	Age at injury	-15·934	2·442	382·749	-6·525	<0·001	<0·001
	Time since injury	15·173	1·557	873·718	9·747	<0·001	<0·001
	YEARDOI	-1·675	2·113	462·733	-0·793	0·429	
	Time since injury*YEARDOI	4·810	2·632	891·559	1·828	0·068	0·544
AIS-D (n = 723)	Intercept	36·320	1·023	775·280	35·509	<0·001	<0·001
	Age at injury	-6·086	1·476	695·751	-4·124	<0·001	<0·001
	Time since injury	13·402	1·055	1453·049	12·704	<0·001	<0·001
	YEARDOI	2·292	1·216	869·473	1·885	0·060	
	Time since injury*YEARDOI	-1·228	1·768	1490·395	-0·695	0·487	

[#]Time since injury measured in weeks: 2, 4, 8, 16, 26, and 52 weeks· DF: degree of freedom; YEARDOI: Year of injury

Additional File 2: Table S9. Model output of upper extremity motor score (UEMS) stratified by sex, plegia, and baseline AIS grades. Patients were enrolled in the EMSCI study. Significant values are highlighted in red.
Note: The model was only run for tetraplegic patients.

Model	Variable	Estimate	Standard Error	DF	t-value	p-value	Adjusted p-value
Female *paraplegic* patients							
AIS-A (n = 228)	Intercept	52·983	1·737	247·776	30·505	<0·001	<0·001
	Age at injury	-3·179	2·662	228·839	-1·194	0·234	
	Time since injury[#]	2·642	1·176	478·410	2·246	0·025	0·402
	YEARDOI	1·918	2·316	257·542	0·828	0·408	
	Time since injury*YEARDOI	-1·349	2·073	483·681	-0·651	0·516	
AIS-B (n = 76)	Intercept	65·233	4·650	74·316	14·030	<0·001	<0·001
	Age at injury	-12·508	7·493	71·474	-1·669	0·100	
	Time since injury	11·780	2·551	156·940	4·619	<0·001	<0·001
	YEARDOI	1·548	5·661	77·390	0·274	0·785	
	Time since injury*YEARDOI	1·669	4·515	159·136	0·370	0·712	
AIS-C (n = 98)	Intercept	72·028	3·216	114·399	22·397	<0·001	<0·001
	Age at injury	-4·382	4·300	100·408	-1·019	0·311	
	Time since injury	21·327	3·207	222·061	6·650	<0·001	<0·001
	YEARDOI	-6·571	4·065	128·566	-1·617	0·108	
	Time since injury*YEARDOI	-3·587	5·691	227·545	-0·630	0·529	
AIS-D (n = 141)	Intercept	87·898	2·062	159·886	42·622	<0·001	<0·001
	Age at injury	-3·948	2·477	137·746	-1·594	0·113	
	Time since injury	9·003	2·258	262·082	3·988	<0·001	0·001
	YEARDOI	4·833	2·481	177·919	1·948	0·053	
	Time since injury*YEARDOI	-1·327	3·855	283·477	-0·344	0·731	
Female *tetraplegic* patients							
AIS-A (n = 159)	Intercept	24·237	4·524	163·686	5·357	<0·001	<0·001
	Age at injury	-9·850	6·779	156·749	-1·453	0·148	
	Time since injury	10·507	2·497	355·452	4·208	<0·001	0·001
	YEARDOI	5·133	5·905	169·137	0·869	0·386	
	Time since injury*YEARDOI	5·302	4·778	360·054	1·110	0·268	
AIS-B (n = 65)	Intercept	22·604	6·430	69·735	3·515	0·001	0·012
	Age at injury	3·610	10·180	59·798	0·355	0·724	
	Time since injury	15·631	5·356	147·111	2·918	0·004	0·065
	YEARDOI	-2·531	7·589	75·997	-0·334	0·740	
	Time since injury*YEARDOI	28·098	10·146	149·010	2·770	0·006	0·101
AIS-C (n = 137)	Intercept	46·119	4·521	156·625	10·201	<0·001	<0·001
	Age at injury	-8·147	5·699	129·568	-1·430	0·155	
	Time since injury	33·525	5·097	300·049	6·577	<0·001	<0·001
	YEARDOI	-5·592	5·552	190·080	-1·007	0·315	
	Time since injury*YEARDOI	7·493	8·904	313·693	0·841	0·401	
AIS-D (n = 223)	Intercept	78·811	3·385	244·159	23·284	<0·001	<0·001
	Age at injury	-12·694	4·050	226·736	-3·135	0·002	0·031
	Time since injury	20·743	3·138	411·772	6·610	<0·001	<0·001
	YEARDOI	6·613	3·432	267·809	1·927	0·055	
	Time since injury*YEARDOI	-4·149	4·902	424·187	-0·846	0·398	
Male *paraplegic* patients							
AIS-A (n = 904)	Intercept	51·981	0·972	914·106	53·458	<0·001	<0·001

	Age at injury	1·267	1·789	890·680	0·708	0·479	
	Time since injury	2·702	0·448	2004·930	6·031	<0·001	<0·001
	YEARDOI	1·654	1·315	937·993	1·258	0·209	
	Time since injury*YEARDOI	-0·092	0·812	2015·832	-0·113	0·910	
AIS-B (n = 209)	Intercept	55·948	2·662	219·897	21·021	<0·001	<0·001
	Age at injury	3·935	4·247	199·615	0·927	0·355	
	Time since injury	9·774	2·008	468·974	4·868	<0·001	<0·001
	YEARDOI	2·126	3·454	234·103	0·615	0·539	
	Time since injury*YEARDOI	8·730	3·624	475·405	2·409	0·016	0·262
AIS-C (n = 263)	Intercept	68·886	1·996	319·958	34·511	<0·001	<0·001
	Age at injury	-4·512	2·923	255·218	-1·544	0·124	
	Time since injury	16·287	2·202	609·466	7·395	<0·001	<0·001
	YEARDOI	-1·909	2·648	357·814	-0·721	0·472	
	Time since injury*YEARDOI	7·285	3·674	613·114	1·983	0·048	0·765
AIS-D (n = 358)	Intercept	91·186	1·201	380·477	75·924	<0·001	<0·001
	Age at injury	-6·661	1·792	324·152	-3·717	<0·001	0·004
	Time since injury	7·823	1·324	596·929	5·907	<0·001	<0·001
	YEARDOI	0·674	1·479	419·434	0·456	0·649	
	Time since injury*YEARDOI	1·703	2·238	635·148	0·761	0·447	
Male *tetraplegic* patients							
AIS-A (n = 634)	Intercept	13·366	2·057	651·350	6·499	<0·001	<0·001
	Age at injury	9·876	3·229	620·695	3·059	0·002	0·037
	Time since injury	10·190	1·270	1360·089	8·021	<0·001	<0·001
	YEARDOI	6·567	2·789	676·217	2·355	0·019	0·301
	Time since injury*YEARDOI	2·253	2·269	1373·221	0·993	0·321	
AIS-B (n = 228)	Intercept	21·932	3·592	270·608	6·106	<0·001	<0·001
	Age at injury	-8·972	5·698	233·317	-1·575	0·117	
	Time since injury	27·856	3·892	555·236	7·157	<0·001	<0·001
	YEARDOI	8·124	5·023	292·809	1·617	0·107	
	Time since injury*YEARDOI	0·400	7·056	563·636	0·057	0·955	
AIS-C (n = 410)	Intercept	48·482	2·940	512·615	16·490	<0·001	<0·001
	Age at injury	-17·742	3·927	436·909	-4·518	<0·001	<0·001
	Time since injury	32·623	3·256	958·356	10·019	<0·001	<0·001
	YEARDOI	-0·392	3·522	589·193	-0·111	0·911	
	Time since injury*YEARDOI	10·759	5·510	983·047	1·953	0·051	
AIS-D (n = 719)	Intercept	77·076	1·589	793·652	48·502	<0·001	<0·001
	Age at injury	-8·026	2·288	706·931	-3·509	0·001	0·008
	Time since injury	22·772	1·704	1460·152	13·363	<0·001	<0·001
	YEARDOI	3·847	1·891	902·888	2·035	0·042	0·675
	Time since injury*YEARDOI	-5·218	2·855	1500·083	-1·827	0·068	

[#]Time since injury measured in weeks: 2, 4, 8, 16, 26, and 52 weeks. DF: degree of freedom; YEARDOI: Year of injury

Additional File 2: Table S10. Model output of total light touch score (TLT) stratified by sex, plegia, and baseline AIS grades. Patients were enrolled in the EMSCI study. Significant values are highlighted in red.

Model	Variable	Estimate	Standard Error	DF	t-value	p-value	Adjusted p-value
Female *paraplegic* patients							
AIS-A (n = 227)	Intercept	69·955	3·136	230·224	22·306	<0·001	<0·001
	Age at injury	-3·702	4·870	223·963	-0·760	0·448	
	Time since injury[#]	1·237	1·249	462·063	0·990	0·323	
	YEARDOI	-2·224	4·157	233·422	-0·535	0·593	
	Time since injury*YEARDOI	2·919	2·205	463·938	1·324	0·186	
AIS-B (n = 75)	Intercept	96·260	4·217	75·975	22·827	<0·001	<0·001
	Age at injury	-13·361	6·849	73·087	-1·951	0·055	
	Time since injury	0·373	2·257	158·250	0·165	0·869	
	YEARDOI	-0·297	5·140	78·922	-0·058	0·954	
	Time since injury*YEARDOI	3·538	4·010	160·551	0·882	0·379	
AIS-C (n = 98)	Intercept	104·493	3·028	105·556	34·512	<0·001	<0·001
	Age at injury	-8·888	4·167	96·943	-2·133	0·035	0·567
	Time since injury	2·271	2·416	215·025	0·940	0·348	
	YEARDOI	-10·356	3·767	114·229	-2·749	0·007	0·111
	Time since injury*YEARDOI	4·457	4·299	218·947	1·037	0·301	
AIS-D (n = 139)	Intercept	106·810	2·901	151·448	36·823	<0·001	<0·001
	Age at injury	-7·049	3·574	139·253	-1·972	0·051	
	Time since injury	-2·676	2·326	244·675	-1·150	0·251	
	YEARDOI	-6·914	3·447	160·833	-2·006	0·047	0·745
	Time since injury*YEARDOI	11·072	4·088	260·407	2·708	0·007	0·116
Female *tetraplegic* patients							
AIS-A (n = 158)	Intercept	28·080	4·481	166·778	6·267	<0·001	<0·001
	Age at injury	3·063	6·668	152·658	0·459	0·647	
	Time since injury	2·089	3·582	355·178	0·583	0·560	
	YEARDOI	6·975	5·958	179·017	1·171	0·243	
	Time since injury*YEARDOI	19·352	6·865	365·393	2·819	0·005	0·081
AIS-B (n = 65)	Intercept	81·391	7·644	68·797	10·648	<0·001	<0·001
	Age at injury	-13·025	12·233	61·420	-1·065	0·291	
	Time since injury	0·283	5·552	145·666	0·051	0·960	
	YEARDOI	-20·543	8·975	73·378	-2·289	0·025	0·400
	Time since injury*YEARDOI	25·051	10·522	147·049	2·381	0·019	0·297
AIS-C (n = 137)	Intercept	76·576	5·279	135·733	14·507	<0·001	<0·001
	Age at injury	6·499	6·773	120·331	0·960	0·339	
	Time since injury	11·359	4·571	276·802	2·485	0·014	0·217
	YEARDOI	-7·765	6·358	157·756	-1·221	0·224	
	Time since injury*YEARDOI	-5·351	8·144	289·168	-0·657	0·512	
AIS-D (n = 223)	Intercept	87·440	4·679	243·732	18·690	<0·001	<0·001
	Age at injury	-1·676	5·670	227·731	-0·296	0·768	
	Time since injury	1·562	3·843	400·956	0·406	0·685	
	YEARDOI	6·067	4·693	261·451	1·293	0·197	
	Time since injury*YEARDOI	6·831	5·956	405·146	1·147	0·252	
Male *paraplegic* patients							
AIS-A (n = 905)	Intercept	65·319	1·604	915·220	40·717	<0·001	<0·001
	Age at injury	3·044	2·959	900·699	1·029	0·304	
	Time since injury	2·060	0·583	1997·424	3·536	<0·001	0·007

	YEARDOI	-1·668	2·162	930·229	-0·772	0·441	
	Time since injury*YEARDOI	0·520	1·056	2004·359	0·492	0·623	
AIS-B (n = 209)	Intercept	89·888	3·107	214·734	28·930	<0·001	<0·001
	Age at injury	7·225	5·025	206·007	1·438	0·152	
	Time since injury	0·933	1·569	462·215	0·595	0·552	
	YEARDOI	-8·382	3·997	220·995	-2·097	0·037	0·594
	Time since injury*YEARDOI	4·263	2·841	465·002	1·501	0·134	
AIS-C (n = 263)	Intercept	94·899	2·181	290·903	43·516	<0·001	<0·001
	Age at injury	-0·618	3·279	256·074	-0·188	0·851	
	Time since injury	-0·296	1·802	576·115	-0·164	0·870	
	YEARDOI	-0·432	2·859	311·650	-0·151	0·880	
	Time since injury*YEARDOI	8·698	3·015	576·816	2·884	0·004	0·065
AIS-D (n = 357)	Intercept	104·297	1·460	385·185	71·459	<0·001	<0·001
	Age at injury	-5·751	2·206	355·051	-2·607	0·010	0·152
	Time since injury	-0·534	1·264	589·102	-0·422	0·673	
	YEARDOI	-1·736	1·786	407·984	-0·972	0·331	
	Time since injury*YEARDOI	5·250	2·152	614·330	2·440	0·015	0·240
Male *tetraplegic* patients							
AIS-A (n = 635)	Intercept	25·099	2·081	694·332	12·061	<0·001	<0·001
	Age at injury	14·356	3·218	624·803	4·461	<0·001	<0·001
	Time since injury	12·858	1·848	1416·541	6·957	<0·001	<0·001
	YEARDOI	1·340	2·848	746·396	0·470	0·638	
	Time since injury*YEARDOI	-3·695	3·285	1442·218	-1·125	0·261	
AIS-B (n = 227)	Intercept	64·110	4·147	251·333	15·458	<0·001	<0·001
	Age at injury	1·945	6·745	227·128	0·288	0·773	
	Time since injury	9·178	3·866	537·357	2·374	0·018	0·287
	YEARDOI	-3·867	5·774	267·547	-0·670	0·504	
	Time since injury*YEARDOI	8·982	7·068	543·461	1·271	0·204	
AIS-C (n = 408)	Intercept	78·046	3·306	445·412	23·606	<0·001	<0·001
	Age at injury	-3·550	4·484	402·089	-0·792	0·429	
	Time since injury	4·755	2·951	894·801	1·611	0·108	
	YEARDOI	-2·907	3·907	489·389	-0·744	0·457	
	Time since injury*YEARDOI	12·953	4·999	912·972	2·591	0·010	0·156
AIS-D (n = 726)	Intercept	89·234	2·245	792·426	39·748	<0·001	<0·001
	Age at injury	4·728	3·256	728·895	1·452	0·147	
	Time since injury	4·206	2·093	1437·208	2·009	0·045	0·715
	YEARDOI	-1·826	2·657	868·189	-0·687	0·492	
	Time since injury*YEARDOI	-2·119	3·534	1476·752	-0·600	0·549	

[#]Time since injury measured in weeks: 2, 4, 8, 16, 26, and 52 weeks. DF: degree of freedom; YEARDOI: Year of injury

Additional File 2: Table S11. Model output of total pinprick score (TPP) stratified by sex, plegia, and baseline AIS grades. Patients were enrolled in the EMSCI study. Significant values are highlighted in red.

Model	Variable	Estimate	Standard Error	DF	t-value	p-value	Adjusted p-value
Female *paraplegic* patients							
AIS-A (n = 228)	Intercept	70·107	3·044	230·808	23·029	<0·001	<0·001
	Age at injury	-5·570	4·730	225·108	-1·177	0·240	
	Time since injury#	-0·634	1·156	459·807	-0·548	0·584	
	YEARDOI	-4·393	4·032	233·755	-1·090	0·277	
	Time since injury*YEARDOI	3·971	2·044	461·577	1·943	0·053	
AIS-B (n = 75)	Intercept	94·957	5·836	74·147	16·270	<0·001	<0·001
	Age at injury	-21·434	9·502	72·031	-2·256	0·027	0·434
	Time since injury	-0·801	2·715	156·389	-0·295	0·769	
	YEARDOI	-8·598	7·097	76·301	-1·212	0·230	
	Time since injury*YEARDOI	9·075	4·828	158·174	1·880	0·062	
AIS-C (n = 98)	Intercept	106·434	3·882	100·327	27·417	<0·001	<0·001
	Age at injury	-13·561	5·361	93·321	-2·530	0·013	0·210
	Time since injury	2·837	2·829	208·995	1·003	0·317	
	YEARDOI	-19·354	4·812	107·110	-4·022	<0·001	0·002
	Time since injury*YEARDOI	2·426	5·033	212·366	0·482	0·630	
AIS-D (n = 139)	Intercept	111·806	4·083	148·785	27·386	<0·001	<0·001
	Age at injury	-19·606	5·063	140·041	-3·872	<0·001	0·003
	Time since injury	-2·574	2·778	238·560	-0·926	0·355	
	YEARDOI	-11·903	4·829	155·469	-2·465	0·015	0·237
	Time since injury*YEARDOI	10·747	4·909	250·495	2·189	0·030	0·472
Female *tetraplegic* patients							
AIS-A (n = 158)	Intercept	28·928	4·189	163·074	6·905	<0·001	<0·001
	Age at injury	-4·506	6·240	149·757	-0·722	0·471	
	Time since injury	-0·654	3·286	351·050	-0·199	0·843	
	YEARDOI	1·654	5·566	174·637	0·297	0·767	
	Time since injury*YEARDOI	14·134	6·317	361·503	2·238	0·026	0·414
AIS-B (n = 65)	Intercept	58·907	9·130	72·072	6·452	<0·001	<0·001
	Age at injury	-6·728	14·549	63·463	-0·462	0·645	
	Time since injury	9·659	7·087	148·503	1·363	0·175	
	YEARDOI	-17·125	10·746	77·505	-1·594	0·115	
	Time since injury*YEARDOI	3·485	13·426	150·045	0·260	0·796	
AIS-C (n = 136)	Intercept	66·184	7·399	137·600	8·945	<0·001	<0·001
	Age at injury	4·683	9·568	126·369	0·490	0·625	
	Time since injury	13·690	5·668	272·242	2·415	0·016	0·262
	YEARDOI	-17·565	8·863	153·664	-1·982	0·049	0·789
	Time since injury*YEARDOI	-0·016	10·189	278·504	-0·002	0·999	
AIS-D (n = 223)	Intercept	87·090	6·543	240·843	13·311	<0·001	<0·001
	Age at injury	-8·144	7·949	227·240	-1·025	0·307	
	Time since injury	1·557	5·030	394·374	0·310	0·757	
	YEARDOI	-4·589	6·547	256·214	-0·701	0·484	
	Time since injury*YEARDOI	12·019	7·844	398·688	1·532	0·126	
Male *paraplegic* patients							
AIS-A (n = 905)	Intercept	65·207	1·574	916·149	41·438	<0·001	<0·001
	Age at injury	0·409	2·901	900·033	0·141	0·888	

	Time since injury	0·346	0·601	1997·015	0·575	0·565	
	YEARDOI	-3·455	2·121	933·058	-1·629	0·104	
	Time since injury*YEARDOI	2·577	1·090	2004·857	2·364	0·018	0·291
AIS-B (n = 209)	Intercept	88·798	3·783	215·845	23·474	<0·001	<0·001
	Age at injury	-1·584	6·112	206·220	-0·259	0·796	
	Time since injury	-0·734	2·016	464·329	-0·364	0·716	
	YEARDOI	-14·204	4·871	222·959	-2·916	0·004	0·063
	Time since injury*YEARDOI	7·060	3·651	467·412	1·934	0·054	
AIS-C (n = 261)	Intercept	96·431	2·910	274·841	33·135	<0·001	<0·001
	Age at injury	-9·261	4·417	249·723	-2·097	0·037	0·592
	Time since injury	-1·657	2·045	561·984	-0·810	0·418	
	YEARDOI	-7·661	3·796	288·638	-2·018	0·045	0·713
	Time since injury*YEARDOI	12·292	3·420	562·375	3·594	<0·001	0·006
AIS-D (n = 356)	Intercept	108·899	2·232	382·398	48·791	<0·001	<0·001
	Age at injury	-19·067	3·380	356·415	-5·641	<0·001	<0·001
	Time since injury	-3·121	1·804	583·176	-1·730	0·084	
	YEARDOI	-7·993	2·729	401·957	-2·929	0·004	0·057
	Time since injury*YEARDOI	11·769	3·078	605·874	3·824	<0·001	0·002
Male *tetraplegic* patients							
AIS-A (n = 634)	Intercept	23·078	1·756	668·981	13·141	<0·001	<0·001
	Age at injury	7·975	2·721	601·383	2·931	0·004	0·056
	Time since injury	6·796	1·549	1389·822	4·388	<0·001	<0·001
	YEARDOI	-0·877	2·409	718·872	-0·364	0·716	
	Time since injury*YEARDOI	-3·363	2·758	1415·535	-1·219	0·223	
AIS-B (n = 227)	Intercept	49·829	4·588	255·590	10·862	<0·001	<0·001
	Age at injury	-10·879	7·447	229·099	-1·461	0·145	
	Time since injury	8·468	4·452	539·512	1·902	0·058	
	YEARDOI	-13·074	6·391	272·841	-2·046	0·042	0·668
	Time since injury*YEARDOI	13·153	8·138	546·100	1·616	0·107	
AIS-C (n = 408)	Intercept	75·235	4·374	443·042	17·201	<0·001	<0·001
	Age at injury	-16·497	5·957	404·954	-2·769	0·006	0·094
	Time since injury	1·831	3·683	892·040	0·497	0·619	
	YEARDOI	-16·518	5·164	481·680	-3·199	0·002	0·024
	Time since injury*YEARDOI	21·719	6·245	909·217	3·478	0·001	0·009
AIS-D (n = 725)	Intercept	90·626	3·084	785·058	29·391	<0·001	<0·001
	Age at injury	-3·940	4·486	730·992	-0·878	0·380	
	Time since injury	4·469	2·718	1420·517	1·645	0·100	
	YEARDOI	-14·446	3·644	851·689	-3·965	<0·001	0·001
	Time since injury*YEARDOI	3·028	4·595	1457·247	0·659	0·510	

[#]Time since injury measured in weeks: 2, 4, 8, 16, 26, and 52 weeks. DF: degree of freedom; YEARDOI: Year of injury

Additional File 2: Table S12. Model output of total sensory score (TSS) stratified by sex, plegia, and baseline AIS grades. Patients were enrolled in the EMSCI study. Significant values are highlighted in red.

Model	Variable	Estimate	Standard Error	DF	t-value	p-value	Adjusted p-value
Female *paraplegic* patients							
AIS-A (n = 227)	Intercept	104·984	4·714	230·157	22·269	<0·001	<0·001
	Age at injury	-5·636	7·320	223·848	-0·770	0·442	
	Time since injury[#]	1·563	1·884	461·990	0·829	0·407	
	YEARDOI	-3·186	6·249	233·378	-0·510	0·611	
	Time since injury*YEARDOI	4·717	3·327	463·879	1·418	0·157	
AIS-B (n = 75)	Intercept	145·222	6·282	75·891	23·117	<0·001	<0·001
	Age at injury	-21·204	10·199	72·888	-2·079	0·041	0·658
	Time since injury	0·440	3·425	158·190	0·128	0·898	
	YEARDOI	-1·546	7·660	78·958	-0·202	0·841	
	Time since injury*YEARDOI	6·030	6·083	160·575	0·991	0·323	
AIS-C (n = 98)	Intercept	156·151	4·549	106·350	34·326	<0·001	<0·001
	Age at injury	-13·171	6·255	97·400	-2·106	0·038	0·605
	Time since injury	3·775	3·689	215·756	1·023	0·307	
	YEARDOI	-14·351	5·664	115·370	-2·534	0·013	0·202
	Time since injury*YEARDOI	5·997	6·562	219·777	0·914	0·362	
AIS-D (n = 139)	Intercept	161·170	4·315	151·217	37·355	<0·001	<0·001
	Age at injury	-11·561	5·314	138·885	-2·175	0·031	0·501
	Time since injury	-4·043	3·481	244·629	-1·162	0·247	
	YEARDOI	-10·865	5·128	160·712	-2·119	0·036	0·571
	Time since injury*YEARDOI	16·978	6·115	260·535	2·776	0·006	0·094
Female *tetraplegic* patients							
AIS-A (n = 158)	Intercept	42·009	6·715	166·460	6·256	<0·001	<0·001
	Age at injury	4·303	9·987	151·958	0·431	0·667	
	Time since injury	2·999	5·435	354·930	0·552	0·582	
	YEARDOI	10·376	8·933	179·033	1·162	0·247	
	Time since injury*YEARDOI	29·787	10·416	365·379	2·860	0·005	0·072
AIS-B (n = 65)	Intercept	122·788	11·398	69·103	10·773	<0·001	<0·001
	Age at injury	-21·780	18·228	61·523	-1·195	0·237	
	Time since injury	1·975	8·385	145·935	0·236	0·814	
	YEARDOI	-30·127	13·390	73·827	-2·250	0·027	0·439
	Time since injury*YEARDOI	33·204	15·890	147·351	2·090	0·038	0·614
AIS-C (n = 137)	Intercept	114·748	7·961	135·879	14·415	<0·001	<0·001
	Age at injury	9·640	10·215	120·516	0·944	0·347	
	Time since injury	16·911	6·883	276·908	2·457	0·015	0·234
	YEARDOI	-11·606	9·588	157·855	-1·211	0·228	
	Time since injury*YEARDOI	-6·874	12·265	289·242	-0·560	0·576	
AIS-D (n = 223)	Intercept	129·974	7·112	244·650	18·275	<0·001	<0·001
	Age at injury	-1·316	8·616	228·250	-0·153	0·879	
	Time since injury	2·745	5·903	402·135	0·465	0·642	
	YEARDOI	9·384	7·136	262·768	1·315	0·190	
	Time since injury*YEARDOI	9·930	9·148	406·393	1·086	0·278	
Male *paraplegic* patients							
AIS-A (n = 905)	Intercept	98·079	2·402	915·296	40·828	<0·001	<0·001
	Age at injury	4·412	4·430	900·540	0·996	0·320	
	Time since injury	3·052	0·879	1997·565	3·471	0·001	0·009

	YEARDOI	-2·629	3·237	930·541	-0·812	0·417	
	Time since injury*YEARDOI	1·081	1·594	2004·606	0·678	0·498	
AIS-B (n = 209)	Intercept	135·035	4·637	215·010	29·124	<0·001	<0·001
	Age at injury	10·575	7·495	205·869	1·411	0·160	
	Time since injury	1·009	2·393	462·463	0·422	0·674	
	YEARDOI	-12·817	5·967	221·563	-2·148	0·033	0·525
	Time since injury*YEARDOI	6·971	4·333	465·383	1·609	0·108	
AIS-C (n = 263)	Intercept	142·476	3·306	292·393	43·096	<0·001	<0·001
	Age at injury	-1·532	4·965	256·101	-0·309	0·758	
	Time since injury	0·089	2·785	577·557	0·032	0·975	
	YEARDOI	-0·647	4·337	314·061	-0·149	0·882	
	Time since injury*YEARDOI	12·542	4·661	578·352	2·691	0·007	0·117
AIS-D (n = 357)	Intercept	156·131	2·225	386·959	70·171	<0·001	<0·001
	Age at injury	-8·597	3·362	356·670	-2·557	0·011	0·176
	Time since injury	-1·047	1·930	590·795	-0·543	0·588	
	YEARDOI	-2·241	2·722	409·851	-0·823	0·411	
	Time since injury*YEARDOI	8·235	3·286	616·011	2·507	0·012	0·199
Male *tetraplegic* patients							
AIS-A (n = 635)	Intercept	37·636	3·120	693·511	12·062	<0·001	<0·001
	Age at injury	21·655	4·825	624·255	4·488	<0·001	<0·001
	Time since injury	19·344	2·767	1415·768	6·991	<0·001	<0·001
	YEARDOI	1·768	4·270	745·386	0·414	0·679	
	Time since injury*YEARDOI	-5·496	4·919	1441·394	-1·117	0·264	
AIS-B (n = 227)	Intercept	96·411	6·252	250·880	15·421	<0·001	<0·001
	Age at injury	2·433	10·165	226·524	0·239	0·811	
	Time since injury	13·400	5·847	536·979	2·292	0·022	0·357
	YEARDOI	-6·259	8·705	267·190	-0·719	0·473	
	Time since injury*YEARDOI	14·190	10·688	543·135	1·328	0·185	
AIS-C (n = 408)	Intercept	117·362	4·992	446·058	23·511	<0·001	<0·001
	Age at injury	-5·403	6·769	402·470	-0·798	0·425	
	Time since injury	6·667	4·466	895·472	1·493	0·136	
	YEARDOI	-4·553	5·899	490·293	-0·772	0·441	
	Time since injury*YEARDOI	19·882	7·565	913·705	2·628	0·009	0·140
AIS-D (n = 726)	Intercept	133·639	3·382	792·125	39·521	<0·001	<0·001
	Age at injury	7·114	4·904	728·339	1·451	0·147	
	Time since injury	6·996	3·158	1437·164	2·215	0·027	0·430
	YEARDOI	-2·726	4·002	868·166	-0·681	0·496	
	Time since injury*YEARDOI	-4·003	5·332	1476·837	-0·751	0·453	

[#]Time since injury measured in weeks: 2, 4, 8, 16, 26, and 52 weeks. DF: degree of freedom; YEARDOI: Year of injury

Additional File 2: Table S13. Model output of SCIM Total Score stratified by sex, plegia, and baseline AIS grades. Patients were enrolled in the EMSCI study. Significant values are highlighted in red.

Model	Variable	Estimate	Standard Error	DF	t-value	p-value	Adjusted p-value
Female *paraplegic* patients							
AIS-A (n = 218)	Intercept	43·926	2·416	334·065	18·183	<0·001	<0·001
	Age at injury	-27·472	3·315	208·734	-8·287	<0·001	<0·001
	Time since injury[#]	37·196	3·924	523·801	9·480	<0·001	<0·001
	YEARDOI	-4·603	3·397	392·737	-1·355	0·176	
	Time since injury*YEARDOI	4·364	6·755	539·907	0·646	0·519	
AIS-B (n = 73)	Intercept	51·365	4·477	87·835	11·474	<0·001	<0·001
	Age at injury	-24·793	6·799	59·261	-3·647	0·001	0·009
	Time since injury	52·274	6·494	167·259	8·050	<0·001	<0·001
	YEARDOI	-10·289	5·794	115·387	-1·776	0·078	
	Time since injury*YEARDOI	-16·821	11·250	175·186	-1·495	0·137	
AIS-C (n = 98)	Intercept	65·041	4·700	135·155	13·838	<0·001	<0·001
	Age at injury	-45·344	6·013	103·245	-7·541	<0·001	<0·001
	Time since injury	54·288	6·511	228·207	8·338	<0·001	<0·001
	YEARDOI	-8·910	6·090	161·692	-1·463	0·145	
	Time since injury*YEARDOI	-22·903	11·313	235·084	-2·025	0·044	0·705
AIS-D (n = 138)	Intercept	81·892	5·554	148·531	14·746	<0·001	<0·001
	Age at injury	-43·583	6·629	111·640	-6·575	<0·001	<0·001
	Time since injury	39·923	7·341	238·717	5·439	<0·001	<0·001
	YEARDOI	-10·058	6·828	177·867	-1·473	0·143	
	Time since injury*YEARDOI	5·189	12·425	267·760	0·418	0·677	
Female *tetraplegic* patients							
AIS-A (n = 157)	Intercept	19·975	2·986	180·346	6·690	<0·001	<0·001
	Age at injury	-12·238	4·425	158·999	-2·765	0·006	0·102
	Time since injury	17·468	2·798	355·448	6·242	<0·001	<0·001
	YEARDOI	-4·408	3·977	199·952	-1·108	0·269	
	Time since injury*YEARDOI	8·243	5·280	366·791	1·561	0·119	
AIS-B (n = 63)	Intercept	19·984	5·158	67·259	3·875	<0·001	0·004
	Age at injury	-13·081	7·918	55·742	-1·652	0·104	
	Time since injury	29·805	5·116	134·704	5·826	<0·001	<0·001
	YEARDOI	-1·259	6·049	79·016	-0·208	0·836	
	Time since injury*YEARDOI	6·720	9·561	137·994	0·703	0·483	
AIS-C (n = 131)	Intercept	37·386	4·557	158·771	8·204	<0·001	<0·001
	Age at injury	-29·434	5·597	128·050	-5·259	<0·001	<0·001
	Time since injury	40·987	5·528	295·786	7·415	<0·001	<0·001
	YEARDOI	-7·249	5·602	201·004	-1·294	0·197	
	Time since injury*YEARDOI	4·311	9·552	307·447	0·451	0·652	
AIS-D (n = 213)	Intercept	75·167	6·247	238·785	12·033	<0·001	<0·001
	Age at injury	-49·424	7·368	211·191	-6·708	<0·001	<0·001
	Time since injury	50·630	7·436	388·540	6·809	<0·001	<0·001
	YEARDOI	-5·318	6·498	287·507	-0·818	0·414	
	Time since injury*YEARDOI	-2·109	11·569	400·814	-0·182	0·856	
Male *paraplegic* patients							
AIS-A (n = 868)	Intercept	45·763	1·235	1282·290	37·046	<0·001	<0·001
	Age at injury	-23·562	2·050	836·493	-11·491	<0·001	<0·001
	Time since injury	41·920	2·026	2238·604	20·688	<0·001	<0·001

	YEARDOI	-8·434	1·779	1595·075	-4·742	<0·001	<0·001
	Time since injury*YEARDOI	0·416	3·557	2288·418	0·117	0·907	
AIS-B (n = 207)	Intercept	49·102	3·065	267·171	16·020	<0·001	<0·001
	Age at injury	-25·463	4·550	182·813	-5·596	<0·001	<0·001
	Time since injury	42·770	4·303	488·769	9·939	<0·001	<0·001
	YEARDOI	-2·317	4·119	325·018	-0·563	0·574	
	Time since injury*YEARDOI	5·837	7·631	504·804	0·765	0·445	
AIS-C (n = 253)	Intercept	59·116	3·091	357·185	19·123	<0·001	<0·001
	Age at injury	-31·165	4·182	229·926	-7·452	<0·001	<0·001
	Time since injury	41·916	4·721	592·955	8·879	<0·001	<0·001
	YEARDOI	-6·327	4·110	420·569	-1·539	0·125	
	Time since injury*YEARDOI	7·221	7·721	595·525	0·935	0·350	
AIS-D (n = 344)	Intercept	81·470	3·029	430·436	26·900	<0·001	<0·001
	Age at injury	-33·315	4·259	301·227	-7·823	<0·001	<0·001
	Time since injury	35·237	4·562	638·317	7·725	<0·001	<0·001
	YEARDOI	-16·957	3·785	499·663	-4·480	<0·001	<0·001
	Time since injury*YEARDOI	13·783	7·563	674·742	1·822	0·069	
Male *tetraplegic* patients							
AIS-A (n = 621)	Intercept	16·430	1·476	731·391	11·129	<0·001	<0·001
	Age at injury	-10·757	2·257	612·141	-4·766	<0·001	<0·001
	Time since injury	26·805	1·656	1390·628	16·188	<0·001	<0·001
	YEARDOI	-0·332	2·049	812·747	-0·162	0·871	
	Time since injury*YEARDOI	-2·304	2·919	1418·310	-0·789	0·430	
AIS-B (n = 221)	Intercept	20·223	2·809	304·907	7·199	<0·001	<0·001
	Age at injury	-18·635	4·240	240·905	-4·395	<0·001	<0·001
	Time since injury	36·750	3·887	549·905	9·456	<0·001	<0·001
	YEARDOI	1·697	3·931	343·554	0·432	0·666	
	Time since injury*YEARDOI	1·442	6·881	560·151	0·210	0·834	
AIS-C (n = 394)	Intercept	42·876	2·835	514·055	15·122	<0·001	<0·001
	Age at injury	-40·378	3·707	414·701	-10·894	<0·001	<0·001
	Time since injury	45·859	3·552	934·257	12·913	<0·001	<0·001
	YEARDOI	-6·950	3·414	613·321	-2·036	0·042	0·675
	Time since injury*YEARDOI	-2·292	5·886	961·985	-0·389	0·697	
AIS-D (n = 700)	Intercept	67·545	2·851	834·689	23·695	<0·001	<0·001
	Age at injury	-39·461	4·046	675·970	-9·754	<0·001	<0·001
	Time since injury	52·590	3·771	1451·561	13·945	<0·001	<0·001
	YEARDOI	-7·248	3·465	994·668	-2·092	0·037	0·587
	Time since injury*YEARDOI	4·523	6·301	1502·528	0·718	0·473	

[#]Time since injury measured in weeks: 2, 4, 8, 16, 26, and 52 weeks. DF: degree of freedom; YEARDOI: Year of injury

Additional File 2: Table S14. Model output of Walking Index for Spinal Cord Injury (WISCI), stratified by sex, plegia, and baseline AIS grades/ Patients were enrolled in the EMSCI study. Significant values are highlighted in red.

Model	Variable	Estimate	Standard Error	DF	t-value	p-value	Adjusted p-value
Female *paraplegic* patients							
AIS-A (n = 224)	Intercept	0·438	0·612	282·626	0·716	0·475	
	Age at injury	-1·688	0·908	226·895	-1·860	0·064	
	Time since injury[#]	1·101	0·682	491·553	1·615	0·107	
	YEARDOI	1·047	0·833	312·012	1·257	0·210	
	Time since injury*YEARDOI	2·029	1·197	503·464	1·695	0·091	
AIS-B (n = 75)	Intercept	3·709	1·499	78·784	2·474	0·016	0·248
	Age at injury	-3·488	2·336	65·307	-1·493	0·140	
	Time since injury	11·678	1·513	156·986	7·717	<0·001	<0·001
	YEARDOI	-1·417	1·873	90·985	-0·757	0·451	
	Time since injury*YEARDOI	-7·949	2·665	163·262	-2·983	0·003	0·053
AIS-C (n = 96)	Intercept	6·933	1·354	131·998	5·120	<0·001	<0·001
	Age at injury	-8·078	1·749	100·897	-4·619	<0·001	<0·001
	Time since injury	16·718	1·820	225·109	9·184	<0·001	<0·001
	YEARDOI	-2·041	1·735	152·774	-1·176	0·241	
	Time since injury*YEARDOI	-9·873	3·155	228·705	-3·129	0·002	0·032
AIS-D (n = 133)	Intercept	13·107	1·875	150·006	6·991	<0·001	<0·001
	Age at injury	-10·209	2·253	125·648	-4·531	<0·001	<0·001
	Time since injury	10·884	2·199	223·173	4·949	<0·001	<0·001
	YEARDOI	-0·466	2·249	171·461	-0·207	0·836	
	Time since injury*YEARDOI	0·299	3·770	250·966	0·079	0·937	
Female *tetraplegic* patients							
AIS-A (n = 157)	Intercept	0·003	0·405	227·823	0·007	0·994	
	Age at injury	0·391	0·574	177·498	0·681	0·497	
	Time since injury	1·316	0·535	400·836	2·461	0·014	0·229
	YEARDOI	-0·259	0·553	272·743	-0·469	0·640	
	Time since injury*YEARDOI	-0·859	1·001	415·208	-0·859	0·391	
AIS-B (n = 64)	Intercept	0·900	1·015	75·404	0·887	0·378	
	Age at injury	-1·192	1·493	50·664	-0·798	0·429	
	Time since injury	0·654	1·372	144·230	0·476	0·635	
	YEARDOI	-0·877	1·228	95·417	-0·714	0·477	
	Time since injury*YEARDOI	9·228	2·591	153·028	3·561	0·001	0·008
AIS-C (n = 130)	Intercept	4·202	1·136	172·374	3·699	<0·001	0·005
	Age at injury	-5·568	1·411	131·721	-3·947	<0·001	0·002
	Time since injury	9·120	1·474	313·378	6·188	<0·001	<0·001
	YEARDOI	-0·418	1·424	217·286	-0·294	0·769	
	Time since injury*YEARDOI	-1·614	2·596	328·236	-0·622	0·535	
AIS-D (n = 211)	Intercept	13·541	1·660	240·461	8·158	<0·001	<0·001
	Age at injury	-11·255	1·956	207·845	-5·753	<0·001	<0·001
	Time since injury	13·846	2·125	403·448	6·516	<0·001	<0·001
	YEARDOI	-0·298	1·722	292·405	-0·173	0·863	
	Time since injury*YEARDOI	-1·374	3·249	412·907	-0·423	0·673	
Male *paraplegic* patients							
AIS-A (n = 888)	Intercept	0·549	0·310	1013·756	1·771	0·077	
	Age at injury	-0·843	0·554	870·069	-1·522	0·129	

	Time since injury	1·814	0·329	2039·791	5·518	<0·001	<0·001
	YEARDOI	0·601	0·430	1137·997	1·396	0·163	
	Time since injury*YEARDOI	0·388	0·587	2076·012	0·660	0·509	
AIS-B (n = 204)	Intercept	1·645	0·992	227·489	1·658	0·099	
	Age at injury	-1·272	1·568	189·436	-0·811	0·418	
	Time since injury	7·455	1·044	448·830	7·140	<0·001	<0·001
	YEARDOI	0·517	1·309	257·882	0·395	0·693	
	Time since injury*YEARDOI	0·344	1·888	459·143	0·182	0·855	
AIS-C (n = 261)	Intercept	5·639	0·911	354·588	6·189	<0·001	<0·001
	Age at injury	-6·709	1·273	244·994	-5·271	<0·001	<0·001
	Time since injury	11·815	1·293	596·877	9·135	<0·001	<0·001
	YEARDOI	-1·444	1·224	409·019	-1·180	0·239	
	Time since injury*YEARDOI	-0·049	2·155	601·303	-0·023	0·982	
AIS-D (n = 344)	Intercept	14·263	0·993	395·962	14·363	<0·001	<0·001
	Age at injury	-8·054	1·436	299·581	-5·610	<0·001	<0·001
	Time since injury	10·480	1·349	586·255	7·770	<0·001	<0·001
	YEARDOI	-4·277	1·237	461·493	-3·457	0·001	0·010
	Time since injury*YEARDOI	2·957	2·257	632·922	1·310	0·191	
Male *tetraplegic* patients							
AIS-A (n = 630)	Intercept	0·064	0·180	843·799	0·354	0·723	
	Age at injury	0·113	0·265	650·389	0·425	0·671	
	Time since injury	0·228	0·245	1523·835	0·930	0·353	
	YEARDOI	-0·144	0·251	980·587	-0·572	0·567	
	Time since injury*YEARDOI	1·111	0·428	1561·915	2·596	0·010	0·152
AIS-B (n = 222)	Intercept	0·479	0·590	363·764	0·812	0·417	
	Age at injury	-1·336	0·881	273·001	-1·516	0·131	
	Time since injury	5·361	0·912	606·060	5·877	<0·001	<0·001
	YEARDOI	-0·144	0·836	419·166	-0·172	0·863	
	Time since injury*YEARDOI	-2·432	1·628	618·280	-1·494	0·136	
AIS-C (n = 402)	Intercept	5·473	0·763	551·423	7·171	<0·001	<0·001
	Age at injury	-7·094	0·996	428·811	-7·123	<0·001	<0·001
	Time since injury	9·993	1·012	971·644	9·874	<0·001	<0·001
	YEARDOI	-1·181	0·928	662·465	-1·273	0·203	
	Time since injury*YEARDOI	0·259	1·687	1001·517	0·154	0·878	
AIS-D (n = 692)	Intercept	11·959	0·786	796·677	15·220	<0·001	<0·001
	Age at injury	-7·210	1·119	643·657	-6·445	<0·001	<0·001
	Time since injury	12·762	1·057	1432·559	12·071	<0·001	<0·001
	YEARDOI	-0·852	0·954	952·457	-0·893	0·372	
	Time since injury*YEARDOI	0·243	1·772	1492·899	0·137	0·891	

[#]Time since injury measured in weeks: 2, 4, 8, 16, 26, and 52 weeks. DF: degree of freedom; YEARDOI: Year of injury

Additional File 2: Table S15. Model output of 6-minute walking test (6-MWT) stratified by sex, plegia, and baseline AIS grades. Patients were enrolled in the EMSCI study. Significant values are highlighted in red.
Note: The model was only run for patients with AIS-C and D injuries. Male and female patients were pooled due to low sample numbers.

Model	Variable	Estimate	Standard Error	DF	t-value	p-value	Adjusted p-value
Paraplegic patients							
AIS-C (n = 165)	Intercept	206·190	39·006	243·162	5·286	<0·001	<0·001
	Age at injury	-254·326	59·871	159·436	-4·248	<0·001	<0·001
	Time since injury[#]	219·369	39·325	161·359	5·578	<0·001	<0·001
	YEARDOI	64·760	58·892	269·890	1·100	0·273	
	Time since injury*YEARDOI	-88·621	74·719	180·227	-1·186	0·237	
AIS-D (n = 332)	Intercept	415·658	27·028	407·472	15·379	<0·001	<0·001
	Age at injury	-280·959	41·636	330·604	-6·748	<0·001	<0·001
	Time since injury	179·867	26·249	380·062	6·852	<0·001	<0·001
	YEARDOI	-13·864	34·050	490·834	-0·407	0·684	
	Time since injury*YEARDOI	-11·944	46·635	435·311	-0·256	0·798	
Tetraplegic patients							
AIS-C (n = 169)	Intercept	309·594	41·125	239·278	7·528	<0·001	<0·001
	Age at injury	-264·405	56·602	164·004	-4·671	<0·001	<0·001
	Time since injury	121·314	41·086	146·590	2·953	0·004	0·015
	YEARDOI	-78·440	59·862	265·375	-1·310	0·191	
	Time since injury*YEARDOI	39·772	75·195	171·270	0·529	0·598	
AIS-D (n = 561)	Intercept	435·224	22·906	662·864	19·000	<0·001	<0·001
	Age at injury	-204·339	32·399	534·487	-6·307	<0·001	<0·001
	Time since injury	128·817	20·737	697·595	6·212	<0·001	<0·001
	YEARDOI	-57·637	27·464	813·681	-2·099	0·036	0·145
	Time since injury*YEARDOI	7·957	35·638	748·220	0·223	0·823	

[#]Time since injury measured in weeks: 2, 4, 8, 16, 26, and 52 weeks. DF: degree of freedom; YEARDOI: Year of injury

Additional File 2: Table S16. Model output of 10m walking test (10-MWT) stratified by sex, plegia, and baseline AIS grades. Patients were enrolled in the EMSCI study. Significant values are highlighted in red.
Note: The model was only run for patients with AIS-C and D injuries.

Model	Variable	Estimate	Standard Error	DF	t-value	p-value	Adjusted p-value
Female *paraplegic* patients							
AIS-C (n = 48)	Intercept	371·520	76·860	72·107	4·834	<0·001	<0·001
	Age at injury	-187·293	102·848	54·588	-1·821	0·074	
	Time since injury	213·307	110·335	73·342	1·933	0·057	
	YEARDOI	53·834	128·403	79·131	0·419	0·676	
	Time since injury*YEARDOI	-494·931	210·995	78·922	-2·346	0·022	0·172
AIS-D (n = 94)	Intercept	333·702	74·782	132·269	4·462	<0·001	<0·001
	Age at injury	-97·158	90·440	94·713	-1·074	0·285	
	Time since injury	102·081	85·856	125·585	1·189	0·237	
	YEARDOI	44·442	92·793	154·658	0·479	0·633	
	Time since injury*YEARDOI	-95·611	143·548	144·338	-0·666	0·506	
Female *tetraplegic* patients							
AIS-C (n = 43)	Intercept	356·847	100·504	63·304	3·551	0·001	0·006
	Age at injury	-110·666	102·884	28·668	-1·076	0·291	
	Time since injury	68·553	128·489	52·524	0·534	0·596	
	YEARDOI	45·799	166·219	67·509	0·276	0·784	
	Time since injury*YEARDOI	-154·041	238·379	54·197	-0·646	0·521	
AIS-D (n = 150)	Intercept	251·719	61·915	219·265	4·066	<0·001	0·001
	Age at injury	3·385	66·007	149·880	0·051	0·959	
	Time since injury	204·301	87·079	266·064	2·346	0·020	0·158
	YEARDOI	125·138	73·092	252·262	1·712	0·088	
	Time since injury*YEARDOI	-258·253	133·839	269·564	-1·930	0·055	
Male *paraplegic* patients							
AIS-C (n = 163)	Intercept	355·202	56·846	252·671	6·249	<0·001	<0·001
	Age at injury	-48·457	65·580	136·991	-0·739	0·461	
	Time since injury	-97·954	79·230	251·152	-1·236	0·218	
	YEARDOI	-43·517	86·680	270·759	-0·502	0·616	
	Time since injury*YEARDOI	155·030	134·018	258·543	1·157	0·248	
AIS-D (n = 285)	Intercept	371·284	33·781	401·548	10·991	<0·001	<0·001
	Age at injury	-165·105	50·038	286·462	-3·300	0·001	0·009
	Time since injury	189·044	49·271	438·411	3·837	<0·001	0·001
	YEARDOI	33·539	45·734	468·970	0·733	0·464	
	Time since injury*YEARDOI	-115·229	82·825	477·317	-1·391	0·165	
Male *tetraplegic* patients							
AIS-C (n = 169)	Intercept	306·170	54·260	269·747	5·643	<0·001	<0·001
	Age at injury	-60·940	58·517	153·925	-1·041	0·299	
	Time since injury	109·022	75·078	239·126	1·452	0·148	
	YEARDOI	105·419	82·125	286·126	1·284	0·200	
	Time since injury*YEARDOI	-157·162	129·562	256·560	-1·213	0·226	
AIS-D (n = 547)	Intercept	412·878	26·887	752·735	15·356	<0·001	<0·001
	Age at injury	-108·114	35·787	500·205	-3·021	0·003	0·021
	Time since injury	71·579	35·699	914·407	2·005	0·045	0·362

YEARDOI	-28·628	34·420	919·479	-0·832	0·406
Time since injury*YEARDOI	-19·204	59·742	967·457	-0·321	0·748

[#]Time since injury measured in weeks: 2, 4, 8, 16, 26, and 52 weeks. DF: degree of freedom; YEARDOI: Year of injury

Additional File 2: Table S17. Sensitivity Analysis II: Sex. Model output of total motor score, SCIM2 and 3, and WISCI stratified by plegia, and baseline AIS grades. Patients were enrolled in the EMSCI study. Significant values are highlighted in red.

Model	Variable	Estimate	Standard Error	DF	t-value	p-value	Adjusted p-value
**Total Motor Score**							
Paraplegia							
AIS-A (n = 1132)	Intercept	52·503	0·882	1124·158	59·505	0·000	0·000
	Age	0·452	1·495	1118·578	0·302	0·763	
	Time since injury[#]	2·503	0·190	2487·113	13·168	0·000	0·000
	Sex	0·712	0·734	1118·619	0·970	0·332	
AIS-B (n = 285)	Intercept	60·779	2·161	279·556	28·125	0·000	0·000
	Age	-0·115	3·698	273·888	-0·031	0·975	
	Time since injury	13·609	0·774	634·241	17·579	0·000	0·000
	Sex	-1·684	1·788	272·080	-0·942	0·347	
AIS-C (n = 361)	Intercept	68·324	1·537	373·896	44·448	0·000	0·000
	Age	-4·419	2·404	360·151	-1·838	0·067	
	Time since injury	20·105	0·809	846·053	24·848	0·000	0·000
	Sex	-0·499	1·197	354·491	-0·417	0·677	
AIS-D (n = 499)	Intercept	91·670	0·942	484·828	97·351	0·000	0·000
	Age	-5·653	1·452	467·131	-3·894	0·000	0·001
	Time since injury	8·527	0·508	928·861	16·776	0·000	0·000
	Sex	-0·454	0·694	453·389	-0·654	0·513	
Tetraplegia							
AIS-A (n = 793)	Intercept	19·157	2·068	786·611	9·265	0·000	0·000
	Age	6·649	2·923	779·580	2·275	0·023	
	Time since injury	11·626	0·516	1723·732	22·553	0·000	0·000
	Sex	-0·936	1·658	774·876	-0·565	0·572	
AIS-B (n = 293)	Intercept	25·638	3·210	301·207	7·988	0·000	0·000
	Age	-5·603	4·988	297·539	-1·123	0·262	
	Time since injury	27·892	1·452	707·760	19·213	0·000	0·000
	Sex	-0·321	2·554	291·535	-0·126	0·900	
AIS-C (n = 547)	Intercept	46·661	2·342	584·275	19·922	0·000	0·000
	Age	-14·781	3·230	570·354	-4·576	0·000	0·000
	Time since injury	38·050	1·228	1285·465	30·975	0·000	0·000
	Sex	0·214	1·659	571·121	0·129	0·898	
AIS-D (n = 942)	Intercept	80·154	1·451	964·836	55·251	0·000	0·000
	Age	-8·686	1·969	938·307	-4·412	0·000	0·000
	Time since injury	19·550	0·641	1930·262	30·512	0·000	0·000
	Sex	-0·323	0·954	927·800	-0·339	0·735	
**Functional Independence (SCIM2 and SCIM 3)**							
Paraplegia							

AIS-A (n = 1086)	Intercept	39·967	1·062	1134·421	37·638	0·000	0·000
	Age	-25·712	1·767	1053·448	-14·553	0·000	0·000
	Time since injury	41·850	0·798	2761·804	52·434	0·000	0·000
	Sex	2·221	0·868	1043·709	2·560	0·011	0·085
AIS-B (n = 280)	Intercept	45·602	2·280	279·492	20·002	0·000	0·000
	Age	-25·061	3·846	251·578	-6·516	0·000	0·000
	Time since injury	45·431	1·705	670·751	26·653	0·000	0·000
	Sex	2·139	1·847	246·992	1·158	0·248	
AIS-C (n = 351)	Intercept	55·316	2·244	370·954	24·651	0·000	0·000
	Age	-36·269	3·483	340·547	-10·414	0·000	0·000
	Time since injury	45·278	1·708	841·697	26·510	0·000	0·000
	Sex	2·496	1·705	323·889	1·464	0·144	
AIS-D (n = 482)	Intercept	72·508	2·394	454·322	30·285	0·000	0·000
	Age	-37·528	3·607	415·531	-10·404	0·000	0·000
	Time since injury	43·567	1·707	941·121	25·520	0·000	0·000
	Sex	0·490	1·713	400·882	0·286	0·775	
Tetraplegia							
AIS-A (n = 778)	Intercept	16·239	1·409	790·327	11·524	0·000	0·000
	Age	-11·205	1·998	772·660	-5·608	0·000	0·000
	Time since injury	24·747	0·655	1760·688	37·805	0·000	0·000
	Sex	0·426	1·122	753·323	0·380	0·704	
AIS-B (n = 284)	Intercept	20·327	2·418	304·671	8·407	0·000	0·000
	Age	-17·358	3·713	301·383	-4·675	0·000	0·000
	Time since injury	36·521	1·440	695·945	25·365	0·000	0·000
	Sex	0·660	1·916	290·584	0·345	0·731	
AIS-C (n = 525)	Intercept	37·907	2·268	569·626	16·711	0·000	0·000
	Age	-37·859	3·110	547·327	-12·172	0·000	0·000
	Time since injury	44·444	1·339	1262·868	33·205	0·000	0·000
	Sex	-0·474	1·591	550·400	-0·298	0·766	
AIS-D (n = 913)	Intercept	67·072	2·577	933·335	26·031	0·000	0·000
	Age	-43·266	3·491	892·435	-12·395	0·000	0·000
	Time since injury	54·127	1·448	1924·450	37·375	0·000	0·000
	Sex	-1·583	1·692	884·479	-0·936	0·350	
**Walking function (WISCI)**							
Paraplegia							
AIS-A (n = 1112)	Intercept	0·784	0·281	1133·978	2·795	0·005	0·042
	Age	-0·919	0·473	1100·499	-1·946	0·052	
	Time since injury	2·021	0·132	2547·520	15·334	0·000	0·000
	Sex	0·113	0·233	1101·766	0·484	0·628	
AIS-B (n = 279)	Intercept	2·244	0·764	273·438	2·938	0·004	0·029
	Age	-1·808	1·309	260·553	-1·381	0·168	

	Time since injury	7·697	0·416	619·308	18·523	0·000	0·000
	Sex	-0·101	0·626	255·592	-0·161	0·872	
AIS-C (n = 357)	Intercept	5·413	0·678	385·962	7·983	0·000	0·000
	Age	-7·249	1·045	352·316	-6·934	0·000	0·000
	Time since injury	11·852	0·475	848·450	24·938	0·000	0·000
	Sex	-0·398	0·519	343·661	-0·766	0·444	
AIS-D (n = 477)	Intercept	11·957	0·810	463·369	14·771	0·000	0·000
	Age	-8·983	1·215	434·273	-7·392	0·000	0·000
	Time since injury	11·929	0·519	893·123	22·996	0·000	0·000
	Sex	0·227	0·583	417·855	0·390	0·697	
Tetraplegia							
AIS-A (n = 787)	Intercept	-0·002	0·172	869·784	-0·014	0·989	
	Age	0·170	0·240	834·262	0·710	0·478	
	Time since injury	0·820	0·102	1960·625	8·020	0·000	0·000
	Sex	-0·037	0·135	809·607	-0·275	0·784	
AIS-B (n = 286)	Intercept	0·646	0·501	347·431	1·291	0·198	
	Age	-1·213	0·770	334·636	-1·576	0·116	
	Time since injury	4·231	0·352	769·486	12·032	0·000	0·000
	Sex	-0·326	0·393	324·951	-0·831	0·407	
AIS-C (n = 532)	Intercept	4·269	0·598	593·034	7·140	0·000	0·000
	Age	-6·750	0·818	566·344	-8·252	0·000	0·000
	Time since injury	9·721	0·380	1320·253	25·617	0·000	0·000
	Sex	0·433	0·421	561·326	1·030	0·304	
AIS-D (n = 903)	Intercept	11·638	0·707	897·599	16·468	0·000	0·000
	Age	-8·378	0·956	860·019	-8·766	0·000	0·000
	Time since injury	12·937	0·406	1917·778	31·899	0·000	0·000
	Sex	0·432	0·462	845·977	0·934	0·350	

[#]Time since injury measured in weeks: 2, 4, 8, 16, 26, and 52 weeks. DF: degree of freedom;

Additional File 2: Table S18. Sensitivity Analysis III: Cause of Injury. Model output of total motor score, SCIM2 and 3, and WISCI stratified by plegia, and baseline AIS grades. Patients were enrolled in the EMSCI study. Significant values are highlighted in red.

Model	Variable	Estimate	Standard Error	DF	t-value	p-value	Adjusted p-value
**Total Motor Score**							
Paraplegia							
AIS-A (n = 1132)	Intercept	53·359	1·560	2447·000	34·202	0·000	0·000
	Age	0·302	1·569	1129·000	0·192	0·848	
	Time since injury[#]	2·503	0·190	2447·000	13·171	0·000	0·000
	Cause of injury (Ref: Traumatic)	-0·245	1·320	1129·000	-0·185	0·853	
AIS-B (n = 285)	Intercept	53·102	2·843	614·000	18·676	0·000	0·000
	Age	2·497	3·769	282·000	0·663	0·508	
	Time since injury	13·571	0·774	614·000	17·536	0·000	0·000
	Cause of injury (Ref: Traumatic)	6·364	2·178	282·000	2·922	0·040	
AIS-C (n = 361)	Intercept	63·801	1·945	757·000	32·798	0·000	0·000
	Age	-2·748	2·444	358·000	-1·124	0·262	
	Time since injury	20·115	0·809	757·000	24·878	0·000	0·000
	Cause of injury (Ref: Traumatic)	4·040	1·511	358·000	2·674	0·008	
AIS-D (n = 499)	Intercept	92·037	1·122	753·000	82·045	0·000	0·000
	Age	-5·794	1·470	496·000	-3·942	0·000	0·001
	Time since injury	8·537	0·509	753·000	16·791	0·000	0·000
	Cause of injury (Ref: Traumatic)	-0·757	0·852	496·000	-0·888	0·375	
Tetraplegia							
AIS-A (n = 793)	Intercept	32·339	4·239	1678·000	7·630	0·000	0·000
	Age	5·899	2·869	790·000	2·056	0·040	
	Time since injury	11·631	0·515	1678·000	22·566	0·000	0·000
	Cause of injury (Ref: Traumatic)	-14·006	3·998	790·000	-3·503	0·001	0·004
AIS-B (n = 293)	Intercept	39·688	5·904	656·000	6·723	0·000	0·000
	Age	-8·088	4·934	290·000	-1·639	0·102	
	Time since injury	27·894	1·450	656·000	19·244	0·000	0·000
	Cause of injury (Ref: Traumatic)	-13·928	5·347	290·000	-2·605	0·010	
AIS-C (n = 547)	Intercept	48·440	3·315	1097·000	14·614	0·000	0·000
	Age	-14·773	3·218	544·000	-4·591	0·000	0·000
	Time since injury	38·059	1·228	1097·000	30·983	0·000	0·000
	Cause of injury (Ref: Traumatic)	-1·732	2·957	544·000	-0·586	0·558	
AIS-D (n = 942)	Intercept	73·041	2·248	1665·000	32·498	0·000	0·000
	Age	-8·741	1·940	939·000	-4·505	0·000	0·000

	Time since injury	19·521	0·641	1665·000	30·474	0·000	0·000
	Cause of injury (Ref: Traumatic)	7·186	2·030	939·000	3·539	0·056	
**Functional Independence (SCIM2 and SCIM 3)**							
Paraplegia							
AIS-A (n =1086)	Intercept	44·532	1·872	2226·000	23·793	0·000	0·000
	Age	-26·760	1·853	1083·000	-14·438	0·000	0·000
	Time since injury	41·883	0·798	2226·000	52·459	0·000	0·000
	Cause of injury (Ref: Traumatic)	-2·536	1·583	1083·000	-1·602	0·110	
AIS-B (n = 280)	Intercept	43·977	3·006	570·000	14·631	0·000	0·000
	Age	-23·518	3·963	277·000	-5·935	0·000	0·000
	Time since injury	45·394	1·704	570·000	26·639	0·000	0·000
	Cause of injury (Ref: Traumatic)	3·044	2·293	277·000	1·327	0·186	
AIS-C (n = 351)	Intercept	54·139	2·834	678·000	19·106	0·000	0·000
	Age	-35·552	3·565	348·000	-9·973	0·000	0·000
	Time since injury	45·334	1·708	678·000	26·543	0·000	0·000
	Cause of injury (Ref: Traumatic)	3·121	2·170	348·000	1·439	0·151	
AIS-D (n = 482)	Intercept	79·640	2·726	673·000	29·217	0·000	0·000
	Age	-40·140	3·587	479·000	-11·192	0·000	0·000
	Time since injury	43·689	1·704	673·000	25·646	0·000	0·000
	Cause of injury (Ref: Traumatic)	-6·780	2·038	479·000	-3·327	0·001	0·008
Tetraplegia							
AIS-A (n = 778)	Intercept	20·716	2·991	1583·000	6·925	0·000	0·000
	Age	-11·607	1·973	775·000	-5·884	0·000	0·000
	Time since injury	24·754	0·655	1583·000	37·817	0·000	0·000
	Cause of injury (Ref: Traumatic)	-4·077	2·834	775·000	-1·439	0·151	
AIS-B (n = 284)	Intercept	24·806	4·627	606·000	5·361	0·000	0·000
	Age	-18·308	3·708	281·000	-4·938	0·000	0·000
	Time since injury	36·529	1·440	606·000	25·375	0·000	0·000
	Cause of injury (Ref: Traumatic)	-3·747	4·202	281·000	-0·892	0·373	
AIS-C (n = 525)	Intercept	40·611	3·164	1038·000	12·835	0·000	0·000
	Age	-37·690	3·096	522·000	-12·175	0·000	0·000
	Time since injury	44·470	1·339	1038·000	33·224	0·000	0·000
	Cause of injury (Ref: Traumatic)	-3·371	2·809	522·000	-1·200	0·231	
AIS-D (n = 913)	Intercept	54·850	3·969	1543·000	13·819	0·000	0·000
	Age	-43·128	3·447	910·000	-12·512	0·000	0·000

	Time since injury	54·005	1·448	1543·000	37·303	0·000	0·000
	Cause of injury (Ref: Traumatic)	11·408	3·586	910·000	3·182	0·002	0·012
**Walking function (WISCI)**							
Paraplegia							
AIS-A (n = 1112)	Intercept	0·866	0·495	2317·000	1·749	0·080	
	Age	-0·924	0·496	1109·000	-1·865	0·062	
	Time since injury	2·021	0·132	2317·000	15·333	0·000	0·000
	Cause of injury (Ref: Traumatic)	0·011	0·419	1109·000	0·027	0·979	
AIS-B (n = 279)	Intercept	0·351	1·005	572·000	0·350	0·727	
	Age	-1·102	1·331	276·000	-0·828	0·408	
	Time since injury	7·679	0·416	572·000	18·482	0·000	0·000
	Cause of injury (Ref: Traumatic)	1·804	0·776	276·000	2·324	0·021	
AIS-C (n = 357)	Intercept	2·719	0·841	695·000	3·232	0·001	0·010
	Age	-6·285	1·052	354·000	-5·972	0·000	0·000
	Time since injury	11·859	0·474	695·000	25·002	0·000	0·000
	Cause of injury (Ref: Traumatic)	2·334	0·649	354·000	3·599	0·000	0·003
AIS-D (n = 477)	Intercept	12·868	0·939	654·000	13·702	0·000	0·000
	Age	-9·314	1·220	474·000	-7·634	0·000	0·000
	Time since injury	11·939	0·519	654·000	23·019	0·000	0·000
	Cause of injury (Ref: Traumatic)	-0·714	0·712	474·000	-1·003	0·316	
Tetraplegia							
AIS-A (n = 787)	Intercept	0·361	0·358	1633·000	1·007	0·314	
	Age	0·157	0·236	784·000	0·663	0·508	
	Time since injury	0·821	0·102	1633·000	8·028	0·000	0·000
	Cause of injury (Ref: Traumatic)	-0·397	0·339	784·000	-1·173	0·241	
AIS-B (n = 286)	Intercept	0·174	0·988	628·000	0·176	0·860	
	Age	-1·049	0·765	283·000	-1·373	0·171	
	Time since injury	4·231	0·352	628·000	12·025	0·000	0·000
	Cause of injury (Ref: Traumatic)	0·162	0·905	283·000	0·179	0·858	
AIS-C (n = 532)	Intercept	4·462	0·857	1057·000	5·204	0·000	0·000
	Age	-6·824	0·816	529·000	-8·364	0·000	0·000
	Time since injury	9·721	0·380	1057·000	25·615	0·000	0·000
	Cause of injury (Ref: Traumatic)	0·185	0·762	529·000	0·242	0·809	
AIS-D (n = 903)	Intercept	8·917	1·128	1534·000	7·908	0·000	0·000
	Age	-8·574	0·944	900·000	-9·080	0·000	0·000

Time since injury	12·932	0·405	1534·000	31·916	0·000	0·000
Cause of injury (Ref: Traumatic)	3·281	1·028	900·000	3·191	0·012	

#Time since injury measured in weeks: 2, 4, 8, 16, 26, and 52 weeks. DF: degree of freedom.
