# Supplementary material for: International surveillance study in acute spinal cord injury confirms viability of multinational clinical trials
Source: BMC Med. 2022 Jun 14;20:225. doi: 10.1186/s12916-022-02395-0 (PMC9202190; doi:10.1186/s12916-022-02395-0)
Supplement: Supplementary file 3 — Additional file 3: Figure S1. Flow chart of included and excluded subjects. Figure S2. Proportion of missing data in the Sygen trial stratified by the exam stage. Figure S3. Proportion of missing data in the Sygen trial stratified by the injury severity (i.e., AIS grade). Figure S4. Sensorimotor recovery after spinal cord injury in patients enrolled in the Sygen clinical trial. Recovery of (A) lower and (B) upper extremity motor score as well as (C) total light touch and (D) total pin prick are all dependent on the injury level (tetra- vs. paraplegia) and the injury severity (i.e., AIS grades). Table S1. Details on excluded Sygen cohort. Table S2. Outcome variables and number of observations with missing data (Sygen). Table S3. Model output of longitudinal analysis of demographics (i.e., sex and age) and baseline injury characteristics (i.e., injury severity and level, plegia). Table S4. Model output of lower extremity motor score (LEMS) stratified by sex, plegia, and AIS grades. Patients were enrolled in the Sygen Trial. Note: In case the number of patients per group were too low (n < 3), the model did not converge and thus, the results are not represented in the table. Table S5. Model output of upper extremity motor score (UEMS) stratified by sex and AIS grades. Patients were enrolled in the Sygen Trial. Note: This analysis was only conducted for the tetraplegic patients. Table S7. Model output of total light touch (TLT) stratified by sex, plegia, and AIS grades. Patients were enrolled in the Sygen Trial. Note: In case the number of patients per group were too low (n < 3), the model did not converge and thus, the results are not represented in the table. Table S8. Model output of total pinprick (TPP) stratified by sex, plegia, and AIS grades. Patients were enrolled in the Sygen Trial. Note: In case the number of patients per group were too low (n < 3), the model did not converge and thus, the results are not represented in the table. Table S9. Sensitivity analysis [file 12916_2022_2395_MOESM3_ESM.pdf]

Additional File 3

1. Data source: Sygen® Trial

Sponsored by the United States Food and Drug Administration (FDA)-sponsored, the Sygen trial is a completed randomized, prospective, phase III, placebo controlled, multi-center study testing the efficacy of GM-1 ganglioside therapy in acute, traumatic spinal cord injury. Full design, recruitment, and enrollment details have been published previously.[28] Briefly, patients were required to have at least one lower extremity with a substantial motor deficit to be included in the Sygen trial. Patients with spinal cord transection or penetration were excluded, as were patients with a cauda equina, brachial or lumbosacral plexus, or peripheral nerve injury. Multiple trauma cases were included as long as they were not so severe as to preclude neurologic evaluation. Major head trauma cases and also intubated chest trauma cases were also excluded. With 797 enrolled patients, the Sygen trial was the largest clinical trial ever conducted in the field of spinal cord injury.

Out of 549 patients who were operated on, 515 received surgical decompression, 107 of which within the first 24 hours post injury. Surgical decompression was at the discretion of the treating physician.

The negative finding of the Sygen study is considered Class I Medical Evidence by the spinal cord injury Committee of the American Association of Neurological Surgeons (AANS) and the Congress of Neurological Surgeons (CNS).

Data availability

Full anonymized data will be shared at the request from any qualified investigator (please contact the Corresponding Author).

Standard Protocol Approvals, Registrations, and Patient Consents

Approval for this study (secondary analysis) was received by an institutional ethical standards committee on human experimentation at the University of British Columbia. The original Sygen clinical trial (results published elsewhere)[2,3] also received ethical approval, but was conducted before clinical trials were required to be registered (i.e., no clinicaltrial.gov identifier available). The data received from the original clinical trial were de-identified in compliance with Health Insurance Portability and Accountability Act (HIPPA).

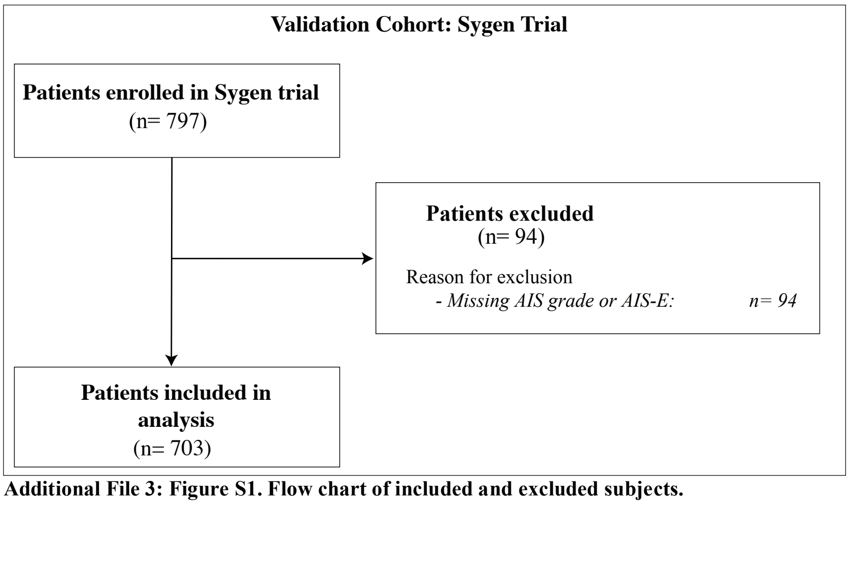

Additional File 3: Figure S1. Flow chart of included and excluded subjects.

2. Excluded cohort

A total of 94 patients (mean age at injury, 31·1 ± 12·4 years; 88·3% male) were excluded from our baseline summary analysis (**Table A2.1.**). The ratio of male and female patients was comparable between included and excluded cohorts (X-squared = 4·402, df = 1, p-value = 0·036). However, in terms of age (t = -2·033, df = 124·56, p-value = 0·044) the cohorts were different. Specifically, the excluded cohort was slightly younger compared to the included cohort.

Additional File 3: Table S1. Details on excluded Sygen cohort

	Overall (N=94)
**Sex**	
Female	11 (11·7%)
Male	83 (88·3%)
**Age (years)**	
Mean (SD)	30·1 (12·4)
Median [Min, Max]	28·0 [14·0, 65·0]
**AIS grade**	
A (complete)	0 (0%)
B (sensory incomplete)	0 (0%)
C (motor incomplete)	0 (0%)
D (motor incomplete)	0 (0%)
Missing	94 (100%)
**Neurological level of injury**	
Cervical	60 (63·8%)
Thoracic	34 (36·2%)

3. Missing data

Approximately 7·2% of the data was missing. Variables and number of patients with missing are listed in Table A2.2.

Additional File 3: Table S2. Outcome variables and number of observations with missing data (Sygen)

Variable	Count (n)	Proportion [%]
Lower extremity motor score	253	5·6
Upper extremity motor score	455	9·9
Total motor score	532	11·8
Total light touch	813	18·1
Total pin prick	813	18·1
Benzel Score	1381	30·7

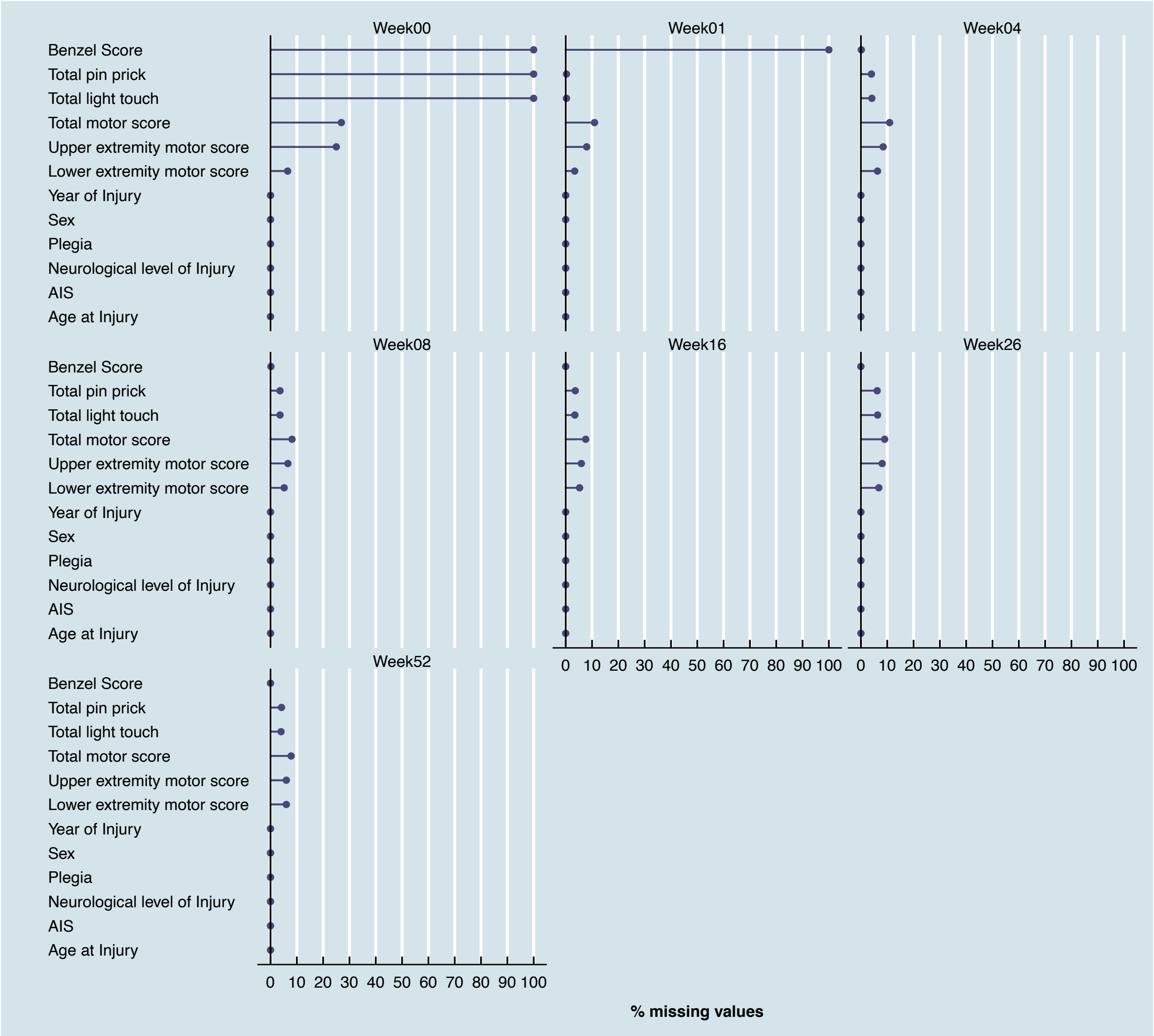

Additional File 3: Figure S2. Proportion of missing data in the Sygen trial stratified by the exam stage.

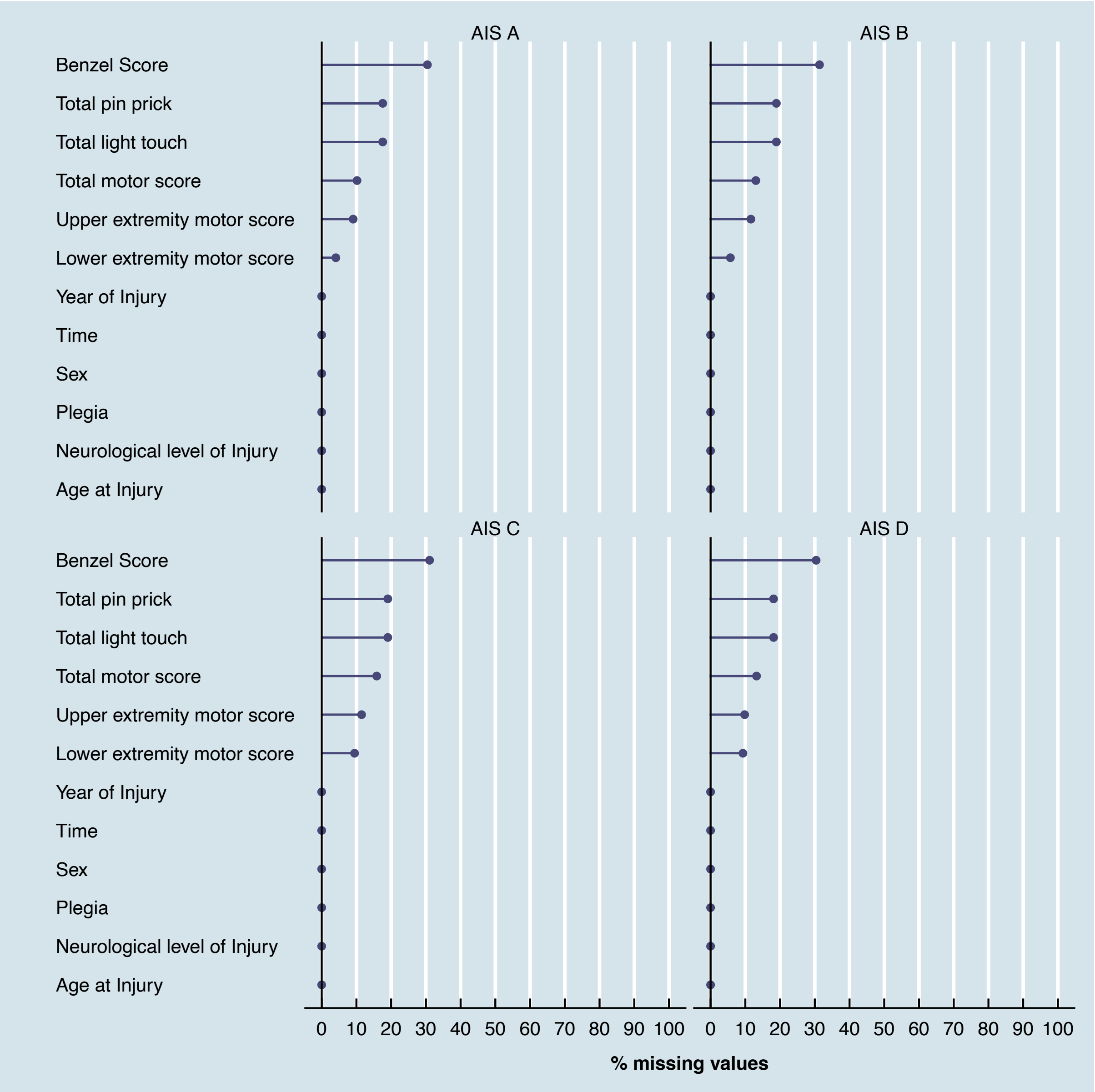

Additional File 3: Figure S3. Proportion of missing data in the Sygen trial stratified by the injury severity (i.e., AIS grade).

4. Recovery after Spinal Cord Injury

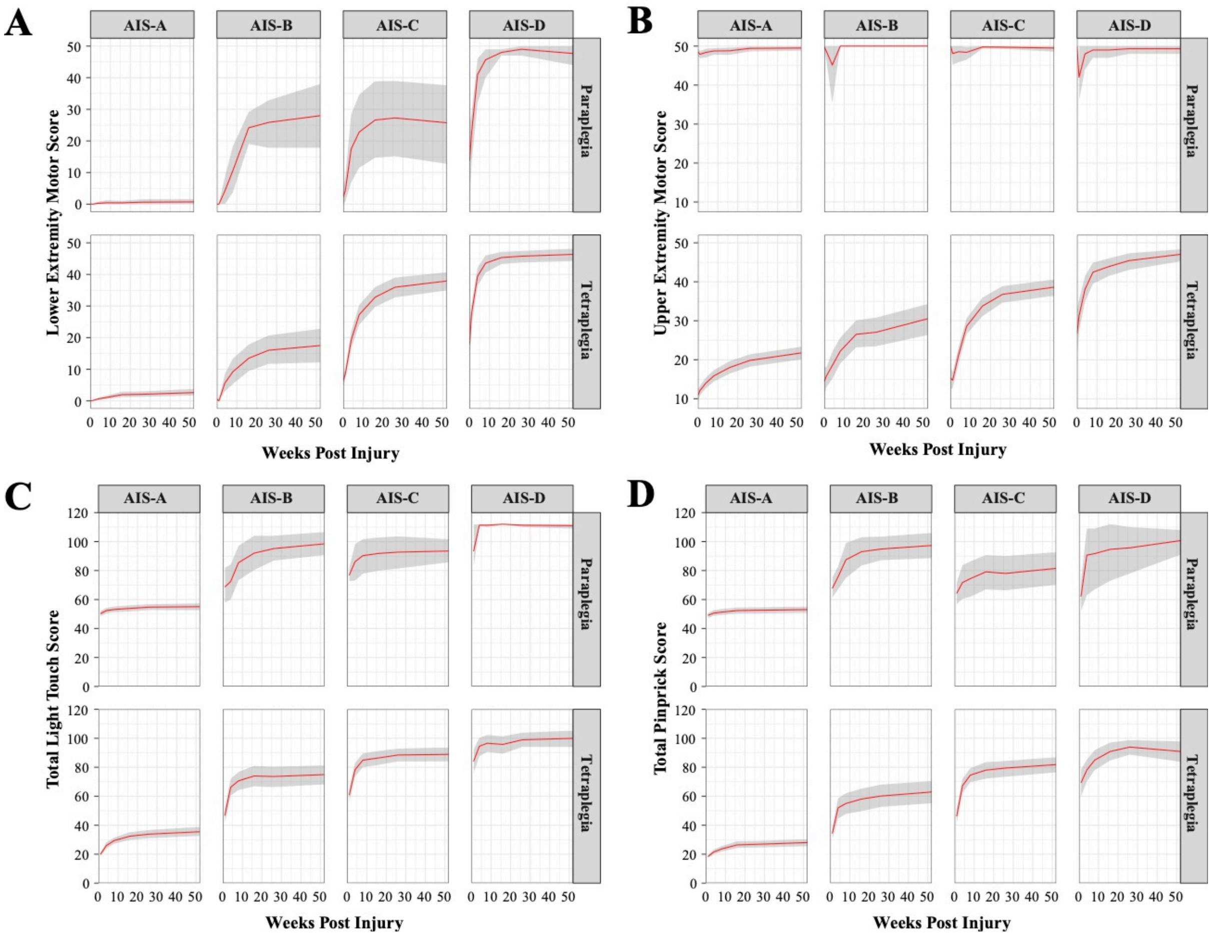

Additional File 3: Figure S4. **Sensorimotor recovery after spinal cord injury in patients enrolled in the Sygen clinical trial.** Recovery of **(A)** lower and **(B)** upper extremity motor score as well as **(C)** total light touch and **(D)** total pin prick are all dependent on the injury level (tetra- vs. paraplegia) and the injury severity (i.e., AIS grades).

Additional File 3: Table S3. Model output of longitudinal analysis of demographics (i.e., sex and age) and baseline injury characteristics (i.e., injury severity and level, plegia).

Model		Coefficients	Estimate	Std· Error	t-value	p-value	Adjusted p-value
Age distribution							
Overall		Intercept	32·687	0·928	35·210	<0·001	Not applicable
		YEARDOI	0·391	1·782	0·220	0·826	Not applicable
Overall: Effect of sex		Intercept	34·643	2·017	17·175	<0·001	Not applicable
		YEARDOI	-1·523	3·955	-0·385	0·700	Not applicable
		Sex	-2·489	2·272	-1·095	0·274	Not applicable
		YEARDOI*Sex	2·461	4·431	0·555	0·579	Not applicable
Paraplegia							
	AIS-A	Intercept	27·986	1·686	16·602	<0·001	<0·001
		YEARDOI	1·336	3·294	0·405	0·686	
	AIS-B	Intercept	34·927	7·774	4·493	0·004	0·033
		YEARDOI	-10·104	12·781	-0·791	0·459	
	AIS-C	Intercept	33·167	7·481	4·434	<0·001	0·008
		YEARDOI	-2·083	12·743	-0·163	0·873	
	AIS-D	Intercept	31·333	2·494	12·561	0·051	
		YEARDOI	<0·001	5·774	<0·001	1·000	
Tetraplegia							
	AIS-A	Intercept	32·278	1·368	23·594	<0·001	<0·001
		YEARDOI	0·812	2·552	0·318	0·751	
	AIS-B	Intercept	32·630	3·440	9·486	<0·001	<0·001
		YEARDOI	3·068	6·527	0·470	0·640	
	AIS-C	Intercept	36·843	2·350	15·681	<0·001	<0·001
		YEARDOI	-0·801	4·680	-0·171	0·864	
	AIS-D	Intercept	35·275	4·765	7·403	<0·001	<0·001
		YEARDOI	3·186	11·911	0·267	0·791	
Sex ratio							
Overall		Intercept	3·473	0·405	8·582	0·001	
		YEARDOI	1·247	0·668	1·866	0·135	
Injury severity							
	AIS-A	Intercept	4·243	0·447	9·501	0·001	0·004
		YEARDOI	-0·067	0·738	-0·091	0·932	
	AIS-B	Intercept	5·120	2·632	1·946	0·124	
		YEARDOI	-2·212	4·346	-0·509	0·638	
	AIS-C	Intercept	4·101	2·793	1·468	0·216	
		YEARDOI	-0·444	4·612	-0·096	0·928	
	AIS-D	Intercept	1·444	-0·776	1·863	0·136	
		YEARDOI	-7·962e-16	1·281	<0·001	1·000	
Plegia							
	Paraplegia	Intercept	5·823	0·611	9·534	0·001	0·002
		YEARDOI	-3·951	1·009	-3·917	0·017	
	Tetraplegia	Intercept	2·430	1·170	2·078	0·106	
		YEARDOI	4·565	1·932	2·363	0·077	
AIS grade							
Overall							
	AIS-A	Intercept	28·945	12·121	2·388	0·038	0·152
		YEARDOI	9·833	20·017	0·491	0·634	
	AIS-B	Intercept	4·390	1·938	2·266	0·053	
		YEARDOI	2·891	3·955	0·731	0·486	
	AIS-C	Intercept	11·040	3·682	2·999	0·015	0·060
		YEARDOI	-1·156	6·622	-0·175	0·865	
	AIS-D	Intercept	3·363	1·351	2·490	0·042	0·168
		YEARDOI	2·148	2·707	-0·794	0·454	
Paraplegia							
	AIS-A	Intercept	19·152	2·825	6·778	0·002	0·020
		YEARDOI	-2·034	4·666	-0·436	0·685	
	AIS-B	Intercept	1·188	0·886	1·341	0·312	
		YEARDOI	0·642	1·738	0·369	0·747	
	AIS-C	Intercept	0·914	0·482	1·894	0·154	
		YEARDOI	2·281	0·984	2·318	0·103	
	AIS-D	Intercept	0·609	0·138	4·400	0·142	
		YEARDOI	0·246	0·320	0·767	0·583	
Tetraplegia							
	AIS-A	Intercept	38·739	8·262	4·689	0·009	0·075
		YEARDOI	21·700	13·644	1·590	0·187	
	AIS-B	Intercept	7·881	1·953	4·035	0·027	0·219
		YEARDOI	5·141	3·987	1·289	0·288	
	AIS-C	Intercept	22·439	2·994	7·496	0·002	0·014
		YEARDOI	-9·371	4·944	-1·895	0·131	
	AIS-D	Intercept	5·821	1·873	3·108	0·053	
		YEARDOI	-4·346	3·823	-1·137	0·338	

Additional File 3: Table S4. Model output of lower extremity motor score (LEMS) stratified by sex, plegia, and AIS grades. Patients were enrolled in the Sygen Trial. Note: In case the number of patients per group were too low (n<3), the model did not converge and thus, the results are not represented in the table.

Model	Variable	Estimate	Standard Error	DF	t-val
Female *paraplegic* patients					
AIS-A (n = 27)	Intercept	0·901	2·907	29·275	0·3
	Age	-2·199	5·466	24·316	-0·4
	Time since Injury	-2·254	1·810	148·156	-1·2
	YEARDOI	-0·714	3·909	34·504	-0·1
	Time since Injury *YEARDOI	10·216	3·207	146·613	3·1
Female *tetraplegic* patients					
AIS-A (n = 60)	Intercept	0·238	1·433	69·984	0·1
	Age	-0·205	2·269	59·472	-0·0
	Time since Injury	0·131	0·907	311·296	0·1
	YEARDOI	-0·416	1·985	82·687	-0·2
	Time since Injury *YEARDOI	5·438	1·726	311·802	3·1
AIS-B (n = 19)	Intercept	0·965	7·580	21·387	0·1
	Age	-8·940	12·860	15·825	-0·6
	Time since Injury	29·204	6·122	90·729	4·7
	YEARDOI	4·047	13·731	24·221	0·2
	Time since Injury *YEARDOI	-19·115	13·246	92·121	-1·4
AIS-C (n = 29)	Intercept	14·007	4·523	34·612	3·0
	Age	-15·064	7·270	25·632	-2·0
	Time since Injury	39·202	3·275	152·639	11·9
	YEARDOI	1·994	7·750	43·472	0·2
	Time since Injury *YEARDOI	-1·856	7·396	152·002	-0·2
AIS-D (n = 5)	Intercept	36·167	6·151	2·393	5·8
	Age	-6·810	9·589	1·928	-0·7
	Time since Injury	15·259	4·126	23·156	3·6
	YEARDOI	-21·498	10·225	3·349	-2·1
	Time since Injury *YEARDOI	16·120	10·307	22·444	1·5
Male *paraplegic* patients					
AIS-A (n = 105)	Intercept	0·072	0·184	228·119	0·3
	Age	-0·440	0·348	104·760	-1·2
	Time since Injury	0·589	0·216	588·869	2·7
	YEARDOI	0·180	0·313	329·759	0·5
	Time since Injury *YEARDOI	-0·935	0·437	591·001	-2·1
AIS-B (n = 8)	Intercept	0·484	6·698	7·056	0·0
	Age	-6·352	11·822	5·263	-0·5
	Time since Injury	36·242	5·304	39·484	6·8
	YEARDOI	-3·521	7·968	10·425	-0·4
	Time since Injury *YEARDOI	-7·957	9·354	40·897	-0·8
AIS-C (n = 12)	Intercept	13·577	14·071	10·411	0·9
	Age	-19·713	23·657	8·896	-0·8
	Time since Injury	20·800	7·468	56·846	2·7
	YEARDOI	-2·848	19·099	11·259	-0·1
	Time since Injury *YEARDOI	1·054	12·635	56·467	0·0
Male *tetraplegic* patients					
AIS-A (n = 252)	Intercept	-0·265	0·668	327·091	-0·3
	Age	0·795	1·175	255·894	0·6
	Time since Injury	3·291	0·496	1319·769	6·6
	YEARDOI	-0·495	0·986	376·979	-0·5
	Time since Injury *YEARDOI	-0·939	0·909	1313·800	-1·0
AIS-B (n = 50)	Intercept	1·967	3·892	64·663	0·5
	Age	-9·116	5·676	47·961	-1·6
	Time since Injury	14·025	3·178	262·989	4·4
	YEARDOI	1·606	5·830	75·189	0·2
	Time since Injury *YEARDOI	10·241	5·839	262·320	1·7
AIS-C (n = 105)	Intercept	5·260	3·242	123·603	1·6
	Age	3·342	4·670	102·870	0·7
	Time since Injury	34·210	2·142	496·111	15·9
	YEARDOI	-0·870	4·773	140·481	-0·1
	Time since Injury *YEARDOI	-1·899	4·140	497·445	-0·4
AIS-D (n = 23)	Intercept	24·613	4·266	29·369	5·7
	Age	0·677	6·459	18·880	0·1
	Time since Injury	28·263	3·845	113·833	7·3
	YEARDOI	-3·825	8·499	37·084	-0·4
	Time since Injury *YEARDOI	1·157	10·291	118·595	0·1

Additional File 3: Table S5. Model output of upper extremity motor score (UEMS) stratified by sex and AIS grades. Patients were enrolled in the Sygen Trial. Not
conducted for the tetraplegic patients.

Model	Variable	Estimate	Standard Error	DF	t-value	p-value
Female *tetraplegic* patients						
AIS-A (n = 59)	Intercept	16·815	4·142	57·624	4·059	<0·001
	Age	-5·101	6·833	56·324	-0·747	0·459
	Time since Injury	9·156	0·945	288·125	9·689	<0·001
	YEARDOI	-3·363	5·610	58·761	-0·599	0·551
	Time since Injury *YEARDOI	-1·438	1·769	288·106	-0·813	0·417
AIS-B (n = 19)	Intercept	21·414	6·684	18·457	3·204	0·005
	Age	-11·019	11·705	15·442	-0·941	0·361
	Time	5·825	3·927	81·377	1·484	0·142
	YEARDOI	-10·163	11·943	19·865	-0·851	0·405
	Time since Injury *YEARDOI	27·328	8·551	82·510	3·196	0·002
AIS-C (n = 29)	Intercept	17·130	3·698	33·835	4·632	<0·001
	Age	-11·935	6·010	26·211	-1·986	0·058
	Time since Injury	29·186	2·549	141·350	11·451	<0·001
	YEARDOI	3·083	6·317	41·871	0·488	0·628
	Time since Injury *YEARDOI	-1·297	5·788	140·900	-0·224	0·823
AIS-D (n = 5)	Intercept	29·421	8·268	1·888	3·558	0·077
	Age	-13·460	13·459	1·802	-1·000	0·432
	Time since Injury	28·951	2·605	22·194	11·113	<0·001
	YEARDOI	2·094	13·005	2·142	0·161	0·886
	Time since Injury *YEARDOI	-25·083	6·769	21·921	-3·706	0·001
Male *tetraplegic* patients						
AIS-A (n = 253)	Intercept	15·919	1·707	266·949	9·327	<0·001
	Age	-9·102	3·108	251·783	-2·928	0·004
	Time since Injury	9·069	0·636	1219·227	14·254	<0·001
	YEARDOI	-4·248	2·445	277·576	-1·737	0·084
	Time since Injury *YEARDOI	2·141	1·164	1217·206	1·840	0·066
AIS-B (n = 49)	Intercept	26·170	4·605	51·885	5·684	<0·001
	Age	-11·705	6·989	45·445	-1·675	0·101
	Time since Injury	12·430	2·338	237·283	5·316	<0·001
	YEARDOI	-13·324	6·786	54·239	-1·963	0·055
	Time since Injury *YEARDOI	6·276	4·242	236·605	1·479	0·140
AIS-C (n = 107)	Intercept	17·164	2·899	122·063	5·921	<0·001
	Age	-8·161	4·126	101·429	-1·978	0·051
	Time since Injury	26·474	1·761	489·094	15·032	<0·001
	YEARDOI	0·104	4·169	133·218	0·025	0·980
	Time since Injury *YEARDOI	-0·018	3·313	488·768	-0·005	0·996
AIS-D (n = 23)	Intercept	32·743	3·568	31·816	9·177	<0·001
	Age	-5·715	5·412	20·562	-1·056	0·303
	Time since Injury	23·041	3·174	114·173	7·259	<0·001
	YEARDOI	3·351	7·353	43·452	0·456	0·651
	Time since Injury *YEARDOI	-12·778	8·581	116·395	-1·489	0·139

Additional File 3: Table S6. Model output of total motor score (TMS) stratified by sex, plegia, and AIS grades. Patients were enrolled in the Sygen Trial. Note: In c
were too low (n<3), the model did not converge and thus, the results are not represented in the table.

Model	Variable	Estimate	Standard Error	DF	t-value
Female *paraplegic* patients					
AIS-A (n = 27)	Intercept	50·522	3·903	32·757	12·946
	Age	-12·735	7·091	23·762	-1·796
	Time since Injury	-0·324	3·179	152·560	-0·102
	YEARDOI	-4·456	5·392	42·639	-0·827
	Time since Injury *YEARDOI	17·799	5·614	150·401	3·171
Female *tetraplegic* patients					
AIS-A (n = 60)	Intercept	15·816	4·624	59·999	3·420
	Age	-5·433	7·551	57·769	-0·719
	Time since Injury	10·400	1·483	309·018	7·015
	YEARDOI	-3·177	6·203	62·590	-0·512
	Time since Injury *YEARDOI	4·323	2·803	309·054	1·542
AIS-B (n = 19)	Intercept	20·383	12·184	20·289	1·673
	Age	-20·149	20·923	15·722	-0·963
	Time since Injury	37·775	9·090	92·224	4·156

	YEARDOI	-3·694	21·948	22·517	-0·168
	Time since Injury *YEARDOI	5·402	19·651	93·493	0·275
AIS-C (n = 29)	Intercept	30·834	7·746	32·130	3·981
	Age	-27·160	12·723	25·909	-2·135
	Time since Injury	68·551	4·938	157·108	13·881
	YEARDOI	5·959	13·013	37·542	0·458
	Time since Injury *YEARDOI	-3·931	10·967	156·459	-0·358
AIS-D (n = 5)	Intercept	65·348	12·034	1·990	5·431
	Age	-20·168	19·398	1·827	-1·040
	Time since Injury	44·142	5·164	23·533	8·548
	YEARDOI	-17·381	19·026	2·304	-0·914
	Time since Injury *YEARDOI	-10·714	12·619	23·065	-0·849
Male *paraplegic* patients					
AIS-A (n = 105)	Intercept	42·123	1·546	168·440	27·240
	Age	-7·039	3·151	97·833	-2·234
	Time since Injury	10·549	1·557	585·166	6·776
	YEARDOI	8·230	2·548	227·901	3·230
	Time since Injury *YEARDOI	-8·344	3·135	587·670	-2·662
AIS-B (n = 8)	Intercept	48·248	8·454	6·770	5·707
	Age	-3·381	15·043	5·226	-0·225
	Time since Injury	37·189	6·274	40·368	5·928
	YEARDOI	-4·749	9·855	9·275	-0·482
	Time since Injury *YEARDOI	-5·640	10·817	41·381	-0·521
AIS-C (n = 12)	Intercept	40·851	16·389	9·994	2·493
	Age	-13·856	27·816	8·863	-0·498
	Time since Injury	41·215	7·812	58·487	5·276
	YEARDOI	26·051	22·194	10·714	1·174
	Time since Injury *YEARDOI	-28·946	13·412	58·235	-2·158
Male *tetraplegic* patients					
AIS-A (n = 254)	Intercept	14·559	1·858	287·003	7·835
	Age	-8·206	3·333	254·628	-2·462
	Time since Injury	14·028	1·004	1332·975	13·971
	YEARDOI	-4·082	2·685	310·454	-1·520
	Time since Injury *YEARDOI	0·112	1·846	1328·793	0·060
AIS-B (n = 50)	Intercept	26·600	6·732	57·284	3·952
	Age	-20·177	10·087	47·157	-2·000
	Time since Injury	27·541	4·478	267·594	6·150
	YEARDOI	-8·994	9·972	64·025	-0·902
	Time since Injury *YEARDOI	15·552	8·250	267·199	1·885
AIS-C (n = 107)	Intercept	21·567	4·852	123·642	4·445
	Age	-4·350	6·954	105·218	-0·626
	Time since Injury	61·987	3·194	547·798	19·411
	YEARDOI	0·798	7·084	142·720	0·113
	Time since Injury *YEARDOI	-6·174	6·210	549·234	-0·994
AIS-D (n = 23)	Intercept	51·807	5·046	39·359	10·268
	Age	-1·874	7·137	19·462	-0·263
	Time since Injury	56·922	5·992	127·212	9·499
	YEARDOI	0·829	10·616	58·551	0·078
	Time since Injury *YEARDOI	-13·880	15·998	132·336	-0·868

Additional File 3: Table S7. Model output of total light touch (TLT) stratified by sex, plegia, and AIS grades. Patients were enrolled in the Sygen Trial. Note: In ca[illegible] patients per group were too low (n<3), the model did not converge and thus, the results are not represented in the table.

Model	Variable	Estimate	Standard Error	DF	t-value	p-value
Female *paraplegic* patients						
AIS-A (n = 27)	Intercept	54·821	7·379	32·854	7·430	<0·001
	Age	-9·757	13·432	24·046	-0·726	0·475
	Time since Injury	-3·477	5·047	124·711	-0·689	0·492
	YEARDOI	-4·980	10·131	41·627	-0·492	0·626
	Time since Injury*YEARDOI	20·663	8·829	123·405	2·340	0·021
Female *tetraplegic* patients						
AIS-A (n = 60)	Intercept	27·500	6·529	76·261	4·212	<0·001
	Age	-9·754	10·176	60·814	-0·959	0·342
	Time since Injury	14·408	4·136	257·206	3·483	0·001
	YEARDOI	-4·738	9·142	93·689	-0·518	0·606
	Time since Injury*YEARDOI	10·552	7·763	257·129	1·359	0·175
AIS-B (n = 19)	Intercept	52·190	18·949	23·152	2·754	0·011
	Age	3·962	31·254	15·372	0·127	0·901
	Time since Injury	37·130	14·728	72·855	2·521	0·014
	YEARDOI	-9·167	34·702	27·185	-0·264	0·794
	Time since Injury*YEARDOI	-1·311	31·806	73·866	-0·041	0·967
AIS-C (n = 29)	Intercept	72·609	9·978	37·281	7·277	<0·001
	Age	-24·226	15·856	26·564	-1·528	0·138
	Time since Injury	37·819	6·824	131·733	5·542	<0·001
	YEARDOI	4·531	17·066	46·458	0·266	0·792
	Time since Injury*YEARDOI	-12·737	15·197	131·451	-0·838	0·404
AIS-D (n = 5)	Intercept	90·760	19·897	1·587	4·561	0·068
	Age	-25·550	31·244	1·306	-0·818	0·536
	Time since Injury	27·839	10·545	18·209	2·640	0·017
	YEARDOI	11·536	32·590	2·126	0·354	0·755
	Time since Injury*YEARDOI	-5·600	25·787	17·620	-0·217	0·831
Male *paraplegic* patients						
AIS-A (n = 105)	Intercept	54·562	2·241	126·802	24·346	<0·001
	Age	-13·804	4·960	101·649	-2·783	0·006
	Time since Injury	6·389	1·281	477·683	4·987	<0·001
	YEARDOI	-0·416	3·532	147·043	-0·118	0·906
	Time since Injury*YEARDOI	-3·996	2·596	478·723	-1·540	0·124
AIS-B (n = 8)	Intercept	66·384	14·911	7·413	4·452	0·003
	Age	14·177	25·991	5·283	0·546	0·608
	Time since Injury	19·674	10·651	32·516	1·847	0·074
	YEARDOI	-16·635	17·849	11·058	-0·932	0·371
	Time since Injury*YEARDOI	41·548	18·370	33·192	2·262	0·030
AIS-C (n = 12)	Intercept	83·657	14·242	11·145	5·874	<0·001
	Age	-17·442	23·576	8·978	-0·740	0·478
	Time since Injury	18·302	7·277	45·598	2·515	0·016
	YEARDOI	-3·194	19·514	12·476	-0·164	0·873
	Time since Injury*YEARDOI	-2·663	12·621	45·405	-0·211	0·834
Male *tetraplegic* patients						
AIS-A (n = 254)	Intercept	21·099	2·636	355·562	8·005	<0·001
	Age	-9·759	4·503	260·122	-2·167	0·031
	Time since Injury	19·352	1·904	1092·265	10·165	<0·001
	YEARDOI	1·701	3·925	428·791	0·434	0·665
	Time since Injury*YEARDOI	-4·173	3·519	1087·972	-1·186	0·236
AIS-B (n = 50)	Intercept	71·180	8·134	91·543	8·751	<0·001
	Age	-16·140	10·652	45·583	-1·515	0·137
	Time since Injury	15·072	8·080	219·397	1·865	0·064
	YEARDOI	-33·202	12·738	121·081	-2·607	0·010
	Time since Injury*YEARDOI	30·180	14·737	218·895	2·048	0·042
AIS-C (n = 106)	Intercept	61·737	6·354	149·939	9·716	<0·001
	Age	-1·373	8·696	105·611	-0·158	0·875
	Time since Injury	34·289	4·986	442·729	6·878	<0·001
	YEARDOI	5·309	9·611	195·733	0·552	0·581
	Time since Injury*YEARDOI	-7·830	9·708	443·544	-0·807	0·420
AIS-D (n = 23)	Intercept	91·861	8·288	36·208	11·084	<0·001
	Age	2·663	12·123	20·559	0·220	0·828
	Time since Injury	12·842	7·664	102·672	1·676	0·097
	YEARDOI	-18·959	17·315	52·144	-1·095	0·279
	Time since Injury*YEARDOI	6·487	20·702	105·428	0·313	0·755

Additional File 3: Table S8. Model output of total pinprick (TPP) stratified by sex, plegia, and AIS grades. Patients were enrolled in the Sygen Trial. Note: In case per group were too low (n<3), the model did not converge and thus, the results are not represented in the table.

Model	Variable	Estimate	Standard Error	DF	t-value	p-value
Female *paraplegic* patients						
AIS-A (n = 27)	Intercept	51·630	7·268	31·242	7·103	<0·001
	Age	-7·966	13·398	24·008	-0·595	0·558
	Time since Injury	1·130	4·596	124·303	0·246	0·806
	YEARDOI	-2·103	9·892	38·410	-0·213	0·833
	Time since Injury*YEARDOI	10·567	8·036	123·114	1·315	0·191
Female *tetraplegic* patients						
AIS-A (n = 60)	Intercept	22·429	4·627	79·558	4·848	<0·001
	Age	-3·808	7·141	61·005	-0·533	0·596
	Time since Injury	4·648	3·148	257·763	1·476	0·141
	YEARDOI	-3·581	6·531	100·606	-0·548	0·585
	Time since Injury*YEARDOI	12·692	5·909	257·690	2·148	0·033
AIS-B (n = 19)	Intercept	42·944	18·280	23·895	2·349	0·027
	Age	-20·461	30·066	15·745	-0·681	0·506
	Time since Injury	35·974	14·371	73·246	2·503	0·015
	YEARDOI	-5·202	33·508	28·106	-0·155	0·878
	Time since Injury*YEARDOI	-2·584	31·033	74·250	-0·083	0·934
AIS-C (n = 29)	Intercept	68·062	11·521	38·208	5·908	<0·001
	Age	-31·466	18·183	26·541	-1·731	0·095
	Time since Injury	36·052	8·150	131·775	4·423	<0·001
	YEARDOI	-11·447	19·781	48·273	-0·579	0·566
	Time since Injury*YEARDOI	18·560	18·152	131·494	1·023	0·308
AIS-D (n = 5)	Intercept	57·985	27·996	1·696	2·071	0·197
	Age	-10·554	45·048	1·544	-0·234	0·842
	Time since Injury	54·457	10·507	18·055	5·183	<0·001
	YEARDOI	45·324	44·281	1·971	1·024	0·415
	Time since Injury*YEARDOI	-50·905	25·596	17·727	-1·989	0·062
Male *paraplegic* patients						
AIS-A (n = 105)	Intercept	52·247	2·249	131·900	23·229	<0·001
	Age	-11·206	4·926	101·506	-2·275	0·025
	Time since Injury	4·730	1·394	478·024	3·394	0·001
	YEARDOI	0·376	3·568	156·607	0·105	0·916
	Time since Injury*YEARDOI	-2·245	2·823	479·179	-0·795	0·427
AIS-B (n = 8)	Intercept	69·410	10·002	9·152	6·939	<0·001
	Age	6·342	16·457	5·204	0·385	0·715
	Time since Injury	15·241	8·931	32·615	1·707	0·097
	YEARDOI	-14·440	12·625	15·931	-1·144	0·270
	Time since Injury*YEARDOI	46·875	15·350	33·383	3·054	0·004
AIS-C (n = 12)	Intercept	80·461	12·674	11·452	6·348	<0·001
	Age	-39·956	20·886	9·073	-1·913	0·088
	Time since Injury	12·631	6·720	45·730	1·880	0·067
	YEARDOI	-5·761	17·404	12·919	-0·331	0·746
	Time since Injury*YEARDOI	8·226	11·656	45·527	0·706	0·484
Male *tetraplegic* patients						
AIS-A (n = 254)	Intercept	19·923	2·212	370·434	9·008	<0·001
	Age	-9·389	3·739	260·094	-2·511	0·013
	Time since Injury	13·296	1·692	1094·515	7·860	<0·001
	YEARDOI	0·980	3·312	455·177	0·296	0·768
	Time since Injury*YEARDOI	-3·899	3·124	1090·001	-1·248	0·212
AIS-B (n = 50)	Intercept	55·912	8·772	78·899	6·374	<0·001
	Age	-30·882	12·107	47·770	-2·551	0·014
	Time since Injury	23·099	7·574	220·015	3·050	0·003
	YEARDOI	-15·440	13·467	98·978	-1·147	0·254
	Time since Injury*YEARDOI	12·064	13·812	219·579	0·874	0·383
AIS-C (n = 106)	Intercept	46·755	6·416	143·588	7·287	<0·001
	Age	-6·499	8·855	104·395	-0·734	0·465
	Time since Injury	41·567	4·821	440·582	8·622	<0·001
	YEARDOI	9·198	9·649	184·149	0·953	0·342
	Time since Injury*YEARDOI	-9·498	9·388	441·370	-1·012	0·312
AIS-D (n = 23)	Intercept	57·386	9·904	29·985	5·795	<0·001
	Age	4·259	15·182	20·289	0·281	0·782
	Time since Injury	35·681	7·763	101·811	4·597	<0·001
	YEARDOI	29·203	20·105	40·125	1·453	0·154
	Time since Injury*YEARDOI	-23·775	21·030	104·144	-1·131	0·261

Additional File 3: Table S9. Sensitivity analysis: Surgical timing on total motor score stratified by AIS grades. Patients were enrolled in the Sygen Trial. Note: In case the number of patients per group were too low (n<3), the model did not converge and thus, the results are not represented in the table.

	**AIS A**		**AIS C**		**AIS D**	
Predictors	*Estimates*	*p*	*Estimates*	*p*	*Estimates*	*p*
(Intercept)	47.34	**<0.001**	40.44	**<0.001**	50.96	**<0.001**
Age	-0.05	0.495	-0.10	0.410	-0.24	0.166
Sex [Male]	-0.84	0.733	2.40	0.498	8.09	0.327
Time	0.15	**<0.001**	0.42	**<0.001**	0.28	**<0.001**
Plegia [tetra]	-28.78	**<0.001**	-11.68	0.150	-17.63	0.147
ealy_vs_late [late]	-0.48	0.812	-6.81	0.410	3.51	0.525
**Random Effects**						
σ^2	20.65		55.72		47.36	
τ_{00}	116.07 $_{ID}$		82.91 $_{ID}$		44.48 $_{ID}$	
ICC	0.85		0.60		0.48	
N	128 $_{ID}$		45 $_{ID}$		14 $_{ID}$	
