# Supplementary material for: International surveillance study in acute spinal cord injury confirms viability of multinational clinical trials
Source: BMC Med. 2022 Jun 14;20:225. doi: 10.1186/s12916-022-02395-0 (PMC9202190; doi:10.1186/s12916-022-02395-0)
Supplement: Supplementary file 4 — Additional file 4: Figure S1. Epidemiological tab in Neurosurveillance. The user can interactively explore the changes in the epidemiological landscape in the EMSCI and Sygen data sets. The platform allows the user to customize the selection of patients to visualize. Figure S2. Epidemiological tab in Neurosurveillance. The user can interactively explore the changes in the epidemiological landscape in the EMSCI and Sygen data sets. The platform allows the user to customize the selection of patients to visualize. [file 12916_2022_2395_MOESM4_ESM.pdf]

Additional File 4

Software Implementation

The *Neurosurveillance* platform was developed using the free statistical software in R and the R package *Shiny*. Briefly, *Shiny* is an R package (and framework) that offers the ability to develop interactive web apps straight from within R. The resulting applications can be run locally or deployed online. Last is particularly beneficial to show and communicate updated findings to a broad audience.

The *Shiny* framework has two structures, contain a server.R file and ui.R file. The server file is a set of instructions that build the R components while the user-interface file is a set of instructions to display the application. R *Shiny* uses "reactive programming", which ensures that changes in inputs are immediately reflected in outputs, making it possible to build a highly interactive tool. Within the R *Shiny* package, ordinary controllers or widgets are provided for ease of use for application programmers.

Both, the front- and back-end of *Neurosurveillance* have been built using the *shinydashboard* package.[13] *shinydashboard* is based upon AdminLTE (https://adminlte. io/), an open-source admin control panel built on top of the Bootstrap framework (Version 3.x) and released under the MIT license. *Neurosurveillance* is available as an online application and is hosted at https://jutzelec.shinyapps.io/neurosurveillance/ and can be accessed via any web browser on any device (e.g., desktop computers, laptops, tablets, smartphones). *Neurosurveillance* is published under the BSD 3-Clause License. The source code of *Neurosurveillance* is available through Github at https://github.com/jutzca/Neurosurveillance/

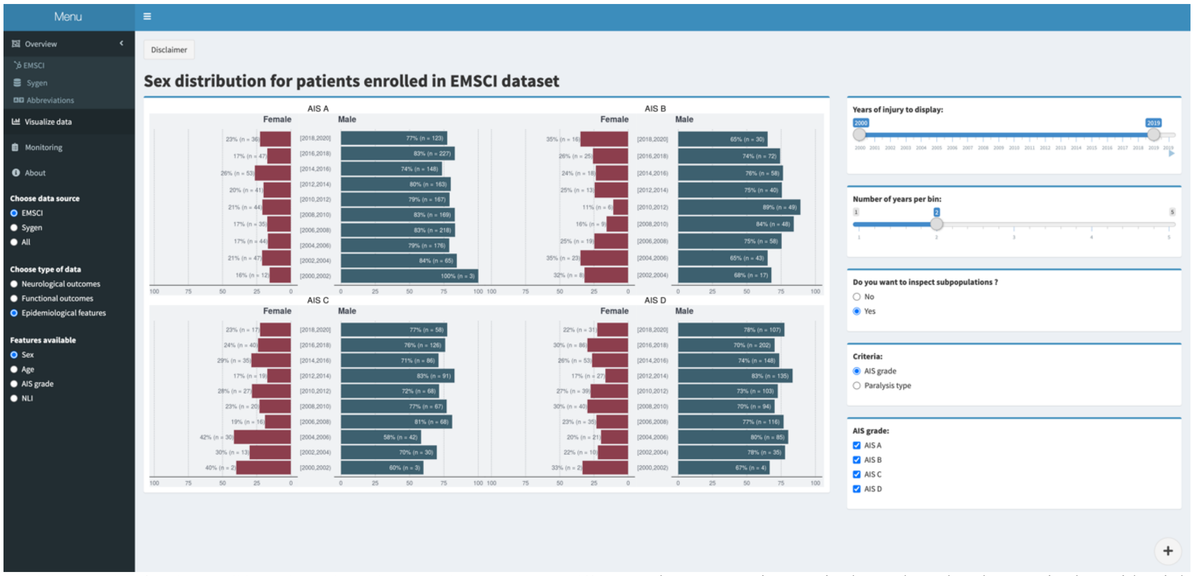

Additional File 4: Figure S1. Epidemiological tab in *Neurosurveillance*. The user can interactively explore the changes in the epidemiological landscape in the EMSCI and Sygen data sets. The platform allows the user to customize the selection of patients to visualize.

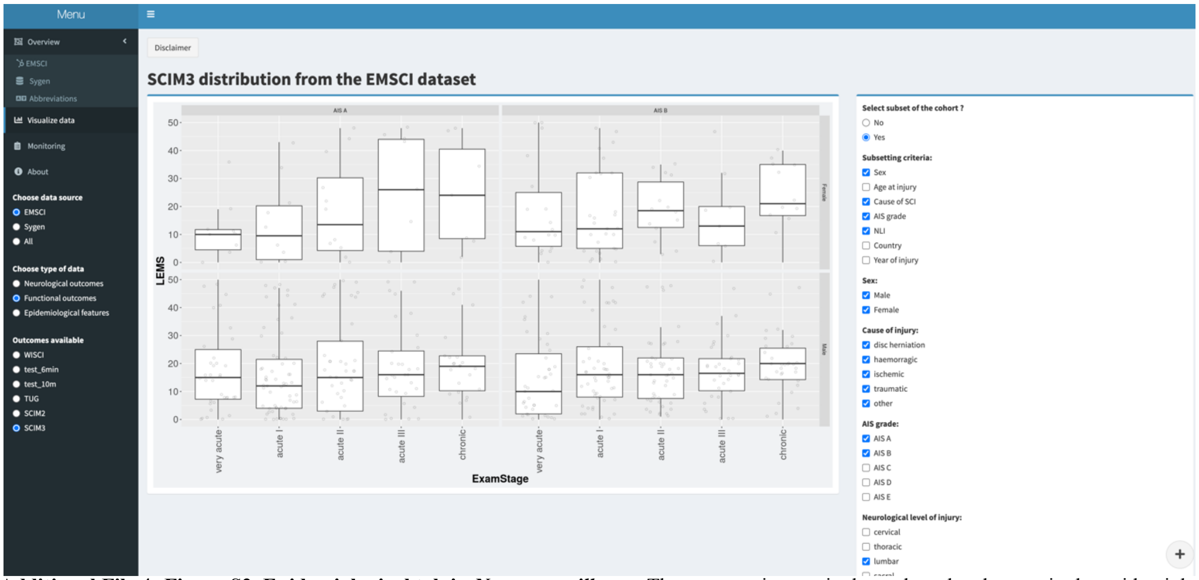

Additional File 4: Figure S2. Epidemiological tab in *Neurosurveillance*. The user can interactively explore the changes in the epidemiological landscape in the EMSCI and Sygen data sets. The platform allows the user to customize the selection of patients to visualize.
